# Assembly of the Complete Mitochondrial Genome of Chinese Plum (*Prunus salicina*): Characterization of Genome Recombination and RNA Editing Sites

**DOI:** 10.3390/genes12121970

**Published:** 2021-12-10

**Authors:** Bo Fang, Jingling Li, Qian Zhao, Yuping Liang, Jie Yu

**Affiliations:** 1 Fruit Research Institute, Chongqing Academy of Agricultural Sciences, Chongqing 401329, China; fb0211@hotmail.com (B.F.); cqnkyzhaoqian@163.com (Q.Z.); 2 College of Horticulture and Landscape Architecture, Southwest University, Chongqing 400716, China; lijingling1997@163.com; 3 Key Laboratory of Horticulture Science for Southern Mountainous Regions from Ministry of Education, Chongqing 400716, China; 4 College of Soil and Water Conservation, Beijing Forestry University, Beijing 100083, China; id_19920603@163.com

**Keywords:** plum, *Prunus salicina*, mitogenome, genome recombination, RNA editing

## Abstract

Despite the significant progress that has been made in the genome sequencing of *Prunus*, this area of research has been lacking a systematic description of the mitochondrial genome of this genus for a long time. In this study, we assembled the mitochondrial genome of the Chinese plum (*Prunus salicina*) using Illumina and Oxford Nanopore sequencing data. The mitochondrial genome size of *P. salicina* was found to be 508,035 base pair (bp), which is the largest reported in the Rosaceae family to date, and *P. salicina* was shown to be 63,453 bp longer than sweet cherry (*P. avium*). The *P. salicina* mitochondrial genome contained 37 protein-coding genes (PCGs), 3 ribosomal RNA (rRNA) genes, and 16 transfer RNA (tRNA) genes. Two plastid-derived tRNA were identified. We also found two short repeats that captured the *nad*3 and *nad*6 genes and resulted in two copies. In addition, nine pairs of repeat sequences were identified as being involved in the mediation of genome recombination. This is crucial for the formation of subgenomic configurations. To characterize RNA editing sites, transcriptome data were used, and we identified 480 RNA editing sites in protein-coding sequences. Among them, the initiation codon of the *nad*1 gene confirmed that an RNA editing event occurred, and the genomic encoded ACG was edited as AUG in the transcript. Combined with previous reports on the chloroplast genome, our data complemented our understanding of the last part of the organelle genome of plum, which will facilitate our understanding of the evolution of organelle genomes.

## 1. Introduction

Plum (*Prunus salicina*) belongs to the genus *Prunus* of Rosaceae and is an important stone fruit tree species worldwide, as the species encompasses many fruit trees with important economic value, such as plum, apricot, peach, and so on [1]. China has become the world’s largest producer of plums, which are cultivated in almost every area of the country, except for the Qinghai–Tibet Plateau [2]. Plum fruit tastes sweet and is rich in anthocyanins, carotene, and phenolic compounds [3,4,5]; it is loved by consumers. In addition to direct consumption, plums can also undergo food processing; the fruit can be processed into dried fruit, canned fruit, juice, jam, etc. [6,7,8,9]. Furthermore, some species have been bred as ornamental trees and their flowers and leaves are of ornamental value, so plum trees are also used in landscaping. China has abundant genetic resources of plums. Plums have a cultivation history of more than 4000 years in China. Many cultivars have been cultivated and domesticated for a long time. Different varieties of plums have many differences in fruit size, color, flavor, aroma, and texture. Moreover, plums can hybridize with some related species, such as peach and apricot [10,11], and give rise to many varieties with different ploidy [12], so plum breeding is also of much interest to fruit tree breeders [7,13].

Mitochondria are semi-autonomous organelles that contain a genetic system independent of the nuclear genome [14], which are called mitochondrial genomes (mitogenomes), although many functions need to be regulated by genes encoded by the nucleus [15,16]. In contrast to animal mitochondrial genomes, plant mitochondrial genomes are very different in size and genomic configuration [17]. Usually, mitogenomes take the form of circular double-linked molecules, but several independent chromosomes have been reported [18], sometimes even being shown to exist in linear or multi-branched structures [19]. The special repair mechanism makes it easy for foreign DNA sequences to be inserted into the mitochondrial genome, resulting in a large number of repeated sequences [20,21]. These repeats often act as mediators in genome recombination, resulting in different genomic configurations [22,23]. These complex structures make the sequencing and assembly of mitochondrial genomes more difficult than in plastids. Therefore, the plants contain far fewer mitochondrial genome resources than plastids. In fact, there are fewer published mitochondrial genome sequences than those of nuclear genomes [24], mainly because the genes of plant mitochondria are highly conserved, and the low evolution rate and relatively complex structure limit phylogenetic studies based on mitochondrial DNA [25]. Furthermore, conventional Illumina sequencing is usually difficult to complete with the assembly of plant mitochondrial genomes due to the read length limitation. Therefore, it is not cost-effective to complete the sequencing of a plant mitochondrial genome. However, mitochondrial genomes are an important material in plant breeding because plant mitochondria contain cytoplasmic male sterility (CMS) genes encoded by mitochondria, which are involved in the production of functional pollen or functional male reproductive organs [26,27]. In recent years, the popularity of long-read sequencing presented by Pacbio and Oxford Nanopore has made the sequencing of plant mitochondrial genomes more convenient and economical. 

Genome sequencing of the genus *Prunus* was first carried out several years ago, and several genomic resources, including that of plum, peach, apricot, and some wild species, have been published [2,28], which has greatly enriched the genetic resources of plum varieties and provided rich genetic data for breeders. However, there have been few systematic studies on mitochondrial genomes in *Prunus*. So far, the mitochondrial genome resources of only one species in *Prunus* have been published and have not yet been discussed in detail [29]. There are only nine mitogenomes available on the National Center for Biotechnology Information (NCBI) in the family Rosaceae, including *Rosa chinensis*, *Fragaria orientalis*, *Malus domestica*, *Malus hupehensis var. mengshanensis*, *Eriobotrya japonica*, *Pyrus betulifolia*, *Sorbus aucuparia*, *Sorbus torminalis*, and *Prunus avium*. This has greatly limited our research on the evolution of mitochondrial genomes in Rosaceae. 

In this study, we completed mitochondrial genome sequencing of a plum cultivar, namely the Wushan plum, and subsequently characterized its genome structure, repeat sequence, and genome recombination, and compared it with that of other mitogenomes of Rosaceae. Combined with transcriptome data, we also extensively described the RNA editing sites of the encoding genes of mitochondria. These results will facilitate our understanding of the evolution of the organelle genome in *Prunus* and lay a foundation for future breeding research. 

## 2. Materials and Methods

### 2.1. Plant Sampling and DNA Sequencing

We collected a representative variety of Chinese plum widely cultivated in Wushan County, Chongqing, which is also known as Wushan Plum, and vouchers (Wushan-Cuili-001) were preserved in the herbarium of the Chongqing Academy of Agricultural Sciences. 

The total genomic DNA was extracted using the Cetyltrimethylammonium Bromide (CTAB) method [30]. The same DNA sample was used for Illumina sequencing and Oxford Nanopore sequencing. For Illumina sequencing, a DNA library with an insert size of 350 bp was constructed using the NEBNext^®^ library building kit [31] and was sequenced by using the Hiseq Xten PE150 sequencing platform at Novogene (Beijing, China). Sequencing produced a total of 5.19 Gigabyte (Gb) raw data. Clean data were obtained using Trimmomatic [32]. For Oxford Nanopore sequencing, purified DNA was prepared for long-read sequencing following the protocol in the SQK-LSK109 genomic sequencing kit (ONT, Oxford, UK). The purified library was loaded into an R9.4 Spot-On Flow Cell (ONT), and Oxford Nanopore GridION × 5 sequencing was carried out for 48 h at Novogene (Beijing, China). In total, 15.49 Gb of sequence reads (1,153,570 reads) was obtained. The average read length of the filtered reads was 13.43 Kilobyte (kb) (N50 = 19.40 kb).

### 2.2. Assembly and Annotation of Mitogenomes

The Oxford Nanopore long reads were assembled into contigs using Nextdenovo with default parameters. Mitochondrial contigs were identified in each draft assembly by the Basic Local Alignment Search Tool (BLAST) program [33] using the mitochondrial genome sequences of *P. avium* (accession number: MT975322.1) as references. Based on the average depth and the number of matched hits, a self-looping contig was selected as the potential mitochondrial genome. To finalize the mitogenome sequence, the Illumina short-read data and nanopore long-read data were used for hybrid error correction with minimap2/miniasm [34,35], racon (v1.4.20), and pilon (v1.23) [36,37]. Burrows-Wheeler Aligner (BWA) tools and SAMtools (v0.1.19) were used to map all the raw reads (including short reads and long reads) to the assembled mitogenome sequences [38,39]. The near even coverage of the assembled genome with these reads supports the correctness of the assembly. The average depth of assembled mitogenomes was 178× (long reads), suggesting the high copy number of mitochondrial sequences in Wushan plum.

The mitogenome was annotated using GeSeq [40] with the reference mitogenome of *P. avium* (accession number: MT975322.1, released on 11 March 2021). The protein-coding genes (PCGs) were manually checked and edited using Apollo [41] if there were any problems. The genome map was drawn using OGDRAW [42]. All tRNA genes were confirmed using tRNAscan-SE [43] with the default settings.

### 2.3. Identification of Repeats and Repeat-Mediated Homologous Recombinations 

The online program REPuter [44] (https://bibiserv.cebitec.uni-bielefeld.de/reputer/ accessed on 6 November 2021) was used for the identification of repeat elements with the following settings: a hamming distance of three, a minimal repeat size of 30 bp, and the e-value was limited to less than 1 × 10^−5^. For the identified repeats, we extracted these repeat sequences based on their location in the mitogenome and then mapped the short reads onto these repeats. Subsequently, for the mapped short reads, we checked each of the repeats to explore whether they had multiple structures by using the visualization tool, Consed [45]. In this way, the evidence of recombination that was mediated by repeat sequences could be observed directly. A total of 19 pairs of repeats (which were mainly direct repeats and inverted repeats) were found to be multiple structures. We then extracted two repeat units of the repeats and their flanking 1000 bp regions and artificially generated two potentially alternative conformations. Finally, the Oxford Nanopore long reads were mapped to these major and alternative conformations of all 19 pairs of repeats to exclude multiple structures due to intracellular sequence transfer. Only reads that completely covered the repeats and the 1000 bp region of their flanks were counted. A total of nine pairs of repeats were thought to be involved in mediating the recombination of mitochondrial genomes.

### 2.4. RNA Extraction and Sequencing

Total RNA from the same plant samples was extracted using the RNAprep Pure Kit DP432 (TIANGEN Biotech Co., Ltd., Beijing, China), following the manufacturer’s instructions. All the RNA samples were assessed for their integrity using a Qsep1 instrument. To construct RNA libraries with the VAHTS mRNA-seq V3 Library Prep Kit for Illumina, 1 μg of total RNA was used. The procedure included polyA-selected RNA extraction, RNA fragmentation, random hexamer-primed reverse transcription, and 150 nt paired-end sequencing by Illumina HiSeq X-ten. Raw RNA-seq reads were processed to remove low-quality reads (reads of more than 50% of bases with a Q-value ≤ 20) together with reads composed of more than 5% of ambiguous nucleotides. Sequencing yielded 7.6 G of raw data.

### 2.5. Identification of RNA Editing Sites in Mitochondrial Protein-Coding Genes (PCGs)

In order to eliminate the interference of natural variation, based on the WGS data of other cultivars of *P. salicina* that we sequenced, we used BWA to map the raw reads to the sequences of mitochondrial PCGs to identify potential single-nucleotide polymorphism (SNP) sites. The results confirmed that the coding sequence of mitochondrial PCGs was highly conserved, and no SNP sites were found. Next, we mapped our transcriptome data to these PCGs, and the potential editing sites were extracted using SNP-calling in BCFtools v1.12 [46], ‘bcftools mpileup’ with stringent parameters “-Ou -f -I -d 10000 -B -q 20” and ‘bcftools call’ with parameters “-mv -o”. We also counted the coverage and base composition of each site using a custom script; sites with low coverage (less than 10) were not considered. RNA editing events were only retained if at least 10% of the reads were supported.

## 3. Results

### 3.1. Characteristics of the P. salicina Mitogenome

**Figure 1:**
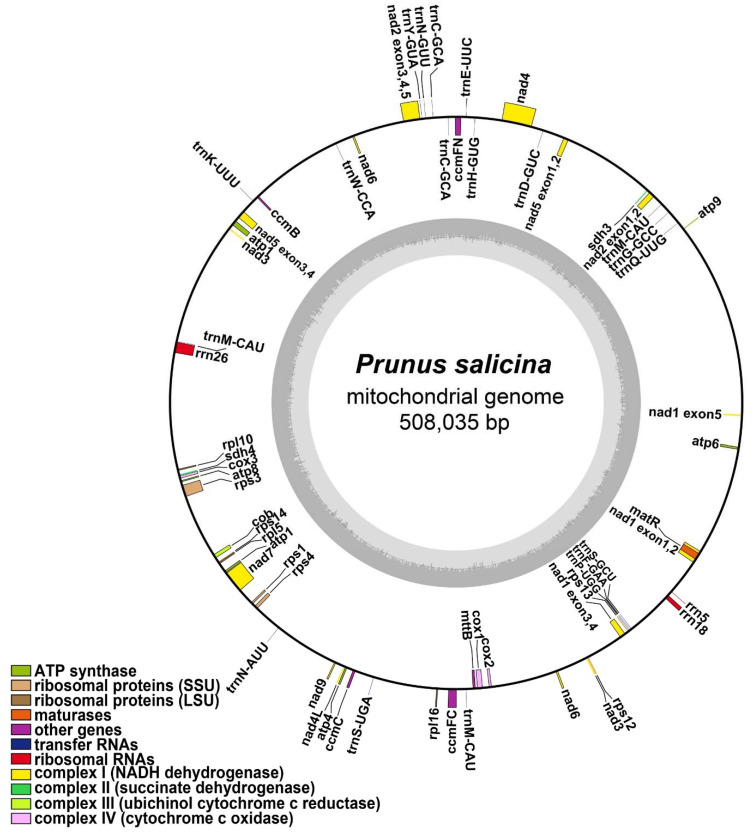
The circular map of *P. salicina* mitogenome. Genes belonging to different functional groups were color coded. ATP: adenosine-triphosphate; SSU: ribosomal small subunit; LSU: ribosomal large subunit; NADH: Nicotinamide adenine dinucleotide.

The *P. salicina* mitogenome was assembled into a single, circular, 508,035 base pair (bp) molecule (Figure 1), which was 63,453 bp longer than that of the *P. avium* (sweet cherry) previously reported. We mapped the raw data to the assembled genome, and both long reads and short reads confirmed that our assembly was gap-free. The mean coverage of *P. salicina* in the present study was 178× for long reads and 48× for short reads.

The mitochondrial genome contained 24 unique core genes and 11 unique variable genes (Table 1). These included five ATP synthase genes (*atp*1, *atp*4, *atp*6, *atp*8, and *atp*9); nine NADH dehydrogenase genes (*nad*1, *nad*2, *nad*3, *nad*4, *nad*4L, *nad*5, *nad*6, *nad*7, and *nad*9), four Cytochrome C Biogenesis genes (*ccm*B, *ccm*C, *ccm*Fc, and *ccm*Fn), three Cytochrome C oxidase genes (*cox*1, *cox*2, and *cox*3), three large subunit of ribosome proteins (*rpl*5, *rpl*10, and *rpl*16), four small subunit of ribosome proteins (*rps*1, *rps*3, *rps*4, *rps*12, *rps*13, and *rps*14), a transport membrane protein (*mtt*B), a maturase (*mat*R), Ubiquinol Cytochrome c Reductase (*cob*), and two respiratory genes (*shd*3 and *sdh*4). Notably, two genes (*atp*6 and *sdh*3) were found to lack a start codon and a stop codon, which could be pseudogenes. Furthermore, *nad*3 and *nad*6 each had two copies in this mitogenome. A total of 16 unique tRNAs and 3 rRNAs were annotated in the *P. salicina* mitogenome.

We also searched for homologous sequences between mitochondrial and plastid genomes and nuclear genomes based on the BLASTn program, and we retrieved a total of 14 homologous sequences between mitogenomes and plastomes (MTPTs) and 982 homologous sequences between mitogenomes and nuclear genomes (MTNUs). However, with regard to MTPTs, none of these sequences had a length of more than 1000 bp, and the total length was only 3116 bp, showing a lack of sequence migration between organelles in *P. salicina*. Among these limited homologous sequences, we annotated some gene fragments from plastid (Table S1), such as *pet*N, *psa*J, *psb*E, and *psb*C. Some tRNA sequences were also shown to have high homology. Among them, two tRNA (*trn*W-CCA and *trn*N-GUU) of mitochondrion might be plastid-derived, because they were shown to have 100% sequence similarity with this tRNA in the plastid (Table 1). For MTNUs, the maximum length of the identified homologous sequence was 8916 bp, with 100% identity. The sequence contained a partial exon of the *nad*7 gene. However, besides this, there were only four other homologous fragments with lengths of more than 1 kb (Table S2), suggesting that the mitogenomes have extremely short sequences acquired from the other two genetic systems in *P. salicina*.

### 3.2. Comparison of Mitochondrial Genomes in ROSACEAE

We searched the available mitochondrial genome resources of Rosaceae in the NCBI and retrieved the complete mitochondrial genome sequences of nine different species (Table 2). These genomes ranged in size from 275,143 to 508,035 and showed considerable variation; however, their GC content was similar, ranging from 45.21% to 45.62%. It is worth noting that multiple copies of some core protein-coding genes (PCGs) were found after re-annotation of these mitogenomes. In our case, eight out of ten mitogenomes had multiple-copy PCGs.

In this study, two mitochondrial genes of the *P. salicina* mitogenome, *nad*3, and *nad*6, had the coding regions of 618 bp and 357 bp, respectively. However, they were observed to be located in two short, inverted repeats with lengths of only 666 bp and 511 bp. It is rare for PCGs to be located on such short repeats, and in this case, they seemed to have been formed in order to obtain multiple copies. In contrast, repeated sequences of other mitochondrial genomes are much longer. For example, three mitochondrial genes (*atp*8, *cox*2, and *rps*3) in *Malus hupehensis var. mengshanensis* were found to be located in a pair of large forward repeats with a length of 30,444 bp [47]. In addition, the four genes (*atp*1, *nad*6, *rps*1, and *rps*4) in the *Pyrus betulifolia* mitogenome are located in a pair of inverted repeats over 30,000 in length [48]. In *Sorbus aucuparia*, the double copies of *mat*R were found to be associated with the inverted repeats of a length more than 26,000 bp.

**Figure 2:**
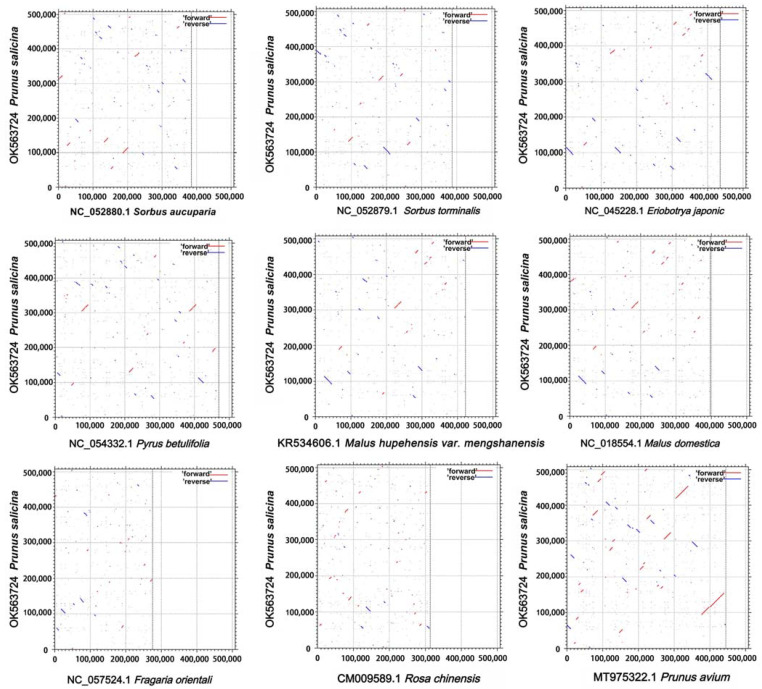
Dot-plot graphs indicating regions of synteny between mitochondrial genomes in Rosaceae compared to *P. salicina* as the reference.

We also compared collinearity between the published mitochondrial genomes of Rosaceae to assess genome rearrangement between different lineages; in the case of our *P. salicina* mitogenome as a reference, the dot-plot (Figure 2) analyses only showed short stretches (less than 22 kb) of synteny across all species, except for *P. avium*, among which, the stretches of synteny with *Fragaria orientalis* and *Rosa chinensis* (both belonging to the subfamily of Rosoideae) were the lowest, and the maximum was less than 12 kb. Regarding other species belonging to the subfamily of Amygdaloideae, *P. salicina* and *P. avium* had the two largest stretches of synteny, being about 62 kb and 40 kb, respectively. This result indicated that the mitogenomes underwent extensive rearrangement and that the mitochondrial genomic synteny decays with time from the divergence.

### 3.3. Repeats and Homologous Recombinations

**Figure 3:**
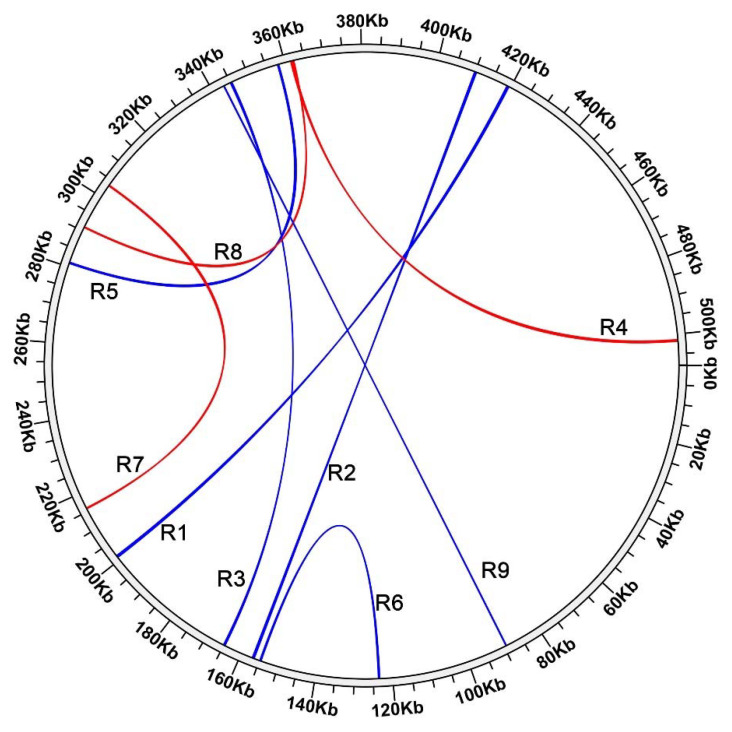
The distribution of the nine pairs of repeats which could mediate recombination in *P. salicina* mitogenome. The arcs show the linkage of direct (red) and inverted (blue) repeats (R1 to R9) with evidence for recombination activity. Numbers on the outer circle represent genome coordinates (Kb).

In addition to increasing the mitogenome size, the presence of repeats also promoted genome recombination as a good medium. Based on the online program REPuter, we identified 684 dispersed repeats in the *P. salicina* mitogenome, most of which were shown to be forward and palindromic (inverted) repeats (Table S3). The length of these repeats mainly ranged from 30 bp to 200 bp, and only 14 pairs of repeats exceeded 200 bp. Based on their position in mitogenomes, we extracted these repeats and mapped the Illumina short reads to these repeats to identify whether there were multiple structures/extensions at both ends. A total of 19 pairs of short repeats were found to have multiple structures at both ends that may facilitate the formation of isomers. However, the detection based on short reads may contain false positives. We further mapped the long reads to these repeats to further screen for repeat units that may promote genome recombination. Finally, a total of nine pairs of repeats showed clear evidence of recombination activity under our stringent criteria (Table 3 and Figure 3). The length of these repeats ranged from 100 bp to 500 bp, with one to eleven recombined reads for each repeat.

**Figure 4:**
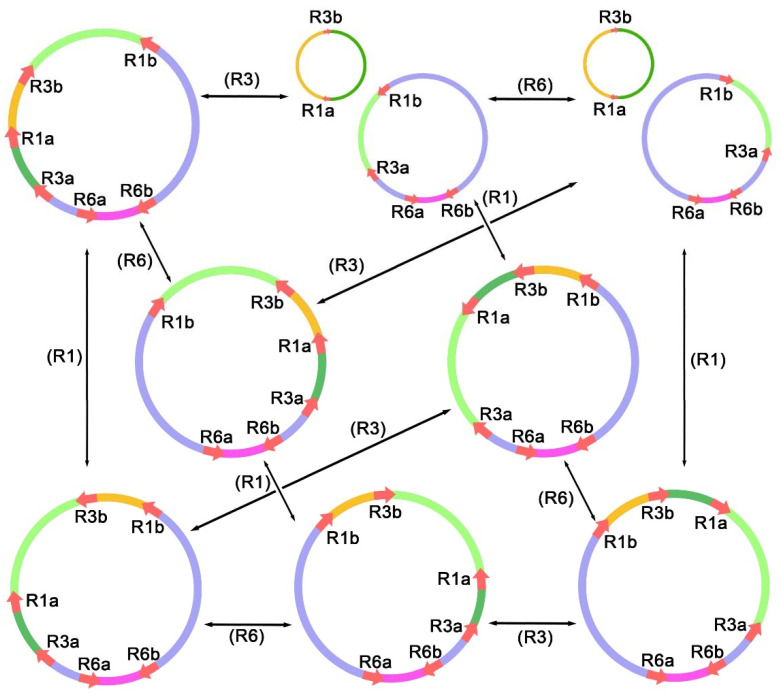
The possible configurations generated from intramolecular recombination mediated by repeat sequences. Here, we only describe the potential isomers generated from the three pairs of repeats with the highest recombination frequency. The three pairs of repeat sequences are R1, R3, and R6, as shown in Table 2.

Three direct repeats and six inverted repeats recombined at frequencies ranging from 0.55% to 5.70%, which could give rise to a set of alternative configurations of mitogenomes and subgenomes via inversions and subdivisions of the master conformations (Figure 4). The highest frequency of recombination was a pair of 511 bp inverted repeats, with a total of 11 pairs of recombinant reads supporting alternative conformations. However, 182 long reads supported the master circle conformation, suggesting a lower frequency of recombination. As for the remaining repeats, they recombined the mitogenome at a lower frequency, similar to the results previously observed in *Scutellaria tsinyunensis* and *Nymphaea colorata* [49,50], namely that the configuration of the master circle was overwhelmingly dominant in the mitochondrion. Our results also support previous studies that showed that the frequency of recombination mediated by short repeats tends to be lower than that mediated by long repeats, with the isomers mediated by this being closer to equal proportions. Notably, this 511 bp repeat captured the *nad*3 gene; it is not known whether genes in the repeated region are affected in expression and transcription.

### 3.4. Characteristics of RNA Editing Sites in Mitochondrial PCGs

**Figure 5:**
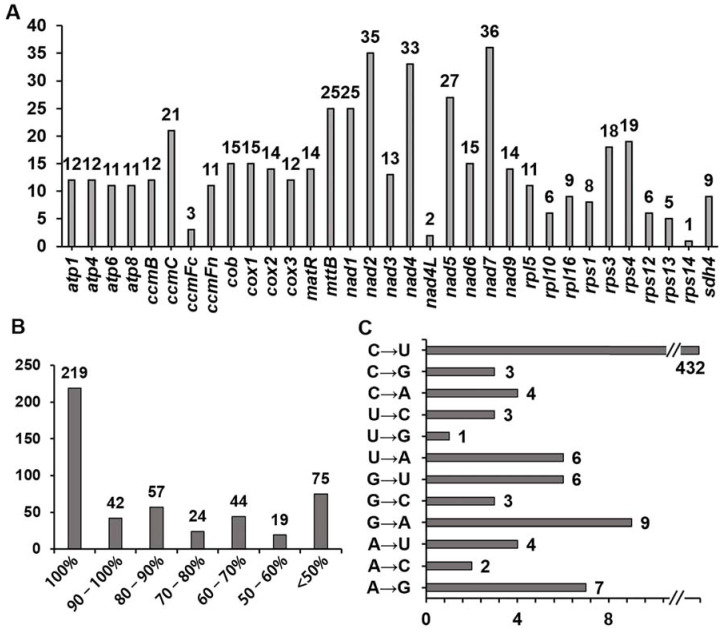
Characteristics of the RNA editing sites identified in PCGs of *P. salicina* mitogenome. (**A**) The number of RNA editing sites identified in each PCGs. (**B**) RNA editing efficiency. (**C**) RNA editing type and their number identified in all PCGs.

We focused on RNA editing sites in 34 coding sequences of mitochondrial genes in Wushan plums, as these sites are likely to trigger changes in coding amino acids. This is abnormally important for the function of mitochondrial genes. From our data, we identified 480 RNA editing sites (Table S4), which is a similar number to most flowering plants [51,52]. Among them, three genes (*nad*7, *nad*2, and *nad*4) had more than 30 editing sites (Figure 5A). It is worth noting that over 45% (219) of the editing sites had near 100% editing efficiency, and 405 (84.4%) were edited with an efficiency above 50%, while only 75 (15.6%) were edited with an efficiency below 50% (Figure 5B). Overall, the editing process was much more efficient than we expected.

Furthermore, we identified a total of 12 different types of RNA editing; of these, 90% (432) were C to U editing (Figure 5C). This type of editing was found in almost all protein-coding genes. U to G and A to C editing were the least common types, with only one (found in the *cox*3 gene) and two (found in the *atp*1 and *atp*8 gene) types being found, respectively. Besides these, the number of occurrences of other types of editing ranged from three to nine.

In some cases, RNA editing was also reported to be responsible for the creation of initiation and/or termination codons. However, while most genes in our study have common start or stop codons, we observed that the start codon of *nad*1 appears to be associated with an RNA editing event. The gene on the negative strand was recorded in the genome as ACG, but the base C to U editing was confirmed in four of the six transcripts. Roughly 66.67% C-to-U-editing efficiency was recorded in our *nad*1 gene. However, no RNA editing event was detected in another gene, *rps*4, in which ACG is also used as a translation initiation (Figure S1). Other genes, such as *rpl*16 encoded by a mitogenome, whose translation begins with genome-encoded GUG, showed no evidence of RNA editing in transcriptome data. This is probably normal in the organelle genomes, and this initial codon was commonly found in the *rps*19 gene encoded in the plastid genomes. This was also confirmed in the bacterial *rpl*16 gene [53].

## 4. Discussion

For organelle genomes, the capture of multi-copy genes is closely related to the formation of repeats, especially in plastomes. Typically, plastomes have a pair of inverted repeats (IRs) and are usually from 20 kb to 30 kb in length. Thus, genes in the IR region, such as *ndh*A, *ndh*B, *rps*7, *rps*12, etc., obtained double copies. This is also not uncommon in plant mitogenomes. The migration of foreign sequences and the special repair mechanism of plant mitogenomes generated a large number of repeats [54,55]. This not only increases the mitogenome size but also allows some mitochondrial genes to obtain multiple copies. However, due to the small number of conserved core mitochondrial genes, the distribution of mitochondrial genes is relatively sparse. Therefore, it is easier to obtain multiple copies for genes located in some large, repeated regions [56]. In the published mitogenomes of Rosaceae, we found the genes that obtained multiple copies included some core genes, such as *atp*8, *cox*2, *nad*4, *atp*1, *nad*6, *nad*3, and *mat*R, as well as some variable genes, such as *rps*1, *rps*3, *rps*4, and *rps*12. Most of them were captured by large repeats, but a special case was observed in the Wushan plum mitogenome that we assembled here, in which the multi-copy event of *nad*3 and *nad*6 was associated with the two short repeats, which has hardly been reported before.

It has been reported that the size of repeats is closely related to the frequency of recombination [56]; namely, the frequency of recombination mediated by short repeats tends to be lower than that mediated by long repeats, the isomers mediated by which is closer to equal proportions. For large repeats, such as the typical inverted repeats observed in chloroplast DNA (cp DNA), it was previously thought that equal proportions mediate SSC region recombination [57]. Here, all of the repeats identified in the mitogenome of plum are short repeats, and they all have low recombination frequency, which are findings that are consistent with those previously reported [49,50]. However, organelles tend to have high copy numbers. It has been reported that the copy number of organelle genomes varies in different tissues of plants and at different developmental stages. For example, the copy number of mitochondrial DNA in roots was found to be more than that in the leaves of two maize lines. With the development of leaves and roots, the copy numbers of plastid DNA increased, while it was decreased for mitochondrial DNA [58]. Furthermore, whole-genome duplications would also induce elevated organellar genome copy numbers [59]. In conclusion, given the high copy number of organelles, the presence of isomers of mitogenomes is considerable, despite the low frequency of recombination. In our study, we made configurations mediated by the three pairs of repeats with the highest recombination frequency, including the rearrangement of mitogenomes, and possibly even the divided genomic conformation. In reality, the process is more complicated than that. As observed in melon, the leaf cells of melon do not contain sufficient copies of mitochondrial genes to ensure that every mitochondrion possesses the entire mitochondrial genome. Thus, the divided subgenome might be more common in plants [60]. In our study, we confirmed the existence of alternative conformations, and previously, this low-frequency recombination has been reported to have certain effects on plant growth, such as the induction of cytoplasmic male sterility (CMS) [61,62].

RNA editing events are ubiquitous in plant mitochondrial genomes. They include not only the coding region but also a large number of non-coding regions [56]. The most widespread type of RNA editing event is the single-base transition of post-transcriptional regulation. In particular, for mitochondrial genes, it has been reported that the initiation codon of many genes might be generated by RNA editing events. For example, previous studies regarding potatoes have found that the genome-encoded ACG has been identified as a C-to-U, RNA-editing event, as the translation is initiated for the *cox*1 gene encoded by potato mitochondria [63]. However, in another potato mitochondrial gene, *rps*10, ACG has been identified, as the translation is initiated directly without RNA editing [64]. Here, we also found this phenomenon in the mitogenome of plum. That is, our transcriptome data confirmed that the actual initial codon of the *nad*1 gene was most likely edited from ACG to AUG, but no editing event was found in the initial codon (ACG) of the mitochondrial *rps*4 gene. We speculate that ACG and AUG may be compatible in the initiation of translation, and this compatibility is reflected in the efficiency of RNA editing, and there may be a mechanism that influences the editing efficiency of the initial codon. Of course, we emphasize that the RNA editing sites identified here may not be complete, as some of the mitochondrial encoding genes lack adequate coverage, which may be due to low expression levels or a small amount of sequencing data.

## 5. Conclusions

The use of a mixture of long reads and short reads made it possible to accurately assemble plant mitochondrial genomes with limited homology. In this study, we assembled the complete mitochondrial genome of a Chinese plum cultivar, Wushan Plum, using Illumina short reads and Oxford nanopore long reads. Based on the long-read data, we identified nine pairs of short repeats that could mediate the genome recombination. Although their recombination frequency is mostly less than 5%, we speculate that abundant subgenomic configurations are still present in the mitochondrial genome of plums. Notably, one pair of repeats resulted in two copies of the *nad*3 gene. Subsequently, we used transcriptome data to characterize the RNA editing sites of mitochondrial protein-coding sequences in detail and found abundant editing sites, especially from C to U editing. These include the initiation site of translation in the *nad*1 gene. Considering the plastid and nuclear genomes of *P. salicina* have been previously published, the sequencing of the mitochondrial genome of *P. salicina* enabled us to provide a broader perspective for the study of gene dialogue between nuclear, mitochondrial, and plastid genomes. The mitochondrial genome we provided here is of great significance for the study of cytoplasmic male sterility in Prunus and also has enriched the mitochondrial genome resources of Rosaceae and deepened our understanding of organelle genome evolution. Furthermore, the decipherment of all genetic resources of *P. salicina* is a milestone in the study of the species.

## Figures and Tables

**Table 1:** Gene composition in the mitogenome of *P. salicina*.

Group of Genes		Name of Genes
Core genes	ATP synthase	*atp*1, *atp*4, *atp*6 ^1^, *atp*8, *atp*9
	Cytochrome c biogenesis	*ccm*B, *ccm*C, *ccm*Fc ^2^, *ccm*Fn
	Ubichinol cytochrome c reductase	*cob*
	Cytochrome c oxidase	*cox*1, *cox*2, *cox*3
	Maturases	*mat*R
	Transport membrane protein	*mtt*B
	NADH dehydrogenase	*nad*1 ^2^, *nad*2 ^2^, *nad*3 (×2), *nad*4 ^2^, *nad*4L, *nad*5 ^2^, *nad*6 (×2), *nad*7 ^2^, *nad*9
Variable genes	Large subunit of ribosome	*rpl*5, *rpl*10, *rpl*16
	Small subunit of ribosome	*rps*1, *rps*3 ^2^, *rps*4, *rps*12, *rps*13, *rps*14
	Succinate dehydrogenase	*sdh*3 ^1^*, sdh*4
rRNA genes	Ribosomal RNAs	*rrn*5, *rrn*18, *rrn*26
tRNA genes	Transfer RNAs	*trn*Y-GUA, *trn*W-CCA ^3^, *trn*S-UGA, *trn*S-GCU, *trn*Q-UUG, *trn*P-UGG, *trn*N-GUU, *trn*N-AUU ^3^, *trn*M-CAU (×3), *trn*K-UUU, *trn*H-GUG, *trn*G-GCC, *trn*F-GAA, *trn*E-UUC, *trn*D-GUC, *trn*C-GCA (×2)

^1^ Pseudogenes, ^2^ genes that contain introns, ^3^ Genes that derived from plastid.

**Table 2:** Comparison of mitogenomes among 10 Rosaceae species.

Species	Accession Number	Genome Size (bp)	GC Content (%)	Number of PCGs		Multicopy Genes
				Core Genes	Variable Genes	
*Rosa chinensis*	CM009589.1	313,448	45.48	24	9 (1)	*rps*3
*Fragaria orientalis*	NC_057524.1	275,143	45.24	24	7	
*Malus domestica*	NC_018554.1	396,947	45.39	24	9 (1)	*rps*12
*Malus hupehensis var. mengshanensis*	KR534606.1	422,555	45.21	24 (2)	9 (1)	*atp*8, *cox*2, *rps*3
*Eriobotrya japonica*	NC_045228.1	434,980	45.42	24 (1)	10	*nad*4
*Pyrus betulifolia*	NC_054332.1	469,928	45.28	24 (2)	10 (2)	*atp*1, *nad*6, *rps*1, *rps*4
*Sorbus aucuparia*	NC_052880.1	384,977	45.39	24 (1)	9	*mat*R
*Sorbus torminalis*	NC_052879.1	386,758	45.31	24	9	
*Prunus avium*	MT975322.1	444,582	45.62	24	10 (1)	*rps*3
*Prunus salicina*	OK563724.1	508,035	45.43	24 (2)	10	*nad*3, *nad*6

The numbers in parentheses represent the number of genes with multiple copies, which were listed in the rightmost column of the table. ‘bp’ represents base pair; ‘PCGs’ represents protein-coding genes.

**Table 3:** Recombination frequency of the mitochondrial genome of *P. salicina* related to nine repeat pairs.

Repeat	Length (bp)	Direction	Position	Reads Support Master Circle Conformation	Reads Support Alternative Conformation
R1	511	+	200803–201313	182	11
		–	419510–419000	(94.30%)	(5.70%)
R2	472	+	156450–156921	167	4
		–	410364–409893	(96.67%)	(2.33%)
R3	393	+	164678–165070	135	4
		–	345427–345035	(97.12%)	(2.88%)
R4	385	+	361588–361972	166	3
		+	501441–501825	(98.22%)	(1.78%)
R5	299	+	280928–281226	163	4
		–	358365–358067	(97.60%)	(2.40%)
R6	294	+	123383–123676	149	5
		–	154864–154571	(96.75%)	(3.25%)
R7	195	+	215708–215902	176	5
		+	303436–303630	(97.24%)	(2.76%)
R8	175	+	290959–291133	182	1
		+	362216–362390	(99.45%)	(0.55%)
R9	111	+	89335–89445	171	1
		–	343225–343115	(99.42%)	(0.58%)

## Data Availability

The assembled mitogenome sequences have been deposited in NCBI (https://www.ncbi.nlm.nih.gov/ (accessed on 17 October 2021)) with accession number: OK563724. The raw sequencing data used in this experiment was also deposited in NCBI with the BioProject number PRJNA780372. All data generated by this study are available at the corresponding author upon reasonable request.

## Supplementary Materials

The following are available online at https://www.mdpi.com/article/10.3390/genes12121970/s1, Figure S1: RNA editing events of the initiation codon of *nad*1 gene. Transcriptome data were mapped to the genome and it was found that the ACG encoded by genome was partially edited to AUG in the transcripts (above). However, no such editing event was observed in *rps*4 (below). Table S1: The homologous sequences identified in the chloroplast and mitochondrial genomes of *P. salicina*. Table S2: The homologous sequences identified in the nuclear and mitochondrial genomes of *P. salicina*. Table S3: The dispersed repeats identified in the mitogenome of *P. salicina*. Table S4: RNA editing sites identified in mitochondrial PCGs from transcriptome data.
