# Supplementary material for: Assembly of the Complete Mitochondrial Genome of Chinese Plum (Prunus salicina): Characterization of Genome Recombination and RNA Editing Sites
Source: Genes (Basel). 2021 Dec 10;12(12):1970. doi: 10.3390/genes12121970 (PMC8701122; doi:10.3390/genes12121970)
Supplement: Supplementary file 1 [file genes-12-01970-s001.zip › genes-1491074 -supplementary.pdf]

Supplementary Materials

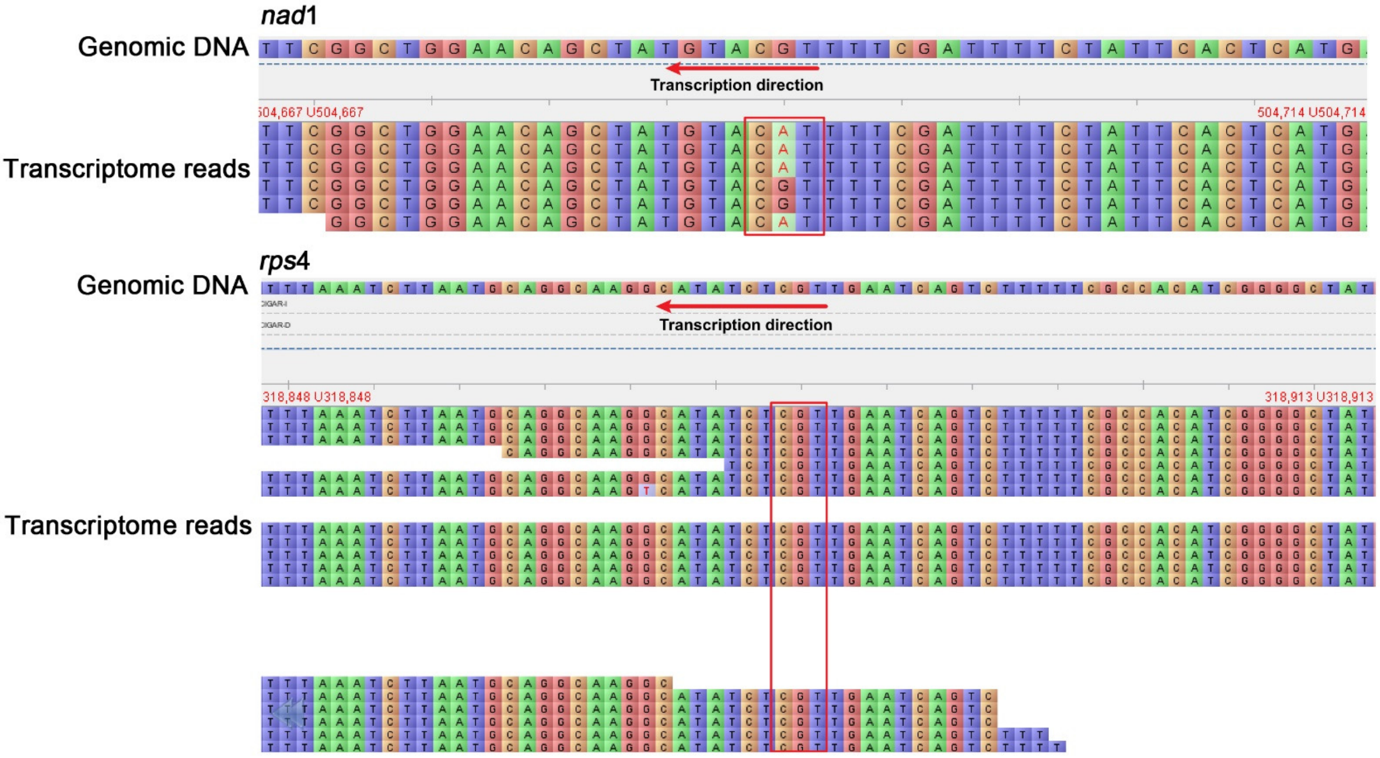

Figure S1. RNA editing events of the initiation codon of *nad*1 gene. Transcriptome data were mapped to the genome and it was found that the ACG encoded by genome was partially edited to AUG in the transcripts (above). However, no such editing event was observed in *rps*4 (below).

Table S1. The homologous sequences identified in the chloroplast and mitochondrial genomes of *P. salicina*.

**Fragments**	**Length (bp)**	Mitogenome		Plastome		Gene
		Start	End	Start	End	
1	859	447,571	448,429	103,084<br>140,159	103,947<br>141,022	*rrn*16 fragment
2	545	271,213	271,757	29,260	29,777	*pet*N fragment
3	418	162,102	162,519	68,420	68,815	*trn*P-UGG, *trn*W-CCA
4	325	287,671	287,995	66,525	66,846	*psa*E fragment
5	246	270,842	271,087	69,057	69,304	*psa*J fragment
6	185	101,877	102,061	32,101	32,289	*trn*D-GUC
7	148	2389	2536	36,618	36,762	*psb*C fragment
8	85	135,654	135,570	111,085<br>132,936	111,170<br>133,021	*trn*N-GUU
9	79	351,965	352,043	88,407<br>155,625	88,481<br>155,699	*trn*M-CAU
10	85	121,608	121,692	153	237	*trn*H-GUG
11	79	62,199	62,121	54,267	54,345	
12	62	408,289	408,350	105,740<br>138,305	105,801<br>138,366	
13	32	288,047	288,015	66,489	66,520	
14	31	3277	3307	10,849	10,879	

Table S2. The homologous sequences identified in the nuclear and mitochondrial genomes of *P. salicina*.

Query	Q. Start	Q. End	Subject	S. Start	S. End	Identity (%)	Alignment Length (bp)	Mismatches	Gap Opens	Evalue
mitogenome	314,461	323,376	chromosome 4	29,816,011	29,807,096	100	8,916	0	0	0.00×10^{1}
mitogenome	353,254	354,629	chromosome 8	6,912,615	6,913,990	98.256	1,376	24	0	0.00×10^{1}
mitogenome	354,834	355,980	chromosome 8	6,914,095	6,915,225	96.867	1,149	16	6	0.00×10^{1}
mitogenome	353,513	354,629	chromosome 8	6,637,339	6,636,225	96.329	1,117	39	2	0.00×10^{1}
mitogenome	354,903	355,980	chromosome 8	6,636,073	6,635,002	89.551	1,091	82	13	0.00×10^{1}
mitogenome	210,967	211,952	chromosome 6	24,034,047	24,033,062	100	986	0	0	0.00×10^{1}
mitogenome	322,544	323,376	chromosome 7	7,686,392	7,685,555	94.055	841	39	5	0.00×10^{1}
mitogenome	235,238	236,000	chromosome 1	50,438,700	50,439,462	99.345	763	5	0	0.00×10^{1}
mitogenome	306,423	307,134	chromosome 4	14,050,344	14,049,633	99.86	712	1	0	0.00×10^{1}
mitogenome	114,214	114,879	chromosome 5	12,355,019	12,354,354	99.7	666	2	0	0.00×10^{1}
mitogenome	241,577	242,237	chromosome 6	23,748,805	23,749,465	99.697	661	2	0	0.00×10^{1}
mitogenome	108,544	109,108	chromosome 2	35,863,777	35,863,213	100	565	0	0	0.00×10^{1}
mitogenome	143,745	144,289	chromosome 4	25,532,814	25,532,270	97.798	545	12	0	0.00×10^{1}
mitogenome	67,560	68,092	chromosome 4	14,106,713	14,107,245	99.812	533	1	0	0.00×10^{1}
mitogenome	475,566	476,090	chromosome 4	14,049,281	14,048,752	98.491	530	3	1	0.00×10^{1}
mitogenome	242,084	242,578	chromosome 4	14,048,749	14,048,255	100	495	0	0	0.00×10^{1}
mitogenome	321,754	322,232	chromosome 7	7,687,676	7,687,202	96.242	479	14	1	0.00×10^{1}
mitogenome	305,116	305,567	chromosome 4	17,533,895	17,533,447	90.066	453	40	4	4.81×10^{-162}
mitogenome	199,362	199,795	chromosome 4	17,533,895	17,533,464	90.575	435	37	3	2.90×10^{-159}
mitogenome	220,624	221,047	chromosome 1	50,440,508	50,440,931	100	424	0	0	0.00×10^{1}
mitogenome	213,308	213,725	chromosome 6	24,033,047	24,032,630	99.761	418	1	0	0.00×10^{1}
mitogenome	384,483	384,830	chromosome 5	28,485,970	28,486,338	78.125	384	33	19	7.32×10^{-46}
mitogenome	340,584	340,963	chromosome 4	18,150,794	18,151,174	89.529	382	37	3	1.81×10^{-131}
mitogenome	242,531	242,873	chromosome 6	23,750,113	23,750,455	99.417	343	2	0	2.83×10^{-174}
mitogenome	285,512	285,837	chromosome 1	53,328,086	53,327,773	81.818	330	40	6	9.21×10^{-65}
mitogenome	433,550	433,877	chromosome 4	13,473,278	13,472,951	99.088	329	1	2	2.87×10^{-164}
mitogenome	448,355	448,681	chromosome 1	19,319,356	19,319,030	100	327	0	0	1.03×10^{-168}
mitogenome	448,355	448,681	chromosome 1	23,369,048	23,368,722	100	327	0	0	1.03×10^{-168}
mitogenome	329,667	329,976	chromosome 3	30,778,313	30,778,622	99.355	310	2	0	6.27×10^{-156}
mitogenome	345,823	346,122	chromosome 1	33,241,387	33,241,088	98	300	6	0	1.06×10^{-143}
mitogenome	163,064	163,358	chromosome 1	33,241,094	33,241,387	96.949	295	8	1	2.32×10^{-135}
mitogenome	490,756	491,048	chromosome 5	4,609,632	4,609,924	98.976	293	3	0	8.22×10^{-145}
mitogenome	6,672	6,957	chromosome 5	4,722,103	4,722,388	97.213	287	6	2	1.40×10^{-132}
mitogenome	421,196	421,481	chromosome 2	1,072,989	1,072,704	100	286	0	0	6.36×10^{-146}

mitogenome	102,050	102,326	chromosome 8	9,867,096	9,866,821	99.278	277	1	1	4.98×10^{-137}
mitogenome	322,280	322,553	chromosome 1	14,160,783	14,160,517	89.855	276	17	7	2.47×10^{-90}
mitogenome	376,220	376,490	chromosome 7	8,516,170	8,515,900	100	271	0	0	1.39×10^{-137}
mitogenome	457,062	457,321	chromosome 4	2,711,185	2,711,444	93.462	260	17	0	4.05×10^{-103}
mitogenome	457,062	457,321	chromosome 4	2,732,663	2,732,922	93.077	260	18	0	1.88×10^{-101}
mitogenome	151,201	151,454	chromosome 1	43,275,543	43,275,793	96.471	255	4	4	5.16×10^{-112}
mitogenome	110,606	110,858	chromosome 4	14,051,038	14,051,290	100	253	0	0	1.41×10^{-127}
mitogenome	440,303	440,552	chromosome 5	18,797,242	18,797,490	99.2	250	1	1	5.09×10^{-122}
mitogenome	100,764	101,012	chromosome 4	9,397,983	9,398,231	99.598	249	1	0	1.09×10^{-123}
mitogenome	162,991	163,238	chromosome 8	3,730,602	3,730,362	89.919	248	18	2	6.97×10^{-81}
mitogenome	151,217	151,454	chromosome 1	42,037,533	42,037,297	97.899	238	4	1	2.40×10^{-110}
mitogenome	378,842	379,079	chromosome 3	27,710,402	27,710,639	100	238	0	0	3.06×10^{-119}
mitogenome	151,218	151,454	chromosome 1	18,469,758	18,469,522	97.046	237	7	0	5.20×10^{-107}
mitogenome	151,218	151,454	chromosome 1	18,514,848	18,514,612	97.046	237	7	0	5.20×10^{-107}
mitogenome	151,218	151,454	chromosome 1	20,609,817	20,610,053	97.046	237	7	0	5.20×10^{-107}
mitogenome	448,672	448,907	chromosome 7	8,663,282	8,663,047	99.153	236	2	0	8.58×10^{-115}
mitogenome	270,747	270,978	chromosome 2	16,943,708	16,943,477	100	232	0	0	6.63×10^{-116}
mitogenome	270,747	270,978	chromosome 2	12,214,730	12,214,958	97.414	232	3	2	8.70×10^{-105}
mitogenome	392,451	392,681	chromosome 1	3,957,816	3,958,046	99.567	231	1	0	1.11×10^{-113}
mitogenome	392,451	392,681	chromosome 1	5,765,263	5,765,493	98.701	231	3	0	2.40×10^{-110}
mitogenome	319,980	320,206	chromosome 1	55,304,994	55,304,767	92.544	228	16	1	8.95×10^{-85}
mitogenome	54,239	54,462	chromosome 1	49,767,634	49,767,409	92.478	226	15	2	1.16×10^{-83}
mitogenome	381,778	382,003	chromosome 3	26,179,533	26,179,308	100	226	0	0	1.44×10^{-112}
mitogenome	54,239	54,462	chromosome 1	9,200,308	9,200,084	94.667	225	11	1	1.91×10^{-91}
mitogenome	54,239	54,461	chromosome 1	49,819,403	49,819,181	93.274	223	15	0	6.92×10^{-86}
mitogenome	322,224	322,442	chromosome 7	7,686,610	7,686,391	95	220	10	1	2.47×10^{-90}
mitogenome	118,830	119,049	chromosome 2	27,939,906	27,940,125	99.545	220	1	0	1.45×10^{-107}
mitogenome	221,032	221,248	chromosome 1	50,442,018	50,442,232	98.618	217	1	1	5.24×10^{-102}
mitogenome	386,751	386,963	chromosome 8	8,255,333	8,255,121	100	213	0	0	2.42×10^{-105}
mitogenome	466,757	466,969	chromosome 3	24,585,860	24,585,648	98.592	213	3	0	2.44×10^{-100}
mitogenome	352,526	352,735	chromosome 1	28,033,502	28,033,310	87.619	210	9	9	2.60×10^{-55}
mitogenome	157,677	157,885	chromosome 1	45,971,219	45,971,011	93.301	209	14	0	9.01×10^{-80}
mitogenome	460,504	460,711	chromosome 2	25,705,061	25,704,854	99.038	208	2	0	3.15×10^{-99}
mitogenome	405,086	405,293	chromosome 3	7,314,831	7,315,038	100	208	0	0	1.46×10^{-102}
mitogenome	337,859	338,065	chromosome 4	25,534,780	25,534,574	99.034	207	2	0	1.13×10^{-98}
mitogenome	135,498	135,698	chromosome 4	16,236,206	16,236,406	100	201	0	0	1.13×10^{-98}
mitogenome	90,749	90,947	chromosome 4	8,792,919	8,792,720	91.542	201	14	2	3.29×10^{-69}
mitogenome	421,589	421,788	chromosome 4	15,967,853	15,967,654	99.5	200	1	0	1.90×10^{-96}

mitogenome	159,592	159,790	chromosome 7	21,923,912	21,924,111	99.5	200	0	1	6.82×10^{-96}
mitogenome	90,749	90,947	chromosome 4	8,764,276	8,764,078	100	199	0	0	1.47×10^{-97}
mitogenome	456,829	457,027	chromosome 5	19,452,247	19,452,051	96.985	199	4	1	5.35×10^{-87}
mitogenome	242,343	242,533	chromosome 6	23,749,462	23,749,659	90.909	198	11	2	9.21×10^{-65}
mitogenome	345,332	345,522	chromosome 7	19,171,732	19,171,535	91.919	198	9	6	4.25×10^{-68}
mitogenome	381,352	381,538	chromosome 2	25,850,449	25,850,635	84.184	196	13	7	3.43×10^{-39}
mitogenome	282,432	282,626	chromosome 3	15,000,888	15,001,081	99.487	195	0	1	4.11×10^{-93}
mitogenome	381,352	381,545	chromosome 2	27,486,588	27,486,774	93.814	194	5	4	1.52×10^{-72}
mitogenome	275,844	276,035	chromosome 6	3,652,463	3,652,271	97.927	193	3	1	5.35×10^{-87}
mitogenome	176,535	176,726	chromosome 4	28,607,876	28,608,067	100	192	0	0	1.14×10^{-93}
mitogenome	340,982	341,172	chromosome 4	18,151,161	18,151,352	91.146	192	16	1	9.21×10^{-65}
mitogenome	84,218	84,409	chromosome 1	33,096,359	33,096,168	99.479	192	1	0	5.31×10^{-92}
mitogenome	101,877	102,061	chromosome 1	50,760,306	50,760,494	81.771	192	25	6	1.61×10^{-32}
mitogenome	101,877	102,061	chromosome 1	50,713,341	50,713,529	81.25	192	26	6	7.48×10^{-31}
mitogenome	318,037	318,226	chromosome 2	739,859	740,047	97.368	190	4	1	1.16×10^{-83}
mitogenome	83,867	84,055	chromosome 5	9,385,942	9,385,754	98.413	189	3	0	5.35×10^{-87}
mitogenome	257,101	257,288	chromosome 5	21,936,783	21,936,970	99.468	188	1	0	8.89×10^{-90}
mitogenome	277,219	277,405	chromosome 6	12,537,083	12,537,269	95.722	187	8	0	1.51×10^{-77}
mitogenome	502,243	502,429	chromosome 6	17,884,932	17,884,746	89.305	187	20	0	1.55×10^{-57}
mitogenome	374,924	375,109	chromosome 1	41,730,740	41,730,555	100	186	0	0	2.47×10^{-90}
mitogenome	353,299	353,483	chromosome 6	11,367,186	11,367,370	95.676	185	8	0	1.95×10^{-76}
mitogenome	440,586	440,769	chromosome 5	18,107,257	18,107,440	99.457	184	1	0	1.49×10^{-87}
mitogenome	479,744	479,926	chromosome 8	10,612,965	10,613,147	95.082	183	9	0	1.17×10^{-73}
mitogenome	65,183	65,365	chromosome 6	13,024,818	13,024,636	98.361	183	3	0	1.16×10^{-83}
mitogenome	123,314	123,496	chromosome 1	27,133,540	27,133,722	90.71	183	17	0	2.58×10^{-60}
mitogenome	237,857	238,039	chromosome 3	9,493,587	9,493,405	98.907	183	2	0	2.49×10^{-85}
mitogenome	341,327	341,504	chromosome 4	18,151,936	18,152,113	88.333	180	17	3	7.27×10^{-51}
mitogenome	297,088	297,252	chromosome 8	13,562,029	13,561,851	91.62	179	1	5	1.55×10^{-57}
mitogenome	201,846	202,023	chromosome 1	24,193,363	24,193,186	98.315	178	3	0	6.97×10^{-81}
mitogenome	358,615	358,792	chromosome 1	24,193,186	24,193,363	89.888	178	18	0	7.22×10^{-56}
mitogenome	379,845	380,021	chromosome 6	27,423,257	27,423,433	91.525	177	15	0	2.58×10^{-60}
mitogenome	302,111	302,286	chromosome 3	26,179,810	26,179,634	97.74	177	3	1	4.19×10^{-78}
mitogenome	120,144	120,320	chromosome 3	27,334,941	27,335,111	89.831	177	12	2	1.21×10^{-53}
mitogenome	294,536	294,710	chromosome 1	24,248,011	24,247,837	100	175	0	0	3.22×10^{-84}
mitogenome	64,984	65,158	chromosome 1	7,300,265	7,300,091	99.429	175	1	0	1.50×10^{-82}
mitogenome	294,536	294,710	chromosome 1	20,222,801	20,222,627	96	175	7	0	1.52×10^{-72}
mitogenome	64,984	65,158	chromosome 1	8,290,752	8,290,923	94.857	175	6	1	4.25×10^{-68}
mitogenome	268,017	268,190	chromosome 1	15,417,720	15,417,893	100	174	0	0	1.16×10^{-83}

mitogenome	378,379	378,550	chromosome 1	25,526,065	25,526,236	93.642	173	9	2	3.31×10^{-64}
mitogenome	378,379	378,550	chromosome 1	21,841,211	21,841,040	93.064	173	10	2	1.54×10^{-62}
mitogenome	220,457	220,628	chromosome 1	50,439,719	50,439,890	100	172	0	0	1.50×10^{-82}
mitogenome	150,819	150,988	chromosome 4	18,884,495	18,884,327	97.076	171	2	3	1.52×10^{-72}
mitogenome	156,849	157,019	chromosome 7	12,154,448	12,154,618	95.906	171	7	0	2.54×10^{-70}
mitogenome	409,795	409,965	chromosome 7	12,154,618	12,154,448	94.152	171	10	0	2.56×10^{-65}
mitogenome	71,043	71,210	chromosome 4	24,379,912	24,380,079	76.471	170	36	4	1.28×10^{-13}
mitogenome	460,764	460,926	chromosome 3	5,894,898	5,894,733	84.706	170	15	6	9.61×10^{-35}
mitogenome	237,658	237,826	chromosome 1	1,717,316	1,717,484	100	169	0	0	6.97×10^{-81}
mitogenome	150,819	150,987	chromosome 1	55,937,897	55,937,732	92.899	169	9	3	9.27×10^{-60}
mitogenome	229,091	229,259	chromosome 2	16,171,184	16,171,352	100	169	0	0	6.97×10^{-81}
mitogenome	46,751	46,918	chromosome 3	26,176,550	26,176,383	100	168	0	0	2.51×10^{-80}
mitogenome	272,982	273,148	chromosome 1	53,294,914	53,295,080	97.006	167	5	0	1.97×10^{-71}
mitogenome	118,618	118,783	chromosome 4	20,794,029	20,793,864	94.578	166	9	0	3.31×10^{-64}
mitogenome	453,323	453,488	chromosome 1	14,161,059	14,160,894	98.193	166	3	0	3.27×10^{-74}
mitogenome	181,397	181,562	chromosome 7	17,204,918	17,204,753	100	166	0	0	3.24×10^{-79}
mitogenome	181,397	181,562	chromosome 7	15,967,992	15,967,827	92.771	166	12	0	3.34×10^{-59}
mitogenome	354,723	354,887	chromosome 1	39,118,097	39,118,261	99.394	165	1	0	5.43×10^{-77}
mitogenome	333,058	333,221	chromosome 1	49,819,150	49,818,986	92.121	165	12	1	2.01×10^{-56}
mitogenome	118,618	118,780	chromosome 4	19,892,529	19,892,690	94.479	163	8	1	5.54×10^{-62}
mitogenome	55,967	56,129	chromosome 5	18,107,722	18,107,560	100	163	0	0	1.51×10^{-77}
mitogenome	298,537	298,698	chromosome 8	17,409,701	17,409,548	90.123	162	8	5	4.38×10^{-48}
mitogenome	109,633	109,793	chromosome 4	14,665,989	14,666,149	100	161	0	0	1.95×10^{-76}
mitogenome	190,304	190,463	chromosome 4	15,968,039	15,968,197	97.5	160	3	1	1.18×10^{-68}
mitogenome	435,801	435,960	chromosome 2	11,291,844	11,292,003	98.125	160	3	0	7.07×10^{-71}
mitogenome	162,946	163,105	chromosome 3	7,314,830	7,314,671	100	160	0	0	7.02×10^{-76}
mitogenome	378,930	379,085	chromosome 5	17,277,431	17,277,274	92.453	159	8	3	3.36×10^{-54}
mitogenome	197,801	197,958	chromosome 4	14,049,425	14,049,582	97.468	158	4	0	4.25×10^{-68}
mitogenome	106,832	106,987	chromosome 2	18,833,918	18,834,075	97.468	158	2	1	1.53×10^{-67}
mitogenome	106,832	106,987	chromosome 2	20,861,722	20,861,565	96.835	158	3	1	7.12×10^{-66}
mitogenome	335,225	335,381	chromosome 1	7,216,257	7,216,101	96.178	157	6	0	3.31×10^{-64}
mitogenome	335,225	335,381	chromosome 1	8,355,189	8,355,345	96.178	157	6	0	3.31×10^{-64}
mitogenome	320,791	320,946	chromosome 2	20,646,619	20,646,467	92.357	157	7	3	1.56×10^{-52}
mitogenome	198,826	198,981	chromosome 4	17,534,050	17,533,895	89.744	156	16	0	5.66×10^{-47}
mitogenome	356,242	356,397	chromosome 1	15,417,565	15,417,720	98.077	156	3	0	1.18×10^{-68}
mitogenome	76,623	76,777	chromosome 4	8,599,982	8,600,136	100	155	0	0	4.22×10^{-73}
mitogenome	150,834	150,988	chromosome 1	55,953,788	55,953,634	94.839	155	8	0	9.27×10^{-60}
mitogenome	320,791	320,942	chromosome 2	19,039,459	19,039,607	93.464	153	5	3	1.21×10^{-53}

mitogenome	68,692	68,842	chromosome 2	22,591,130	22,590,980	100	151	0	0	7.07×10^{-71}
mitogenome	444,343	444,490	chromosome 4	2,711,592	2,711,445	97.297	148	4	0	1.54×10^{-62}
mitogenome	444,343	444,490	chromosome 4	2,733,070	2,732,923	97.297	148	4	0	1.54×10^{-62}
mitogenome	338,939	339,086	chromosome 7	3,087,608	3,087,461	98.649	148	2	0	7.12×10^{-66}
mitogenome	319,569	319,716	chromosome 2	23,363,789	23,363,935	97.297	148	3	1	5.54×10^{-62}
mitogenome	319,569	319,716	chromosome 2	23,321,745	23,321,891	95.27	148	6	1	5.58×10^{-57}
mitogenome	285,415	285,561	chromosome 3	18,888,812	18,888,666	95.238	147	7	0	5.58×10^{-57}
mitogenome	222,984	223,129	chromosome 2	35,415,940	35,416,085	100	146	0	0	4.25×10^{-68}
mitogenome	71,977	72,120	chromosome 6	11,959,250	11,959,393	100	144	0	0	5.50×10^{-67}
mitogenome	102,464	102,605	chromosome 1	6,711,624	6,711,483	95.833	144	2	3	7.22×10^{-56}
mitogenome	86,131	86,274	chromosome 2	1,923,574	1,923,431	97.222	144	4	0	2.58×10^{-60}
mitogenome	86,131	86,274	chromosome 2	1,952,937	1,952,794	97.222	144	4	0	2.58×10^{-60}
mitogenome	103,659	103,801	chromosome 6	23,762,103	23,762,245	93.706	143	9	0	2.02×10^{-51}
mitogenome	179,145	179,286	chromosome 6	21,126,970	21,126,829	97.183	142	4	0	3.34×10^{-59}
mitogenome	179,145	179,286	chromosome 6	21,144,438	21,144,297	97.183	142	4	0	3.34×10^{-59}
mitogenome	179,145	179,286	chromosome 6	21,171,217	21,171,076	97.183	142	4	0	3.34×10^{-59}
mitogenome	179,145	179,286	chromosome 6	21,224,558	21,224,417	97.183	142	4	0	3.34×10^{-59}
mitogenome	179,145	179,286	chromosome 6	21,050,050	21,049,909	95.775	142	6	0	7.22×10^{-56}
mitogenome	85,295	85,436	chromosome 1	25,932,085	25,931,944	100	142	0	0	7.12×10^{-66}
mitogenome	196,367	196,507	chromosome 3	13,505,951	13,505,811	100	141	0	0	2.56×10^{-65}
mitogenome	120,336	120,476	chromosome 3	19,573,391	19,573,531	95.745	141	6	0	2.60×10^{-55}
mitogenome	64,843	64,981	chromosome 4	20,196,565	20,196,426	95	140	6	1	1.56×10^{-52}
mitogenome	64,843	64,981	chromosome 4	20,541,520	20,541,381	95	140	6	1	1.56×10^{-52}
mitogenome	353,079	353,218	chromosome 8	6,912,470	6,912,609	99.286	140	1	0	4.28×10^{-63}
mitogenome	353,079	353,218	chromosome 8	6,637,506	6,637,368	92.143	140	10	1	7.32×10^{-46}
mitogenome	228,383	228,522	chromosome 7	4,270,982	4,270,843	100	140	0	0	9.21×10^{-65}
mitogenome	118,576	118,715	chromosome 2	22,584,713	22,584,574	92.857	140	10	0	4.38×10^{-48}
mitogenome	357,372	357,510	chromosome 6	12,009,688	12,009,550	89.209	139	15	0	3.43×10^{-39}
mitogenome	130,773	130,911	chromosome 1	55,384,218	55,384,080	100	139	0	0	3.31×10^{-64}
mitogenome	418,164	418,302	chromosome 1	50,241,258	50,241,396	97.842	139	3	0	3.34×10^{-59}
mitogenome	418,164	418,302	chromosome 1	50,291,779	50,291,917	97.842	139	3	0	3.34×10^{-59}
mitogenome	418,164	418,302	chromosome 1	50,359,436	50,359,574	97.842	139	3	0	3.34×10^{-59}
mitogenome	418,164	418,302	chromosome 1	50,313,444	50,313,582	96.403	139	5	0	7.22×10^{-56}
mitogenome	380,229	380,367	chromosome 2	1,273,622	1,273,484	97.122	139	4	0	1.55×10^{-57}
mitogenome	338,941	339,077	chromosome 7	3,431,517	3,431,380	94.928	138	6	1	2.02×10^{-51}
mitogenome	340,826	340,963	chromosome 3	11,934,607	11,934,738	92.754	138	4	5	2.63×10^{-45}
mitogenome	355,863	355,999	chromosome 7	5,162,399	5,162,263	91.971	137	11	0	9.47×10^{-45}
mitogenome	104,220	104,355	chromosome 8	8,433,520	8,433,655	94.118	136	8	0	3.38×10^{-49}

mitogenome	102,944	103,079	chromosome 1	39,265,867	39,265,732	97.794	136	3	0	1.55×10^{-57}
mitogenome	418,164	418,299	chromosome 1	50,231,343	50,231,478	97.059	136	4	0	7.22×10^{-56}
mitogenome	418,164	418,299	chromosome 1	50,262,862	50,262,997	96.324	136	5	0	3.36×10^{-54}
mitogenome	163,661	163,789	chromosome 7	19,171,537	19,171,672	83.088	136	16	6	5.86×10^{-22}
mitogenome	340,829	340,963	chromosome 7	20,654,293	20,654,159	97.778	135	3	0	5.58×10^{-57}
mitogenome	83,350	83,484	chromosome 7	2,042,055	2,041,921	96.296	135	5	0	1.21×10^{-53}
mitogenome	41,439	41,573	chromosome 3	532,768	532,902	99.259	135	1	0	2.58×10^{-60}
mitogenome	418,164	418,297	chromosome 1	50,281,118	50,281,250	95.522	134	5	1	7.27×10^{-51}
mitogenome	153,069	153,202	chromosome 5	18,143,994	18,143,868	89.552	134	7	1	7.43×10^{-36}
mitogenome	153,069	153,202	chromosome 5	18,192,129	18,192,003	89.552	134	7	1	7.43×10^{-36}
mitogenome	223,340	223,472	chromosome 4	25,535,010	25,534,878	98.496	133	2	0	1.55×10^{-57}
mitogenome	118,576	118,708	chromosome 4	26,260,311	26,260,179	93.233	133	9	0	7.32×10^{-46}
mitogenome	118,576	118,708	chromosome 4	26,955,675	26,955,807	93.233	133	9	0	7.32×10^{-46}
mitogenome	145,455	145,583	chromosome 1	16,559,007	16,559,139	96.241	133	1	1	2.02×10^{-51}
mitogenome	392,677	392,809	chromosome 5	12,917,856	12,917,724	99.248	133	1	0	3.34×10^{-59}
mitogenome	356,081	356,213	chromosome 2	12,557,238	12,557,370	99.248	133	1	0	3.34×10^{-59}
mitogenome	356,081	356,213	chromosome 2	16,623,321	16,623,189	99.248	133	1	0	3.34×10^{-59}
mitogenome	164,677	164,808	chromosome 4	29,813,400	29,813,269	97.727	132	3	0	2.60×10^{-55}
mitogenome	411,921	412,052	chromosome 3	26,179,261	26,179,130	100	132	0	0	2.58×10^{-60}
mitogenome	345,297	345,427	chromosome 4	29,813,269	29,813,399	97.71	131	3	0	9.34×10^{-55}
mitogenome	332,367	332,497	chromosome 1	7,216,872	7,217,002	100	131	0	0	9.27×10^{-60}
mitogenome	332,367	332,497	chromosome 1	8,354,573	8,354,443	97.71	131	3	0	9.34×10^{-55}
mitogenome	145,457	145,583	chromosome 1	17,071,733	17,071,863	96.183	131	1	1	2.62×10^{-50}
mitogenome	2,389	2,519	chromosome 3	14,063,347	14,063,220	88.55	131	12	3	1.24×10^{-33}
mitogenome	133,537	133,666	chromosome 6	25,284,138	25,284,267	99.231	130	1	0	1.55×10^{-57}
mitogenome	133,537	133,666	chromosome 6	26,851,130	26,851,259	99.231	130	1	0	1.55×10^{-57}
mitogenome	223,062	223,189	chromosome 1	8,511,198	8,511,327	94.615	130	5	2	5.66×10^{-47}
mitogenome	223,062	223,189	chromosome 1	7,020,292	7,020,163	93.846	130	6	2	2.63×10^{-45}
mitogenome	298,810	298,939	chromosome 1	22,751,167	22,751,040	90.769	130	10	2	1.23×10^{-38}
mitogenome	298,810	298,939	chromosome 1	24,678,444	24,678,571	90.769	130	10	2	1.23×10^{-38}
mitogenome	439,605	439,734	chromosome 3	8,838,317	8,838,446	99.231	130	1	0	1.55×10^{-57}
mitogenome	381,121	381,249	chromosome 3	15,135,828	15,135,700	93.077	130	7	2	1.23×10^{-43}
mitogenome	61,983	62,110	chromosome 4	12,657,565	12,657,438	93.75	128	8	0	9.47×10^{-45}
mitogenome	317,969	318,096	chromosome 5	28,418,578	28,418,452	89.062	128	13	1	3.46×10^{-34}
mitogenome	129,164	129,290	chromosome 6	14,759,355	14,759,481	96.063	127	5	0	3.38×10^{-49}
mitogenome	129,164	129,290	chromosome 6	15,781,896	15,782,022	96.063	127	5	0	3.38×10^{-49}
mitogenome	195,060	195,186	chromosome 1	13,928,869	13,928,744	99.213	127	0	1	2.60×10^{-55}
mitogenome	71,421	71,546	chromosome 4	25,535,202	25,535,077	100	126	0	0	5.58×10^{-57}

mitogenome	479,007	479,130	chromosome 7	19,171,619	19,171,744	85.714	126	16	2	2.09×10^{-26}
mitogenome	258,023	258,147	chromosome 4	15,967,972	15,967,848	98.4	125	2	0	4.35×10^{-53}
mitogenome	158,372	158,496	chromosome 3	648,399	648,523	100	125	0	0	2.01×10^{-56}
mitogenome	157,076	157,199	chromosome 4	29,813,400	29,813,277	98.387	124	2	0	1.56×10^{-52}
mitogenome	386,959	387,082	chromosome 8	8,254,725	8,254,602	100	124	0	0	7.22×10^{-56}
mitogenome	284,889	285,012	chromosome 5	22,382,961	22,383,084	98.387	124	2	0	1.56×10^{-52}
mitogenome	1,724	1,846	chromosome 1	14,308,361	14,308,483	97.561	123	3	0	2.62×10^{-50}
mitogenome	70,115	70,237	chromosome 2	31,536,087	31,536,209	98.374	123	2	0	5.62×10^{-52}
mitogenome	435,565	435,683	chromosome 3	14,325,320	14,325,198	94.309	123	3	1	1.59×10^{-42}
mitogenome	299,071	299,192	chromosome 4	19,887,064	19,886,944	95.082	122	5	1	3.41×10^{-44}
mitogenome	299,071	299,192	chromosome 4	20,799,579	20,799,699	95.082	122	5	1	3.41×10^{-44}
mitogenome	163,070	163,191	chromosome 5	18,107,441	18,107,560	93.443	122	6	2	7.37×10^{-41}
mitogenome	67,626	67,746	chromosome 4	14,047,933	14,048,053	90.909	121	11	0	7.43×10^{-36}
mitogenome	466,473	466,593	chromosome 7	22,013,005	22,012,891	88.43	121	8	3	3.48×10^{-29}
mitogenome	345,990	346,110	chromosome 5	18,107,560	18,107,441	92.562	121	8	1	1.23×10^{-38}
mitogenome	71,212	71,332	chromosome 3	26,179,018	26,178,898	100	121	0	0	3.36×10^{-54}
mitogenome	113,993	114,112	chromosome 6	12,779,666	12,779,785	93.333	120	8	0	2.65×10^{-40}
mitogenome	53,483	53,602	chromosome 1	37,667,960	37,668,079	94.167	120	7	0	5.70×10^{-42}
mitogenome	352,159	352,278	chromosome 1	37,850,995	37,850,877	90	120	11	1	4.47×10^{-33}
mitogenome	373,477	373,596	chromosome 2	28,795,723	28,795,604	97.5	120	3	0	1.22×10^{-48}
mitogenome	112,573	112,691	chromosome 7	25,496,635	25,496,753	99.16	119	1	0	2.02×10^{-51}
mitogenome	54,605	54,723	chromosome 7	14,721,861	14,721,743	89.076	119	13	0	2.08×10^{-31}
mitogenome	257,910	258,028	chromosome 2	23,322,151	23,322,269	95.798	119	5	0	9.47×10^{-45}
mitogenome	3,153	3,269	chromosome 8	855,741	855,858	88.983	118	12	1	2.69×10^{-30}
mitogenome	355,319	355,436	chromosome 1	7,216,488	7,216,605	96.61	118	4	0	7.32×10^{-46}
mitogenome	355,319	355,436	chromosome 1	8,354,957	8,354,840	96.61	118	4	0	7.32×10^{-46}
mitogenome	117,504	117,621	chromosome 1	14,763,623	14,763,740	95.763	118	5	0	3.41×10^{-44}
mitogenome	456,563	456,680	chromosome 7	11,899,797	11,899,680	96.61	118	4	0	7.32×10^{-46}
mitogenome	179,896	180,013	chromosome 5	7,642,304	7,642,187	100	118	0	0	1.56×10^{-52}
mitogenome	179,896	180,013	chromosome 5	6,948,006	6,947,889	97.458	118	3	0	1.57×10^{-47}
mitogenome	239,966	240,083	chromosome 2	14,377,547	14,377,664	98.305	118	2	0	3.38×10^{-49}
mitogenome	332,043	332,156	chromosome 3	29,209,726	29,209,609	90.678	118	7	2	4.47×10^{-33}
mitogenome	8,494	8,609	chromosome 2	2,196,500	2,196,385	99.138	116	1	0	9.41×10^{-50}
mitogenome	98,946	99,061	chromosome 2	21,448,197	21,448,312	94.828	116	6	0	2.05×10^{-41}
mitogenome	2,963	3,078	chromosome 3	33,802,431	33,802,546	100	116	0	0	2.02×10^{-51}
mitogenome	2,963	3,078	chromosome 3	34,040,096	34,039,981	97.414	116	3	0	2.04×10^{-46}
mitogenome	272,317	272,432	chromosome 3	15,436,350	15,436,465	93.966	116	7	0	9.54×10^{-40}
mitogenome	122,160	122,273	chromosome 3	27,497,738	27,497,623	93.966	116	5	1	3.43×10^{-39}

mitogenome	193,594	193,709	chromosome 3	2,455,315	2,455,429	93.103	116	7	1	1.60×10^{-37}
mitogenome	259,406	259,520	chromosome 4	11,497,321	11,497,435	100	115	0	0	7.27×10^{-51}
mitogenome	274,889	275,003	chromosome 4	30,693,734	30,693,621	90.435	115	10	1	5.78×10^{-32}
mitogenome	389,776	389,890	chromosome 1	27,188,859	27,188,745	92.174	115	9	0	7.43×10^{-36}
mitogenome	278,148	278,260	chromosome 7	15,439,300	15,439,414	90.435	115	9	1	5.78×10^{-32}
mitogenome	257,911	258,025	chromosome 2	23,364,182	23,364,296	98.261	115	2	0	1.57×10^{-47}
mitogenome	340,417	340,525	chromosome 2	2,676,785	2,676,673	87.826	115	6	4	2.71×10^{-25}
mitogenome	259,113	259,227	chromosome 3	26,179,018	26,179,132	99.13	115	1	0	3.38×10^{-49}
mitogenome	394,593	394,706	chromosome 1	8,354,709	8,354,596	99.123	114	1	0	1.22×10^{-48}
mitogenome	394,593	394,706	chromosome 1	7,216,736	7,216,849	97.368	114	3	0	2.63×10^{-45}
mitogenome	278,148	278,260	chromosome 7	15,422,174	15,422,287	91.228	114	9	1	4.47×10^{-33}
mitogenome	340,417	340,525	chromosome 2	3,198,156	3,198,045	88.596	114	6	3	2.09×10^{-26}
mitogenome	300,669	300,781	chromosome 6	22,199,686	22,199,574	92.92	113	8	0	2.06×10^{-36}
mitogenome	85,466	85,578	chromosome 1	33,683,201	33,683,313	94.69	113	6	0	9.54×10^{-40}
mitogenome	352,205	352,317	chromosome 1	46,320,154	46,320,265	92.92	113	7	1	7.43×10^{-36}
mitogenome	396,102	396,214	chromosome 3	30,778,201	30,778,313	99.115	113	1	0	4.38×10^{-48}
mitogenome	102,316	102,427	chromosome 8	9,865,918	9,865,807	99.107	112	1	0	1.57×10^{-47}
mitogenome	67,355	67,466	chromosome 7	13,117,469	13,117,580	96.429	112	4	0	1.59×10^{-42}
mitogenome	325,357	325,468	chromosome 3	7,670,670	7,670,781	95.536	112	5	0	7.37×10^{-41}
mitogenome	338,918	339,027	chromosome 3	13,560,818	13,560,707	95.536	112	3	1	2.65×10^{-40}
mitogenome	325,357	325,468	chromosome 3	999,187	999,077	94.643	112	5	1	1.23×10^{-38}
mitogenome	221,663	221,773	chromosome 4	15,177,260	15,177,150	100	111	0	0	1.22×10^{-48}
mitogenome	221,663	221,773	chromosome 4	15,256,624	15,256,734	95.495	111	5	0	2.65×10^{-40}
mitogenome	149,025	149,135	chromosome 3	34,299,981	34,299,872	95.495	111	4	1	9.54×10^{-40}
mitogenome	156,342	156,451	chromosome 6	8,053,192	8,053,083	95.455	110	5	0	9.54×10^{-40}
mitogenome	156,342	156,451	chromosome 6	9,259,871	9,259,762	95.455	110	5	0	9.54×10^{-40}
mitogenome	495,119	495,228	chromosome 1	55,460,397	55,460,506	99.091	110	1	0	2.04×10^{-46}
mitogenome	387,944	388,053	chromosome 1	46,877,788	46,877,897	95.455	110	5	0	9.54×10^{-40}
mitogenome	361,623	361,731	chromosome 4	17,556,845	17,556,953	100	109	0	0	1.57×10^{-47}
mitogenome	501,476	501,584	chromosome 4	17,556,845	17,556,953	100	109	0	0	1.57×10^{-47}
mitogenome	437,772	437,880	chromosome 1	7,216,005	7,215,897	96.33	109	4	0	7.37×10^{-41}
mitogenome	317,772	317,880	chromosome 1	41,910,735	41,910,843	96.33	109	4	0	7.37×10^{-41}
mitogenome	185,466	185,574	chromosome 7	25,496,452	25,496,560	100	109	0	0	1.57×10^{-47}
mitogenome	146,542	146,650	chromosome 2	16,913,271	16,913,379	96.33	109	4	0	7.37×10^{-41}
mitogenome	86,943	87,050	chromosome 1	4,265,100	4,265,206	96.296	108	3	1	9.54×10^{-40}
mitogenome	86,943	87,050	chromosome 1	6,076,977	6,077,083	96.296	108	3	1	9.54×10^{-40}
mitogenome	315,789	315,896	chromosome 1	8,354,956	8,355,063	94.444	108	6	0	5.74×10^{-37}
mitogenome	315,789	315,896	chromosome 1	7,216,489	7,216,382	90.741	108	10	0	2.69×10^{-30}

mitogenome	242,757	242,864	chromosome 5	13,795,330	13,795,223	96.296	108	4	0	2.65×10^{-40}
mitogenome	242,757	242,864	chromosome 5	14,898,805	14,898,698	95.37	108	5	0	1.23×10^{-38}
mitogenome	333,003	333,107	chromosome 2	29,146,426	29,146,532	89.815	108	7	3	1.62×10^{-27}
mitogenome	319,460	319,567	chromosome 3	27,708,776	27,708,676	92.593	108	1	1	2.08×10^{-31}
mitogenome	367,811	367,917	chromosome 4	29,815,327	29,815,433	100	107	0	0	2.04×10^{-46}
mitogenome	114,048	114,152	chromosome 4	29,813,162	29,813,268	97.196	107	1	1	7.37×10^{-41}
mitogenome	250,772	250,878	chromosome 6	11,959,498	11,959,392	100	107	0	0	2.04×10^{-46}
mitogenome	333,130	333,233	chromosome 6	17,917,436	17,917,334	89.72	107	4	6	7.53×10^{-26}
mitogenome	385,245	385,351	chromosome 5	17,955,184	17,955,078	99.065	107	1	0	9.47×10^{-45}
mitogenome	141,452	141,558	chromosome 3	748,238	748,132	95.327	107	5	0	4.44×10^{-38}
mitogenome	117,175	117,280	chromosome 4	16,644,934	16,645,039	98.113	106	2	0	1.59×10^{-42}
mitogenome	336,360	336,465	chromosome 1	8,355,452	8,355,347	98.113	106	2	0	1.59×10^{-42}
mitogenome	495,584	495,687	chromosome 7	19,171,635	19,171,740	92.453	106	6	2	5.78×10^{-32}
mitogenome	388,192	388,296	chromosome 6	15,470,956	15,470,852	97.143	105	3	0	2.65×10^{-40}
mitogenome	388,192	388,296	chromosome 6	14,482,753	14,482,649	96.19	105	4	0	1.23×10^{-38}
mitogenome	424,912	425,014	chromosome 6	26,554,192	26,554,088	92.381	105	6	1	2.08×10^{-31}
mitogenome	437,773	437,877	chromosome 1	8,355,442	8,355,546	99.048	105	1	0	1.23×10^{-43}
mitogenome	308,838	308,942	chromosome 5	14,898,908	14,898,804	95.238	105	5	0	5.74×10^{-37}
mitogenome	67,434	67,535	chromosome 5	13,216,404	13,216,300	94.286	105	3	1	3.46×10^{-34}
mitogenome	304,768	304,872	chromosome 3	17,724,799	17,724,697	93.333	105	5	1	4.47×10^{-33}
mitogenome	199,014	199,118	chromosome 3	17,724,799	17,724,697	93.333	105	5	1	4.47×10^{-33}
mitogenome	82,776	82,879	chromosome 1	33,096,566	33,096,669	96.154	104	4	0	4.44×10^{-38}
mitogenome	7,268	7,371	chromosome 7	8,516,628	8,516,731	98.077	104	2	0	2.05×10^{-41}
mitogenome	223,134	223,237	chromosome 5	999,217	999,320	98.077	104	2	0	2.05×10^{-41}
mitogenome	223,134	223,237	chromosome 5	1,037,889	1,037,992	98.077	104	2	0	2.05×10^{-41}
mitogenome	223,134	223,237	chromosome 5	934,537	934,640	97.115	104	3	0	9.54×10^{-40}
mitogenome	223,134	223,237	chromosome 5	1,161,495	1,161,598	97.115	104	3	0	9.54×10^{-40}
mitogenome	223,134	223,237	chromosome 5	1,200,360	1,200,463	96.154	104	4	0	4.44×10^{-38}
mitogenome	62,687	62,790	chromosome 3	13,914,085	13,913,982	97.115	104	3	0	9.54×10^{-40}
mitogenome	144,524	144,626	chromosome 4	12,652,206	12,652,305	89.32	103	8	2	9.74×10^{-25}
mitogenome	386,932	387,033	chromosome 6	5,567,797	5,567,899	96.117	103	3	1	5.74×10^{-37}
mitogenome	424,912	425,012	chromosome 6	24,952,850	24,952,748	94.175	103	4	1	1.24×10^{-33}
mitogenome	424,912	425,012	chromosome 6	26,559,647	26,559,545	94.175	103	4	1	1.24×10^{-33}
mitogenome	66,783	66,884	chromosome 6	22,718,615	22,718,717	90.291	103	9	1	5.82×10^{-27}
mitogenome	504,746	504,848	chromosome 1	44,344,743	44,344,845	99.029	103	1	0	1.59×10^{-42}
mitogenome	332,147	332,249	chromosome 1	40,194,609	40,194,711	94.175	103	6	0	3.46×10^{-34}
mitogenome	439,488	439,590	chromosome 2	7,402,817	7,402,919	98.058	103	2	0	7.37×10^{-41}
mitogenome	73,073	73,175	chromosome 3	10,337,315	10,337,213	99.029	103	1	0	1.59×10^{-42}

mitogenome	99,285	99,386	chromosome 4	3,270,557	3,270,658	96.078	102	4	0	5.74×10^{-37}
mitogenome	155,801	155,902	chromosome 8	2,755,093	2,755,193	94.118	102	5	1	4.47×10^{-33}
mitogenome	332,973	333,074	chromosome 1	9,808,729	9,808,628	100	102	0	0	1.23×10^{-43}
mitogenome	302,649	302,748	chromosome 1	13,960,238	13,960,339	95.098	102	3	2	9.61×10^{-35}
mitogenome	286,097	286,198	chromosome 1	46,468,766	46,468,665	94.118	102	6	0	1.24×10^{-33}
mitogenome	161,802	161,903	chromosome 7	23,303,637	23,303,738	98.039	102	2	0	2.65×10^{-40}
mitogenome	427,897	427,998	chromosome 7	2,641,062	2,640,961	97.059	102	3	0	1.23×10^{-38}
mitogenome	330,965	331,066	chromosome 5	19,246,221	19,246,322	99.02	102	1	0	5.70×10^{-42}
mitogenome	223,134	223,235	chromosome 5	1,592,060	1,591,959	97.059	102	3	0	1.23×10^{-38}
mitogenome	343,532	343,633	chromosome 3	26,179,634	26,179,533	99.02	102	1	0	5.70×10^{-42}
mitogenome	308,838	308,939	chromosome 3	16,392,143	16,392,238	89.216	102	5	2	1.26×10^{-23}
mitogenome	189,870	189,970	chromosome 8	17,757,701	17,757,601	99.01	101	1	0	2.05×10^{-41}
mitogenome	136,868	136,968	chromosome 6	17,819,576	17,819,476	97.03	101	3	0	4.44×10^{-38}
mitogenome	302,649	302,748	chromosome 1	13,917,557	13,917,657	94.059	101	5	1	1.61×10^{-32}
mitogenome	287,603	287,703	chromosome 7	23,131,679	23,131,768	85.149	101	4	4	9.88×10^{-15}
mitogenome	223,134	223,234	chromosome 5	945,671	945,771	97.03	101	3	0	4.44×10^{-38}
mitogenome	196,110	196,209	chromosome 6	24,947,282	24,947,183	94	100	6	0	1.61×10^{-32}
mitogenome	171,405	171,504	chromosome 3	2,455,220	2,455,319	98	100	2	0	3.43×10^{-39}
mitogenome	407,168	407,266	chromosome 4	17,554,761	17,554,859	100	99	0	0	5.70×10^{-42}
mitogenome	196,111	196,209	chromosome 6	26,554,088	26,553,990	93.939	99	6	0	5.78×10^{-32}
mitogenome	394,134	394,232	chromosome 1	10,760,939	10,760,841	96.97	99	3	0	5.74×10^{-37}
mitogenome	306,651	306,749	chromosome 1	17,322,932	17,322,834	94.949	99	5	0	1.24×10^{-33}
mitogenome	317,206	317,304	chromosome 1	2,919,490	2,919,586	94.949	99	3	1	4.47×10^{-33}
mitogenome	394,134	394,232	chromosome 1	12,254,509	12,254,411	91.919	99	8	0	1.25×10^{-28}
mitogenome	130,402	130,497	chromosome 1	17,071,862	17,071,960	91.919	99	5	1	1.62×10^{-27}
mitogenome	394,134	394,232	chromosome 1	11,756,388	11,756,290	90.909	99	9	0	5.82×10^{-27}
mitogenome	317,068	317,165	chromosome 2	20,104,115	20,104,209	83.838	99	11	5	1.28×10^{-13}
mitogenome	317,068	317,165	chromosome 2	20,137,335	20,137,429	81.818	99	13	5	2.77×10^{-10}
mitogenome	60,751	60,848	chromosome 4	20,541,618	20,541,521	97.959	98	2	0	4.44×10^{-38}
mitogenome	60,751	60,848	chromosome 4	20,196,663	20,196,566	96.939	98	3	0	2.06×10^{-36}
mitogenome	440,936	441,033	chromosome 6	19,748,634	19,748,730	95.918	98	3	1	3.46×10^{-34}
mitogenome	336,368	336,465	chromosome 1	7,216,002	7,216,099	98.98	98	1	0	9.54×10^{-40}
mitogenome	136,094	136,190	chromosome 1	39,118,264	39,118,361	95.918	98	3	1	3.46×10^{-34}
mitogenome	61,258	61,354	chromosome 7	13,240,926	13,241,021	95.918	98	1	2	1.24×10^{-33}
mitogenome	164,678	164,773	chromosome 7	19,171,635	19,171,732	91.837	98	6	2	1.62×10^{-27}
mitogenome	157,077	157,172	chromosome 7	19,171,635	19,171,732	90.816	98	7	2	7.53×10^{-26}
mitogenome	317,073	317,168	chromosome 7	19,171,635	19,171,732	88.776	98	9	2	1.63×10^{-22}
mitogenome	171,407	171,504	chromosome 3	2,454,223	2,454,127	95.918	98	3	1	3.46×10^{-34}

mitogenome	495,583	495,679	chromosome 4	29,813,400	29,813,304	96.907	97	3	0	7.43×10^{-36}
mitogenome	117,061	117,157	chromosome 6	12,089,280	12,089,184	98.969	97	1	0	3.43×10^{-39}
mitogenome	285,155	285,251	chromosome 6	27,046,997	27,047,093	91.753	97	8	0	1.62×10^{-27}
mitogenome	122,179	122,273	chromosome 6	22,558,436	22,558,340	91.753	97	6	1	5.82×10^{-27}
mitogenome	114,054	114,150	chromosome 1	2,919,586	2,919,490	97.938	97	2	0	1.60×10^{-37}
mitogenome	190,153	190,249	chromosome 1	52,476,662	52,476,566	95.876	97	4	0	3.46×10^{-34}
mitogenome	227,155	227,251	chromosome 2	35,416,086	35,416,182	97.938	97	2	0	1.60×10^{-37}
mitogenome	114,068	114,164	chromosome 2	1,751,658	1,751,562	94.845	97	5	0	1.61×10^{-32}
mitogenome	117,369	117,465	chromosome 2	12,239,794	12,239,699	88.66	97	10	1	5.86×10^{-22}
mitogenome	117,369	117,465	chromosome 2	16,919,769	16,919,864	88.66	97	10	1	5.86×10^{-22}
mitogenome	377,065	377,160	chromosome 6	7,057,714	7,057,619	100	96	0	0	2.65×10^{-40}
mitogenome	130,402	130,497	chromosome 1	16,559,138	16,559,233	94.792	96	5	0	5.78×10^{-32}
mitogenome	438,737	438,831	chromosome 1	56,168,560	56,168,654	92.708	96	5	2	4.50×10^{-28}
mitogenome	236,255	236,350	chromosome 7	8,516,396	8,516,491	98.958	96	1	0	1.23×10^{-38}
mitogenome	144,557	144,652	chromosome 7	6,979,993	6,980,080	89.583	96	2	1	2.11×10^{-21}
mitogenome	428,008	428,103	chromosome 5	11,734,462	11,734,557	98.958	96	1	0	1.23×10^{-38}
mitogenome	453,710	453,805	chromosome 2	14,377,801	14,377,896	100	96	0	0	2.65×10^{-40}
mitogenome	391,155	391,249	chromosome 8	8,255,241	8,255,147	97.895	95	2	0	2.06×10^{-36}
mitogenome	440,939	441,033	chromosome 6	18,912,900	18,912,807	96.842	95	2	1	3.46×10^{-34}
mitogenome	223,676	223,770	chromosome 1	46,860,632	46,860,726	100	95	0	0	9.54×10^{-40}
mitogenome	117,277	117,371	chromosome 7	14,721,650	14,721,744	96.842	95	3	0	9.61×10^{-35}
mitogenome	138,259	138,350	chromosome 7	1,242,229	1,242,323	94.737	95	2	2	2.69×10^{-30}
mitogenome	96,799	96,893	chromosome 5	14,562,889	14,562,795	100	95	0	0	9.54×10^{-40}
mitogenome	151,511	151,604	chromosome 5	16,289,719	16,289,626	94.737	95	3	2	7.48×10^{-31}
mitogenome	455,671	455,765	chromosome 2	740,133	740,039	98.947	95	1	0	4.44×10^{-38}
mitogenome	297,021	297,115	chromosome 2	16,834,726	16,834,634	95.789	95	2	1	1.61×10^{-32}
mitogenome	297,021	297,115	chromosome 2	12,317,899	12,317,993	94.737	95	5	0	2.08×10^{-31}
mitogenome	156,208	156,302	chromosome 2	15,436,144	15,436,050	93.684	95	6	0	9.68×10^{-30}
mitogenome	503,534	503,626	chromosome 1	896,379	896,472	94.681	94	4	1	2.69×10^{-30}
mitogenome	108,071	108,160	chromosome 1	18,653,687	18,653,594	90.426	94	5	2	4.53×10^{-23}
mitogenome	332,527	332,620	chromosome 7	22,792,948	22,793,041	96.809	94	3	0	3.46×10^{-34}
mitogenome	287,280	287,373	chromosome 7	17,165,009	17,165,100	94.681	94	3	1	2.69×10^{-30}
mitogenome	5,314	5,407	chromosome 5	18,107,045	18,107,138	93.617	94	6	0	3.48×10^{-29}
mitogenome	297,050	297,143	chromosome 3	13,560,819	13,560,726	97.872	94	2	0	7.43×10^{-36}
mitogenome	287,280	287,373	chromosome 3	5,871,052	5,870,959	96.809	94	3	0	3.46×10^{-34}
mitogenome	7,555	7,647	chromosome 4	28,212,983	28,212,891	98.925	93	1	0	5.74×10^{-37}
mitogenome	465,282	465,374	chromosome 1	32,993,124	32,993,032	100	93	0	0	1.23×10^{-38}
mitogenome	120,317	120,409	chromosome 7	12,982,325	12,982,417	96.774	93	3	0	1.24×10^{-33}

mitogenome	187,880	187,972	chromosome 5	10,151,498	10,151,406	94.624	93	5	0	2.69×10^{-30}
mitogenome	318,241	318,333	chromosome 2	739,767	739,859	97.849	93	2	0	2.67×10^{-35}
mitogenome	237,787	237,879	chromosome 2	33,050,542	33,050,450	92.473	93	7	0	5.82×10^{-27}
mitogenome	237,787	237,879	chromosome 2	36,209,281	36,209,373	92.473	93	7	0	5.82×10^{-27}
mitogenome	336,685	336,777	chromosome 3	29,986,601	29,986,509	100	93	0	0	1.23×10^{-38}
mitogenome	438,440	438,531	chromosome 4	4,095,122	4,095,031	98.913	92	1	0	2.06×10^{-36}
mitogenome	272,317	272,408	chromosome 6	5,422,907	5,422,998	95.652	92	4	0	2.08×10^{-31}
mitogenome	285,160	285,251	chromosome 6	25,476,931	25,477,022	95.652	92	4	0	2.08×10^{-31}
mitogenome	280,209	280,300	chromosome 1	25,840,738	25,840,647	98.913	92	1	0	2.06×10^{-36}
mitogenome	461,196	461,281	chromosome 1	9,420,369	9,420,460	90.217	92	3	1	2.11×10^{-21}
mitogenome	435,684	435,775	chromosome 7	25,454,271	25,454,362	93.478	92	6	0	4.50×10^{-28}
mitogenome	374,712	374,802	chromosome 8	17,757,878	17,757,788	100	91	0	0	1.60×10^{-37}
mitogenome	439,346	439,436	chromosome 7	8,516,627	8,516,537	97.802	91	2	0	3.46×10^{-34}
mitogenome	157,397	157,486	chromosome 5	22,456,109	22,456,199	91.209	91	7	1	1.26×10^{-23}
mitogenome	331,714	331,804	chromosome 2	6,228,781	6,228,691	95.604	91	4	0	7.48×10^{-31}
mitogenome	54,017	54,106	chromosome 2	71,647	71,559	92.308	91	4	2	9.74×10^{-25}
mitogenome	54,017	54,106	chromosome 2	33,138,232	33,138,144	92.308	91	4	2	9.74×10^{-25}
mitogenome	274,012	274,101	chromosome 3	15,978,403	15,978,313	97.802	91	1	1	1.24×10^{-33}
mitogenome	479,029	479,118	chromosome 4	29,813,393	29,813,304	92.222	90	7	0	2.71×10^{-25}
mitogenome	396,168	396,257	chromosome 8	8,236,879	8,236,790	95.556	90	4	0	2.69×10^{-30}
mitogenome	343,301	343,390	chromosome 8	8,254,440	8,254,529	95.556	90	4	0	2.69×10^{-30}
mitogenome	424,921	425,010	chromosome 6	24,947,374	24,947,285	95.556	90	4	0	2.69×10^{-30}
mitogenome	276,486	276,575	chromosome 1	38,694,940	38,694,851	98.889	90	1	0	2.67×10^{-35}
mitogenome	390,308	390,397	chromosome 1	18,605,961	18,606,050	94.444	90	5	0	1.25×10^{-28}
mitogenome	278,908	278,997	chromosome 1	35,973,670	35,973,757	94.444	90	3	2	4.50×10^{-28}
mitogenome	418,878	418,967	chromosome 7	5,162,805	5,162,894	97.778	90	2	0	1.24×10^{-33}
mitogenome	122,390	122,479	chromosome 2	23,364,183	23,364,094	98.889	90	1	0	2.67×10^{-35}
mitogenome	331,714	331,803	chromosome 2	5,000,033	4,999,944	95.556	90	4	0	2.69×10^{-30}
mitogenome	64,733	64,821	chromosome 8	6,759,204	6,759,116	94.382	89	5	0	4.50×10^{-28}
mitogenome	448,765	448,853	chromosome 6	16,005,572	16,005,660	92.135	89	7	0	9.74×10^{-25}
mitogenome	448,765	448,853	chromosome 6	15,005,447	15,005,535	91.011	89	8	0	4.53×10^{-23}
mitogenome	82,809	82,897	chromosome 1	1,443,354	1,443,442	97.753	89	2	0	4.47×10^{-33}
mitogenome	157,463	157,551	chromosome 1	54,559,694	54,559,782	95.506	89	4	0	9.68×10^{-30}
mitogenome	394,107	394,195	chromosome 5	3,422,326	3,422,238	96.629	89	3	0	2.08×10^{-31}
mitogenome	441,300	441,388	chromosome 3	16,493,657	16,493,569	95.506	89	4	0	9.68×10^{-30}
mitogenome	180,083	180,171	chromosome 3	30,082,396	30,082,308	93.258	89	6	0	2.09×10^{-26}
mitogenome	18,109	18,196	chromosome 8	17,758,022	17,757,935	100	88	0	0	7.43×10^{-36}
mitogenome	171,787	171,874	chromosome 7	21,482,977	21,482,890	100	88	0	0	7.43×10^{-36}

mitogenome	200,825	200,912	chromosome 7	26,029,966	26,029,879	96.591	88	3	0	7.48×10^{-31}
mitogenome	419,401	419,488	chromosome 7	26,029,879	26,029,966	96.591	88	3	0	7.48×10^{-31}
mitogenome	382,715	382,802	chromosome 5	13,795,695	13,795,608	97.727	88	2	0	1.61×10^{-32}
mitogenome	185,107	185,194	chromosome 5	18,108,012	18,107,925	97.727	88	2	0	1.61×10^{-32}
mitogenome	103,808	103,895	chromosome 2	18,833,914	18,833,827	97.727	88	2	0	1.61×10^{-32}
mitogenome	103,808	103,895	chromosome 2	20,861,726	20,861,813	97.727	88	2	0	1.61×10^{-32}
mitogenome	419,666	419,753	chromosome 3	2,508,553	2,508,466	97.727	88	2	0	1.61×10^{-32}
mitogenome	4,804	4,890	chromosome 4	14,199,158	14,199,072	95.402	87	4	0	1.25×10^{-28}
mitogenome	468,263	468,349	chromosome 4	10,430,061	10,430,146	94.253	87	4	1	2.09×10^{-26}
mitogenome	468,263	468,349	chromosome 6	2,338,303	2,338,388	95.402	87	3	1	4.50×10^{-28}
mitogenome	301,795	301,881	chromosome 1	13,230,390	13,230,476	95.402	87	4	0	1.25×10^{-28}
mitogenome	468,263	468,349	chromosome 1	56,047,118	56,047,203	93.103	87	5	1	9.74×10^{-25}
mitogenome	279,862	279,947	chromosome 7	6,122,862	6,122,948	96.552	87	2	1	9.68×10^{-30}
mitogenome	468,263	468,349	chromosome 7	11,913,378	11,913,463	95.402	87	3	1	4.50×10^{-28}
mitogenome	468,263	468,349	chromosome 7	13,042,501	13,042,416	95.402	87	3	1	4.50×10^{-28}
mitogenome	252,010	252,096	chromosome 2	1,195,453	1,195,539	100	87	0	0	2.67×10^{-35}
mitogenome	122,393	122,479	chromosome 2	23,322,150	23,322,064	100	87	0	0	2.67×10^{-35}
mitogenome	143,095	143,181	chromosome 2	8,550,917	8,550,831	94.253	87	5	0	5.82×10^{-27}
mitogenome	468,263	468,349	chromosome 2	2,687,009	2,686,924	93.103	87	5	1	9.74×10^{-25}
mitogenome	2,628	2,714	chromosome 3	27,262,760	27,262,846	98.851	87	1	0	1.24×10^{-33}
mitogenome	180,083	180,169	chromosome 3	30,187,840	30,187,754	97.701	87	2	0	5.78×10^{-32}
mitogenome	258,663	258,748	chromosome 4	22,441,638	22,441,553	97.674	86	2	0	2.08×10^{-31}
mitogenome	36,578	36,663	chromosome 6	27,575,550	27,575,465	100	86	0	0	9.61×10^{-35}
mitogenome	36,578	36,663	chromosome 6	27,593,475	27,593,390	100	86	0	0	9.61×10^{-35}
mitogenome	331,587	331,672	chromosome 1	56,301,733	56,301,648	100	86	0	0	9.61×10^{-35}
mitogenome	279,862	279,947	chromosome 7	6,016,326	6,016,411	97.674	86	2	0	2.08×10^{-31}
mitogenome	279,862	279,947	chromosome 7	6,160,471	6,160,556	97.674	86	2	0	2.08×10^{-31}
mitogenome	456,563	456,647	chromosome 7	11,916,997	11,916,912	96.512	86	2	1	3.48×10^{-29}
mitogenome	438,904	438,989	chromosome 7	9,431,248	9,431,333	95.349	86	4	0	4.50×10^{-28}
mitogenome	438,904	438,989	chromosome 7	10,023,461	10,023,376	95.349	86	4	0	4.50×10^{-28}
mitogenome	64,733	64,818	chromosome 7	6,211,629	6,211,710	90.698	86	4	1	2.73×10^{-20}
mitogenome	449,762	449,847	chromosome 5	18,144,079	18,143,994	97.674	86	2	0	2.08×10^{-31}
mitogenome	449,762	449,847	chromosome 5	18,192,214	18,192,129	95.349	86	4	0	4.50×10^{-28}
mitogenome	390,114	390,199	chromosome 2	13,629,654	13,629,569	95.349	86	4	0	4.50×10^{-28}
mitogenome	503,390	503,475	chromosome 3	9,063,672	9,063,757	97.674	86	2	0	2.08×10^{-31}
mitogenome	121,608	121,692	chromosome 6	20,056,963	20,057,047	96.471	85	3	0	3.48×10^{-29}
mitogenome	121,608	121,692	chromosome 6	18,521,993	18,521,910	95.294	85	3	1	5.82×10^{-27}
mitogenome	64,733	64,817	chromosome 1	49,224,512	49,224,428	96.471	85	3	0	3.48×10^{-29}

mitogenome	101,497	101,580	chromosome 4	8,764,276	8,764,359	96.429	84	3	0	1.25×10^{-28}
mitogenome	301,344	301,427	chromosome 8	14,052,213	14,052,296	95.238	84	4	0	5.82×10^{-27}
mitogenome	430,384	430,467	chromosome 6	26,559,809	26,559,892	95.238	84	4	0	5.82×10^{-27}
mitogenome	430,384	430,467	chromosome 6	24,953,012	24,953,094	94.048	84	4	1	9.74×10^{-25}
mitogenome	124,407	124,490	chromosome 7	7,054,717	7,054,796	91.667	84	3	2	7.59×10^{-21}
mitogenome	222,597	222,679	chromosome 4	18,021,980	18,022,062	96.386	83	3	0	4.50×10^{-28}
mitogenome	444,838	444,920	chromosome 8	6,844,465	6,844,383	95.181	83	4	0	2.09×10^{-26}
mitogenome	85,078	85,160	chromosome 1	27,028,514	27,028,432	96.386	83	3	0	4.50×10^{-28}
mitogenome	356,075	356,157	chromosome 1	23,023,028	23,022,946	90.361	83	8	0	9.81×10^{-20}
mitogenome	127,499	127,579	chromosome 1	25,579,301	25,579,223	87.952	83	4	6	9.88×10^{-15}
mitogenome	447,536	447,618	chromosome 7	8,516,734	8,516,816	98.795	83	1	0	2.08×10^{-31}
mitogenome	350,102	350,184	chromosome 5	5,356,239	5,356,157	96.386	83	3	0	4.50×10^{-28}
mitogenome	350,102	350,184	chromosome 5	6,840,615	6,840,697	96.386	83	3	0	4.50×10^{-28}
mitogenome	376,270	376,352	chromosome 5	2,877,303	2,877,383	93.976	83	3	1	3.50×10^{-24}
mitogenome	156,105	156,182	chromosome 2	15,436,221	15,436,139	90.361	83	3	1	4.57×10^{-18}
mitogenome	350,102	350,184	chromosome 3	15,632,708	15,632,626	95.181	83	4	0	2.09×10^{-26}
mitogenome	407,336	407,417	chromosome 4	17,554,860	17,554,941	100	82	0	0	1.61×10^{-32}
mitogenome	424,825	424,905	chromosome 6	24,952,921	24,953,001	93.902	82	3	2	1.26×10^{-23}
mitogenome	424,825	424,905	chromosome 6	26,559,718	26,559,798	93.902	82	3	2	1.26×10^{-23}
mitogenome	157,151	157,232	chromosome 1	42,012,299	42,012,218	100	82	0	0	1.61×10^{-32}
mitogenome	157,151	157,232	chromosome 1	43,312,643	43,312,724	100	82	0	0	1.61×10^{-32}
mitogenome	272,762	272,841	chromosome 1	15,287,849	15,287,930	93.902	82	3	2	1.26×10^{-23}
mitogenome	306,522	306,603	chromosome 7	26,030,032	26,030,113	96.341	82	3	0	1.62×10^{-27}
mitogenome	325,728	325,809	chromosome 5	19,007,672	19,007,591	100	82	0	0	1.61×10^{-32}
mitogenome	325,728	325,809	chromosome 5	18,907,577	18,907,496	97.561	82	2	0	3.48×10^{-29}
mitogenome	64,735	64,815	chromosome 4	12,657,420	12,657,340	96.296	81	3	0	5.82×10^{-27}
mitogenome	135,574	135,654	chromosome 8	8,294,455	8,294,535	97.531	81	2	0	1.25×10^{-28}
mitogenome	121,443	121,523	chromosome 1	35,973,668	35,973,597	83.951	81	4	2	1.67×10^{-07}
mitogenome	163,058	163,138	chromosome 7	19,139,758	19,139,678	97.531	81	2	0	1.25×10^{-28}
mitogenome	295,401	295,479	chromosome 7	15,916,445	15,916,365	96.296	81	1	2	2.09×10^{-26}
mitogenome	295,401	295,479	chromosome 7	17,165,000	17,164,920	96.296	81	1	2	2.09×10^{-26}
mitogenome	451,835	451,915	chromosome 3	12,150,780	12,150,860	98.765	81	1	0	2.69×10^{-30}
mitogenome	444,838	444,917	chromosome 8	6,716,274	6,716,352	95	80	3	1	3.50×10^{-24}
mitogenome	461,840	461,919	chromosome 1	6,877,065	6,876,986	97.5	80	2	0	4.50×10^{-28}
mitogenome	461,840	461,919	chromosome 1	8,681,307	8,681,386	97.5	80	2	0	4.50×10^{-28}
mitogenome	356,075	356,154	chromosome 1	18,906,642	18,906,721	91.25	80	7	0	9.81×10^{-20}
mitogenome	356,075	356,154	chromosome 1	18,993,966	18,994,045	91.25	80	7	0	9.81×10^{-20}
mitogenome	356,075	356,154	chromosome 1	19,068,077	19,068,156	91.25	80	7	0	9.81×10^{-20}

mitogenome	356,075	356,154	chromosome 1	23,101,591	23,101,670	91.25	80	7	0	9.81×10^{-20}
mitogenome	355,746	355,825	chromosome 7	5,162,631	5,162,552	92.5	80	6	0	2.11×10^{-21}
mitogenome	124,109	124,188	chromosome 3	17,059,849	17,059,928	98.75	80	1	0	9.68×10^{-30}
mitogenome	298,424	298,502	chromosome 4	10,538,740	10,538,818	100	79	0	0	7.48×10^{-31}
mitogenome	421,927	422,005	chromosome 6	27,324,669	27,324,746	97.468	79	1	1	5.82×10^{-27}
mitogenome	5,617	5,695	chromosome 6	24,398,552	24,398,629	93.671	79	4	1	5.86×10^{-22}
mitogenome	5,617	5,695	chromosome 6	26,019,787	26,019,864	93.671	79	4	1	5.86×10^{-22}
mitogenome	296,612	296,690	chromosome 1	56,158,682	56,158,604	96.203	79	3	0	7.53×10^{-26}
mitogenome	302,649	302,726	chromosome 1	13,928,919	13,928,997	94.937	79	3	1	1.26×10^{-23}
mitogenome	225,361	225,438	chromosome 1	47,382,715	47,382,793	94.937	79	3	1	1.26×10^{-23}
mitogenome	444,457	444,535	chromosome 5	24,778,930	24,779,006	93.671	79	3	2	5.86×10^{-22}
mitogenome	444,458	444,536	chromosome 5	24,799,791	24,799,867	93.671	79	3	2	5.86×10^{-22}
mitogenome	56,221	56,299	chromosome 2	1,259,659	1,259,737	96.203	79	3	0	7.53×10^{-26}
mitogenome	295,401	295,479	chromosome 3	5,871,061	5,871,139	96.203	79	3	0	7.53×10^{-26}
mitogenome	357,745	357,823	chromosome 3	2,508,638	2,508,715	96.203	79	2	1	2.71×10^{-25}
mitogenome	250,362	250,439	chromosome 4	18,157,858	18,157,935	98.718	78	1	0	1.25×10^{-28}
mitogenome	300,747	300,824	chromosome 8	4,504,841	4,504,764	96.154	78	3	0	2.71×10^{-25}
mitogenome	78,079	78,156	chromosome 1	33,096,499	33,096,576	94.872	78	4	0	1.26×10^{-23}
mitogenome	393,998	394,072	chromosome 1	32,257,503	32,257,428	87.179	78	5	5	5.95×10^{-12}
mitogenome	157,238	157,315	chromosome 7	11,196,086	11,196,009	100	78	0	0	2.69×10^{-30}
mitogenome	449,279	449,356	chromosome 7	14,581,469	14,581,546	98.718	78	1	0	1.25×10^{-28}
mitogenome	157,238	157,315	chromosome 7	11,196,277	11,196,200	97.436	78	2	0	5.82×10^{-27}
mitogenome	288,830	288,907	chromosome 7	25,496,561	25,496,638	97.436	78	2	0	5.82×10^{-27}
mitogenome	105,164	105,241	chromosome 2	17,165,686	17,165,609	100	78	0	0	2.69×10^{-30}
mitogenome	77,544	77,621	chromosome 2	31,824,448	31,824,525	98.718	78	1	0	1.25×10^{-28}
mitogenome	351,965	352,042	chromosome 2	19,040,053	19,040,126	85.897	78	7	3	7.69×10^{-11}
mitogenome	351,965	352,042	chromosome 2	20,646,025	20,645,952	85.897	78	7	3	7.69×10^{-11}
mitogenome	90,285	90,361	chromosome 4	8,764,077	8,764,001	94.805	77	4	0	4.53×10^{-23}
mitogenome	67,218	67,294	chromosome 1	39,549,364	39,549,288	98.701	77	1	0	4.50×10^{-28}
mitogenome	381,868	381,944	chromosome 1	7,216,736	7,216,660	94.805	77	4	0	4.53×10^{-23}
mitogenome	381,868	381,944	chromosome 1	8,354,709	8,354,785	94.805	77	4	0	4.53×10^{-23}
mitogenome	4,407	4,483	chromosome 1	13,920,672	13,920,596	94.805	77	4	0	4.53×10^{-23}
mitogenome	213,632	213,708	chromosome 7	21,482,815	21,482,891	100	77	0	0	9.68×10^{-30}
mitogenome	319,611	319,687	chromosome 5	23,464,414	23,464,490	97.403	77	2	0	2.09×10^{-26}
mitogenome	429,632	429,708	chromosome 2	14,508,036	14,507,960	92.208	77	6	0	9.81×10^{-20}
mitogenome	498,126	498,202	chromosome 3	2,508,296	2,508,370	97.403	77	0	2	7.53×10^{-26}
mitogenome	181,203	181,279	chromosome 3	15,848,980	15,848,904	96.104	77	3	0	9.74×10^{-25}
mitogenome	101,505	101,580	chromosome 4	8,792,926	8,793,001	97.368	76	2	0	7.53×10^{-26}

mitogenome	351,005	351,080	chromosome 6	16,241,944	16,242,019	98.684	76	1	0	1.62×10^{-27}
mitogenome	122,150	122,225	chromosome 6	11,546,096	11,546,170	96.053	76	2	1	1.26×10^{-23}
mitogenome	201,601	201,676	chromosome 1	39,549,440	39,549,365	90.789	76	7	0	1.64×10^{-17}
mitogenome	332,078	332,152	chromosome 1	40,185,049	40,185,122	90.789	76	4	3	2.12×10^{-16}
mitogenome	195,443	195,518	chromosome 7	14,580,237	14,580,312	98.684	76	1	0	1.62×10^{-27}
mitogenome	437,565	437,640	chromosome 5	23,651,153	23,651,080	97.368	76	0	1	2.71×10^{-25}
mitogenome	384,563	384,638	chromosome 5	11,503,741	11,503,666	96.053	76	3	0	3.50×10^{-24}
mitogenome	429,775	429,850	chromosome 2	19,768,684	19,768,759	94.737	76	4	0	1.63×10^{-22}
mitogenome	345,335	345,410	chromosome 2	20,104,209	20,104,135	92.105	76	5	1	1.27×10^{-18}
mitogenome	164,695	164,770	chromosome 2	20,104,135	20,104,209	92.105	76	5	1	1.27×10^{-18}
mitogenome	495,601	495,676	chromosome 2	20,104,135	20,104,209	92.105	76	5	1	1.27×10^{-18}
mitogenome	157,094	157,169	chromosome 2	20,104,135	20,104,209	90.789	76	6	1	5.91×10^{-17}
mitogenome	345,335	345,410	chromosome 2	20,137,429	20,137,355	89.474	76	7	1	2.75×10^{-15}
mitogenome	164,695	164,770	chromosome 2	20,137,355	20,137,429	89.474	76	7	1	2.75×10^{-15}
mitogenome	495,601	495,676	chromosome 2	20,137,355	20,137,429	89.474	76	7	1	2.75×10^{-15}
mitogenome	157,094	157,169	chromosome 2	20,137,355	20,137,429	88.158	76	8	1	1.28×10^{-13}
mitogenome	168,716	168,791	chromosome 3	3,519,908	3,519,833	98.684	76	1	0	1.62×10^{-27}
mitogenome	159,100	159,175	chromosome 3	33,982,028	33,982,103	96.053	76	3	0	3.50×10^{-24}
mitogenome	332,056	332,131	chromosome 3	30,466,697	30,466,772	94.737	76	4	0	1.63×10^{-22}
mitogenome	159,100	159,175	chromosome 3	33,870,198	33,870,123	94.737	76	4	0	1.63×10^{-22}
mitogenome	110,937	111,011	chromosome 4	14,051,288	14,051,362	100	75	0	0	1.25×10^{-28}
mitogenome	76,703	76,777	chromosome 4	8,978,704	8,978,630	90.667	75	7	0	5.91×10^{-17}
mitogenome	282,296	282,370	chromosome 8	8,254,526	8,254,600	94.667	75	4	0	5.86×10^{-22}
mitogenome	319,613	319,687	chromosome 8	1,982,845	1,982,919	93.333	75	5	0	2.73×10^{-20}
mitogenome	319,613	319,687	chromosome 8	2,010,450	2,010,524	93.333	75	5	0	2.73×10^{-20}
mitogenome	346,043	346,116	chromosome 7	19,139,678	19,139,752	93.333	75	4	1	9.81×10^{-20}
mitogenome	386,083	386,157	chromosome 5	17,955,183	17,955,257	97.333	75	2	0	2.71×10^{-25}
mitogenome	285,365	285,439	chromosome 2	27,860,079	27,860,006	96	75	2	1	4.53×10^{-23}
mitogenome	466,878	466,951	chromosome 3	12,926,703	12,926,777	97.333	75	1	1	9.74×10^{-25}
mitogenome	96,082	96,155	chromosome 4	18,022,169	18,022,096	98.649	74	1	0	2.09×10^{-26}
mitogenome	389,277	389,350	chromosome 4	25,210,401	25,210,328	97.297	74	2	0	9.74×10^{-25}
mitogenome	90,288	90,361	chromosome 4	8,792,716	8,792,643	94.595	74	4	0	2.11×10^{-21}
mitogenome	70,447	70,520	chromosome 4	15,256,550	15,256,623	94.595	74	4	0	2.11×10^{-21}
mitogenome	310,995	311,068	chromosome 8	7,083,736	7,083,663	89.189	74	8	0	9.88×10^{-15}
mitogenome	141,095	141,168	chromosome 6	20,977,412	20,977,339	97.297	74	2	0	9.74×10^{-25}
mitogenome	430,384	430,457	chromosome 6	24,947,547	24,947,615	90.541	74	2	2	9.88×10^{-15}
mitogenome	430,384	430,457	chromosome 6	26,554,354	26,554,422	90.541	74	2	2	9.88×10^{-15}
mitogenome	287,300	287,373	chromosome 1	9,123,459	9,123,386	94.595	74	4	0	2.11×10^{-21}

mitogenome	287,300	287,373	chromosome 1	22,459,873	22,459,946	94.595	74	4	0	2.11×10^{-21}
mitogenome	279,862	279,935	chromosome 7	6,081,230	6,081,303	100	74	0	0	4.50×10^{-28}
mitogenome	184,345	184,418	chromosome 2	16,078,421	16,078,494	94.595	74	4	0	2.11×10^{-21}
mitogenome	194,949	195,022	chromosome 3	31,647,090	31,647,163	97.297	74	2	0	9.74×10^{-25}
mitogenome	201,687	201,759	chromosome 8	5,038,675	5,038,603	86.301	73	10	0	7.69×10^{-11}
mitogenome	336,811	336,883	chromosome 6	6,475,787	6,475,715	100	73	0	0	1.62×10^{-27}
mitogenome	290,333	290,405	chromosome 1	32,993,254	32,993,182	98.63	73	1	0	7.53×10^{-26}
mitogenome	223,062	223,134	chromosome 1	8,505,752	8,505,824	97.26	73	2	0	3.50×10^{-24}
mitogenome	223,062	223,134	chromosome 1	7,025,621	7,025,549	95.89	73	3	0	1.63×10^{-22}
mitogenome	185,822	185,894	chromosome 5	4,562,971	4,563,042	98.63	73	0	1	2.71×10^{-25}
mitogenome	185,822	185,894	chromosome 5	4,457,009	4,457,080	97.26	73	1	1	1.26×10^{-23}
mitogenome	185,822	185,894	chromosome 5	4,515,365	4,515,436	97.26	73	1	1	1.26×10^{-23}
mitogenome	282,180	282,252	chromosome 2	33,213,114	33,213,186	98.63	73	1	0	7.53×10^{-26}
mitogenome	351,413	351,485	chromosome 2	13,661,714	13,661,786	97.26	73	2	0	3.50×10^{-24}
mitogenome	148,283	148,354	chromosome 2	10,619,689	10,619,617	95.89	73	2	1	5.86×10^{-22}
mitogenome	279,540	279,612	chromosome 3	1,676,313	1,676,385	97.26	73	2	0	3.50×10^{-24}
mitogenome	279,540	279,612	chromosome 3	1,886,949	1,887,021	97.26	73	2	0	3.50×10^{-24}
mitogenome	279,540	279,612	chromosome 3	5,237,748	5,237,676	97.26	73	2	0	3.50×10^{-24}
mitogenome	340,066	340,137	chromosome 4	25,535,422	25,535,493	100	72	0	0	5.82×10^{-27}
mitogenome	309,221	309,292	chromosome 8	1,419,517	1,419,588	94.444	72	4	0	2.73×10^{-20}
mitogenome	219,753	219,824	chromosome 7	1,055,204	1,055,133	100	72	0	0	5.82×10^{-27}
mitogenome	301,608	301,679	chromosome 7	18,774,518	18,774,447	98.611	72	1	0	2.71×10^{-25}
mitogenome	145,712	145,783	chromosome 2	21,252,233	21,252,304	98.611	72	1	0	2.71×10^{-25}
mitogenome	184,348	184,418	chromosome 4	24,215,303	24,215,233	95.775	71	3	0	2.11×10^{-21}
mitogenome	90	160	chromosome 1	25,065,095	25,065,165	100	71	0	0	2.09×10^{-26}
mitogenome	333,004	333,074	chromosome 1	10,933,561	10,933,491	98.592	71	1	0	9.74×10^{-25}
mitogenome	90	160	chromosome 1	22,291,473	22,291,403	98.592	71	1	0	9.74×10^{-25}
mitogenome	184,348	184,418	chromosome 2	19,518,436	19,518,366	97.183	71	2	0	4.53×10^{-23}
mitogenome	184,348	184,418	chromosome 2	31,302,385	31,302,315	95.775	71	3	0	2.11×10^{-21}
mitogenome	112,155	112,225	chromosome 2	1,997,884	1,997,953	91.549	71	5	1	7.64×10^{-16}
mitogenome	193,594	193,664	chromosome 3	2,454,131	2,454,061	95.775	71	3	0	2.11×10^{-21}
mitogenome	138,353	138,423	chromosome 3	4,789,928	4,789,858	90.141	71	7	0	9.88×10^{-15}
mitogenome	147,495	147,563	chromosome 4	15,967,974	15,968,043	94.286	70	3	1	1.27×10^{-18}
mitogenome	413,862	413,931	chromosome 1	35,835,197	35,835,266	98.571	70	1	0	3.50×10^{-24}
mitogenome	219,755	219,824	chromosome 7	283,335	283,266	100	70	0	0	7.53×10^{-26}
mitogenome	226,916	226,985	chromosome 7	25,446,210	25,446,141	100	70	0	0	7.53×10^{-26}
mitogenome	144,212	144,281	chromosome 5	9,555,168	9,555,099	95.714	70	3	0	7.59×10^{-21}
mitogenome	144,212	144,281	chromosome 5	9,556,751	9,556,682	95.714	70	3	0	7.59×10^{-21}

mitogenome	144,212	144,281	chromosome 5	9,529,598	9,529,531	90	70	5	1	1.28×10^{-13}
mitogenome	295,383	295,451	chromosome 4	12,989,798	12,989,731	97.101	69	1	1	2.11×10^{-21}
mitogenome	324,514	324,582	chromosome 4	1,615,699	1,615,767	91.304	69	6	0	2.75×10^{-15}
mitogenome	421,932	422,000	chromosome 6	11,335,939	11,335,871	98.551	69	1	0	1.26×10^{-23}
mitogenome	190,873	190,941	chromosome 1	13,959,953	13,960,021	94.203	69	4	0	1.27×10^{-18}
mitogenome	94,579	94,647	chromosome 7	26,030,269	26,030,337	95.652	69	3	0	2.73×10^{-20}
mitogenome	287,016	287,083	chromosome 5	5,443,413	5,443,480	95.652	69	1	2	9.81×10^{-20}
mitogenome	209,462	209,530	chromosome 3	11,934,541	11,934,609	95.652	69	3	0	2.73×10^{-20}
mitogenome	184,350	184,418	chromosome 3	30,736,335	30,736,403	94.203	69	4	0	1.27×10^{-18}
mitogenome	450,667	450,735	chromosome 3	30,466,869	30,466,937	91.304	69	6	0	2.75×10^{-15}
mitogenome	198,307	198,374	chromosome 4	3,937,765	3,937,832	98.529	68	1	0	4.53×10^{-23}
mitogenome	184,351	184,418	chromosome 6	23,011,020	23,010,953	95.588	68	3	0	9.81×10^{-20}
mitogenome	82,601	82,668	chromosome 1	33,096,487	33,096,420	98.529	68	1	0	4.53×10^{-23}
mitogenome	291,371	291,438	chromosome 1	27,419,355	27,419,288	97.059	68	2	0	2.11×10^{-21}
mitogenome	164,553	164,620	chromosome 7	12,154,551	12,154,618	95.588	68	3	0	9.81×10^{-20}
mitogenome	287,016	287,083	chromosome 5	6,694,828	6,694,761	98.529	68	1	0	4.53×10^{-23}
mitogenome	385,815	385,882	chromosome 5	2,877,568	2,877,503	94.118	68	2	1	1.64×10^{-17}
mitogenome	90,185	90,252	chromosome 3	14,809,921	14,809,854	98.529	68	1	0	4.53×10^{-23}
mitogenome	323,113	323,180	chromosome 3	31,326,623	31,326,690	95.588	68	3	0	9.81×10^{-20}
mitogenome	323,018	323,083	chromosome 4	19,623,496	19,623,431	91.045	67	4	2	1.28×10^{-13}
mitogenome	3,530	3,596	chromosome 1	7,300,349	7,300,283	98.507	67	1	0	1.63×10^{-22}
mitogenome	3,530	3,596	chromosome 1	8,290,668	8,290,734	98.507	67	1	0	1.63×10^{-22}
mitogenome	353,419	353,485	chromosome 1	20,098,829	20,098,895	98.507	67	1	0	1.63×10^{-22}
mitogenome	353,419	353,485	chromosome 1	24,149,353	24,149,419	98.507	67	1	0	1.63×10^{-22}
mitogenome	378,101	378,167	chromosome 1	31,153,829	31,153,894	95.522	67	2	1	1.27×10^{-18}
mitogenome	486,796	486,862	chromosome 7	12,520,133	12,520,199	98.507	67	1	0	1.63×10^{-22}
mitogenome	455,844	455,910	chromosome 2	11,000,283	11,000,217	100	67	0	0	3.50×10^{-24}
mitogenome	323,018	323,083	chromosome 4	19,585,815	19,585,750	96.97	66	2	0	2.73×10^{-20}
mitogenome	226,110	226,175	chromosome 8	13,063,395	13,063,460	100	66	0	0	1.26×10^{-23}
mitogenome	102,094	102,159	chromosome 8	4,400,040	4,400,104	95.455	66	2	1	4.57×10^{-18}
mitogenome	183,403	183,468	chromosome 1	12,010,736	12,010,801	98.485	66	1	0	5.86×10^{-22}
mitogenome	183,403	183,468	chromosome 1	12,506,074	12,506,139	98.485	66	1	0	5.86×10^{-22}
mitogenome	169,574	169,639	chromosome 1	19,227,836	19,227,900	98.485	66	0	1	2.11×10^{-21}
mitogenome	169,574	169,639	chromosome 1	23,270,399	23,270,463	98.485	66	0	1	2.11×10^{-21}
mitogenome	226,110	226,175	chromosome 1	874,750	874,815	96.97	66	2	0	2.73×10^{-20}
mitogenome	111,408	111,473	chromosome 1	44,441,176	44,441,237	92.424	66	1	1	3.55×10^{-14}
mitogenome	297,072	297,137	chromosome 7	3,087,608	3,087,543	96.97	66	2	0	2.73×10^{-20}
mitogenome	410,144	410,209	chromosome 7	11,196,342	11,196,277	96.97	66	2	0	2.73×10^{-20}

mitogenome	156,605	156,670	chromosome 7	11,196,277	11,196,342	96.97	66	2	0	2.73×10^{-20}
mitogenome	226,110	226,175	chromosome 5	21,599,259	21,599,194	96.97	66	2	0	2.73×10^{-20}
mitogenome	94,632	94,697	chromosome 5	26,393,142	26,393,207	93.939	66	4	0	5.91×10^{-17}
mitogenome	226,110	226,175	chromosome 2	14,248,786	14,248,851	96.97	66	2	0	2.73×10^{-20}
mitogenome	138,381	138,443	chromosome 2	8,686,416	8,686,351	93.939	66	1	2	7.64×10^{-16}
mitogenome	188,613	188,678	chromosome 3	10,452,185	10,452,250	95.455	66	3	0	1.27×10^{-18}
mitogenome	384,419	384,482	chromosome 6	12,238,720	12,238,784	95.385	65	2	1	1.64×10^{-17}
mitogenome	317,246	317,310	chromosome 6	12,779,785	12,779,721	92.308	65	5	0	9.88×10^{-15}
mitogenome	73,512	73,576	chromosome 1	56,158,604	56,158,540	100	65	0	0	4.53×10^{-23}
mitogenome	54,060	54,124	chromosome 1	55,007,707	55,007,771	98.462	65	1	0	2.11×10^{-21}
mitogenome	64,234	64,298	chromosome 7	6,212,061	6,212,125	100	65	0	0	4.53×10^{-23}
mitogenome	437,120	437,184	chromosome 2	12,317,899	12,317,835	93.846	65	4	0	2.12×10^{-16}
mitogenome	391,155	391,218	chromosome 2	33,926,968	33,926,905	90.769	65	4	2	1.65×10^{-12}
mitogenome	386,843	386,906	chromosome 2	33,926,968	33,926,905	90.769	65	4	2	1.65×10^{-12}
mitogenome	271,006	271,067	chromosome 4	24,060,454	24,060,392	90.625	64	3	3	2.14×10^{-11}
mitogenome	380,392	380,454	chromosome 1	45,945,248	45,945,186	95.312	64	1	2	5.91×10^{-17}
mitogenome	297,074	297,137	chromosome 7	3,431,517	3,431,454	96.875	64	2	0	3.53×10^{-19}
mitogenome	161,462	161,525	chromosome 5	10,257,459	10,257,396	98.438	64	1	0	7.59×10^{-21}
mitogenome	137,933	137,995	chromosome 8	15,559,692	15,559,754	96.825	63	2	0	1.27×10^{-18}
mitogenome	137,933	137,995	chromosome 8	16,367,827	16,367,889	96.825	63	2	0	1.27×10^{-18}
mitogenome	105,453	105,515	chromosome 6	5,708,183	5,708,244	95.238	63	2	1	2.12×10^{-16}
mitogenome	490,095	490,157	chromosome 1	8,868,466	8,868,404	100	63	0	0	5.86×10^{-22}
mitogenome	490,095	490,157	chromosome 1	6,681,999	6,682,061	98.413	63	1	0	2.73×10^{-20}
mitogenome	69,400	69,462	chromosome 1	37,668,162	37,668,224	95.238	63	3	0	5.91×10^{-17}
mitogenome	122,657	122,719	chromosome 1	275,909	275,971	93.651	63	4	0	2.75×10^{-15}
mitogenome	349,013	349,075	chromosome 1	41,782,968	41,782,906	90.476	63	6	0	5.95×10^{-12}
mitogenome	391,222	391,284	chromosome 7	5,592,941	5,593,003	96.825	63	2	0	1.27×10^{-18}
mitogenome	496,314	496,376	chromosome 2	12,317,837	12,317,898	92.063	63	4	1	4.60×10^{-13}
mitogenome	443,692	443,754	chromosome 3	29,414,640	29,414,702	98.413	63	1	0	2.73×10^{-20}
mitogenome	354,194	354,255	chromosome 3	17,814,199	17,814,261	96.825	63	1	1	4.57×10^{-18}
mitogenome	338,060	338,120	chromosome 4	25,533,012	25,532,951	96.774	62	1	1	1.64×10^{-17}
mitogenome	504,749	504,810	chromosome 1	9,848,485	9,848,424	100	62	0	0	2.11×10^{-21}
mitogenome	504,749	504,810	chromosome 1	10,976,265	10,976,204	100	62	0	0	2.11×10^{-21}
mitogenome	463,180	463,241	chromosome 1	32,257,451	32,257,390	100	62	0	0	2.11×10^{-21}
mitogenome	456,416	456,477	chromosome 1	4,294,387	4,294,448	98.387	62	1	0	9.81×10^{-20}
mitogenome	194,304	194,365	chromosome 1	49,373,032	49,372,971	98.387	62	1	0	9.81×10^{-20}
mitogenome	462,984	463,045	chromosome 1	16,117,612	16,117,673	96.774	62	2	0	4.57×10^{-18}
mitogenome	85,371	85,432	chromosome 1	33,096,420	33,096,359	96.774	62	2	0	4.57×10^{-18}

mitogenome	58,985	59,046	chromosome 7	6,211,741	6,211,802	98.387	62	1	0	9.81×10^{-20}
mitogenome	72,397	72,457	chromosome 5	6,166,746	6,166,685	98.387	62	0	1	3.53×10^{-19}
mitogenome	378,424	378,485	chromosome 2	33,685,989	33,685,928	98.387	62	1	0	9.81×10^{-20}
mitogenome	149,467	149,527	chromosome 2	33,527,027	33,527,088	93.548	62	3	1	3.55×10^{-14}
mitogenome	302,117	302,178	chromosome 3	1,676,254	1,676,315	96.774	62	2	0	4.57×10^{-18}
mitogenome	302,117	302,178	chromosome 3	1,886,890	1,886,951	96.774	62	2	0	4.57×10^{-18}
mitogenome	302,117	302,178	chromosome 3	5,237,807	5,237,746	95.161	62	3	0	2.12×10^{-16}
mitogenome	387,125	387,186	chromosome 3	8,562,349	8,562,410	95.161	62	3	0	2.12×10^{-16}
mitogenome	383,204	383,264	chromosome 6	20,528,801	20,528,741	100	61	0	0	7.59×10^{-21}
mitogenome	383,204	383,264	chromosome 6	20,237,747	20,237,807	98.361	61	1	0	3.53×10^{-19}
mitogenome	315,575	315,635	chromosome 1	30,314,934	30,314,994	95.082	61	3	0	7.64×10^{-16}
mitogenome	311,890	311,950	chromosome 1	26,376,464	26,376,524	90.164	61	6	0	7.69×10^{-11}
mitogenome	317,276	317,336	chromosome 7	9,925,165	9,925,105	96.721	61	2	0	1.64×10^{-17}
mitogenome	432,464	432,522	chromosome 4	3,428,008	3,427,949	95	60	2	1	9.88×10^{-15}
mitogenome	433,195	433,254	chromosome 6	21,735,272	21,735,213	98.333	60	1	0	1.27×10^{-18}
mitogenome	195,717	195,776	chromosome 6	2,600,595	2,600,654	93.333	60	4	0	1.28×10^{-13}
mitogenome	204,254	204,313	chromosome 7	13,171,988	13,172,047	96.667	60	2	0	5.91×10^{-17}
mitogenome	338,923	338,982	chromosome 2	16,834,693	16,834,634	98.333	60	1	0	1.27×10^{-18}
mitogenome	338,923	338,982	chromosome 2	12,317,934	12,317,993	96.667	60	2	0	5.91×10^{-17}
mitogenome	195,888	195,947	chromosome 3	34,310,804	34,310,745	96.667	60	2	0	5.91×10^{-17}
mitogenome	328,853	328,912	chromosome 3	30,467,097	30,467,154	95	60	1	2	9.88×10^{-15}
mitogenome	195,597	195,655	chromosome 8	17,757,877	17,757,935	100	59	0	0	9.81×10^{-20}
mitogenome	6,545	6,603	chromosome 8	9,232,247	9,232,305	94.915	59	3	0	9.88×10^{-15}
mitogenome	289,701	289,759	chromosome 1	32,993,181	32,993,123	100	59	0	0	9.81×10^{-20}
mitogenome	462,174	462,232	chromosome 1	55,248,221	55,248,163	96.61	59	2	0	2.12×10^{-16}
mitogenome	357,594	357,652	chromosome 1	27,108,232	27,108,290	93.22	59	4	0	4.60×10^{-13}
mitogenome	241,454	241,511	chromosome 5	23,651,137	23,651,080	91.525	59	3	2	7.69×10^{-11}
mitogenome	150,627	150,685	chromosome 2	17,473,127	17,473,184	94.915	59	2	1	3.55×10^{-14}
mitogenome	150,627	150,685	chromosome 2	21,730,399	21,730,456	94.915	59	2	1	3.55×10^{-14}
mitogenome	485,070	485,127	chromosome 4	17,555,067	17,555,010	96.552	58	2	0	7.64×10^{-16}
mitogenome	135,167	135,223	chromosome 1	52,929,169	52,929,225	94.828	58	1	2	1.28×10^{-13}
mitogenome	193,477	193,534	chromosome 7	17,059,413	17,059,356	96.552	58	2	0	7.64×10^{-16}
mitogenome	193,477	193,534	chromosome 7	15,799,942	15,799,885	94.828	58	3	0	3.55×10^{-14}
mitogenome	149,471	149,527	chromosome 2	33,534,176	33,534,233	94.828	58	2	1	1.28×10^{-13}
mitogenome	147,973	148,030	chromosome 2	16,834,579	16,834,636	93.103	58	4	0	1.65×10^{-12}
mitogenome	454,697	454,754	chromosome 3	11,934,540	11,934,483	98.276	58	1	0	1.64×10^{-17}
mitogenome	113,071	113,128	chromosome 3	19,377,571	19,377,628	96.552	58	2	0	7.64×10^{-16}
mitogenome	223,131	223,187	chromosome 1	7,024,825	7,024,769	98.246	57	1	0	5.91×10^{-17}

mitogenome	223,131	223,187	chromosome 1	8,506,548	8,506,604	98.246	57	1	0	5.91×10^{-17}
mitogenome	70,209	70,265	chromosome 1	55,521,632	55,521,688	96.491	57	2	0	2.75×10^{-15}
mitogenome	288,252	288,308	chromosome 1	38,862,660	38,862,605	94.737	57	2	1	4.60×10^{-13}
mitogenome	322,768	322,824	chromosome 7	10,515,456	10,515,400	94.737	57	3	0	1.28×10^{-13}
mitogenome	188,566	188,622	chromosome 5	5,404,460	5,404,404	96.491	57	2	0	2.75×10^{-15}
mitogenome	188,566	188,622	chromosome 5	6,792,247	6,792,303	96.491	57	2	0	2.75×10^{-15}
mitogenome	298,867	298,923	chromosome 2	34,882,269	34,882,325	98.246	57	1	0	5.91×10^{-17}
mitogenome	4,854	4,909	chromosome 3	9,609,829	9,609,885	96.491	57	1	1	9.88×10^{-15}
mitogenome	487,624	487,680	chromosome 3	12,700,684	12,700,629	96.491	57	1	1	9.88×10^{-15}
mitogenome	193,269	193,324	chromosome 4	27,636,609	27,636,664	100	56	0	0	4.57×10^{-18}
mitogenome	328,853	328,908	chromosome 4	25,963,901	25,963,846	96.429	56	2	0	9.88×10^{-15}
mitogenome	502,474	502,529	chromosome 8	1,535,869	1,535,924	100	56	0	0	4.57×10^{-18}
mitogenome	372,563	372,618	chromosome 6	15,005,248	15,005,193	92.857	56	4	0	2.14×10^{-11}
mitogenome	372,563	372,618	chromosome 6	16,005,381	16,005,326	91.071	56	5	0	9.95×10^{-10}
mitogenome	75,783	75,838	chromosome 1	15,578,685	15,578,740	98.214	56	1	0	2.12×10^{-16}
mitogenome	433,471	433,526	chromosome 1	32,257,445	32,257,390	96.429	56	2	0	9.88×10^{-15}
mitogenome	274,126	274,181	chromosome 1	41,782,913	41,782,968	94.643	56	3	0	4.60×10^{-13}
mitogenome	131,654	131,709	chromosome 1	44,442,169	44,442,114	94.643	56	3	0	4.60×10^{-13}
mitogenome	281,431	281,486	chromosome 5	5,404,404	5,404,349	98.214	56	1	0	2.12×10^{-16}
mitogenome	378,630	378,685	chromosome 2	1,751,198	1,751,143	98.214	56	1	0	2.12×10^{-16}
mitogenome	152,132	152,187	chromosome 2	10,639,986	10,639,932	98.214	56	0	1	7.64×10^{-16}
mitogenome	113,066	113,121	chromosome 3	2,508,716	2,508,771	98.214	56	1	0	2.12×10^{-16}
mitogenome	156,413	156,467	chromosome 4	14,107,126	14,107,180	98.182	55	1	0	7.64×10^{-16}
mitogenome	64,176	64,230	chromosome 7	6,211,966	6,212,020	100	55	0	0	1.64×10^{-17}
mitogenome	228,628	228,682	chromosome 5	4,832,420	4,832,366	98.182	55	1	0	7.64×10^{-16}
mitogenome	228,628	228,682	chromosome 5	75,293	75,347	96.364	55	2	0	3.55×10^{-14}
mitogenome	228,628	228,682	chromosome 5	2,270,799	2,270,745	96.364	55	2	0	3.55×10^{-14}
mitogenome	391,084	391,138	chromosome 2	22,270,320	22,270,374	100	55	0	0	1.64×10^{-17}
mitogenome	305,775	305,829	chromosome 2	10,619,614	10,619,560	96.364	55	2	0	3.55×10^{-14}
mitogenome	460,311	460,365	chromosome 3	9,823,603	9,823,549	98.182	55	1	0	7.64×10^{-16}
mitogenome	460,311	460,365	chromosome 3	10,082,327	10,082,273	98.182	55	1	0	7.64×10^{-16}
mitogenome	133,154	133,208	chromosome 3	23,881,048	23,880,994	90.909	55	5	0	3.58×10^{-09}
mitogenome	169,894	169,947	chromosome 4	7,559,543	7,559,596	98.148	54	1	0	2.75×10^{-15}
mitogenome	324,859	324,912	chromosome 4	9,463,948	9,463,895	98.148	54	1	0	2.75×10^{-15}
mitogenome	5,602	5,655	chromosome 8	1,887,096	1,887,043	98.148	54	1	0	2.75×10^{-15}
mitogenome	274,965	275,018	chromosome 8	6,728,135	6,728,187	98.148	54	0	1	9.88×10^{-15}
mitogenome	58,601	58,654	chromosome 6	23,450,234	23,450,181	92.593	54	4	0	2.77×10^{-10}
mitogenome	178,605	178,658	chromosome 1	27,189,315	27,189,368	96.296	54	2	0	1.28×10^{-13}

mitogenome	106,281	106,334	chromosome 1	39,354,456	39,354,508	96.296	54	1	1	4.60×10^{-13}
mitogenome	421,475	421,528	chromosome 2	1,072,566	1,072,513	94.444	54	3	0	5.95×10^{-12}
mitogenome	140,156	140,209	chromosome 3	12,994,388	12,994,441	94.444	54	3	0	5.95×10^{-12}
mitogenome	389,659	389,712	chromosome 3	17,421,760	17,421,707	92.593	54	4	0	2.77×10^{-10}
mitogenome	495,496	495,548	chromosome 4	5,051,533	5,051,585	88.679	53	6	0	2.15×10^{-06}
mitogenome	338,955	339,007	chromosome 8	13,562,029	13,561,977	96.226	53	2	0	4.60×10^{-13}
mitogenome	60,712	60,764	chromosome 1	25,840,652	25,840,600	100	53	0	0	2.12×10^{-16}
mitogenome	256,380	256,432	chromosome 7	3,087,660	3,087,608	98.113	53	1	0	9.88×10^{-15}
mitogenome	163,220	163,272	chromosome 7	11,885,904	11,885,852	98.113	53	1	0	9.88×10^{-15}
mitogenome	345,909	345,961	chromosome 7	11,885,852	11,885,904	98.113	53	1	0	9.88×10^{-15}
mitogenome	163,220	163,272	chromosome 7	11,896,836	11,896,784	98.113	53	1	0	9.88×10^{-15}
mitogenome	345,909	345,961	chromosome 7	11,896,784	11,896,836	98.113	53	1	0	9.88×10^{-15}
mitogenome	256,385	256,437	chromosome 7	3,431,566	3,431,515	98.113	53	0	1	3.55×10^{-14}
mitogenome	281,431	281,483	chromosome 5	6,792,303	6,792,355	100	53	0	0	2.12×10^{-16}
mitogenome	467,918	467,970	chromosome 5	11,889,388	11,889,439	96.226	53	1	1	1.65×10^{-12}
mitogenome	459,990	460,042	chromosome 2	1,751,562	1,751,614	94.34	53	3	0	2.14×10^{-11}
mitogenome	119,975	120,027	chromosome 3	27,334,893	27,334,945	96.226	53	2	0	4.60×10^{-13}
mitogenome	99,918	99,969	chromosome 6	24,706,237	24,706,186	100	52	0	0	7.64×10^{-16}
mitogenome	99,918	99,969	chromosome 6	26,363,181	26,363,130	100	52	0	0	7.64×10^{-16}
mitogenome	329,611	329,662	chromosome 6	8,053,311	8,053,362	92.308	52	4	0	3.58×10^{-09}
mitogenome	249,839	249,890	chromosome 1	55,853,280	55,853,229	100	52	0	0	7.64×10^{-16}
mitogenome	381,100	381,151	chromosome 1	27,108,166	27,108,217	94.231	52	3	0	7.69×10^{-11}
mitogenome	144,742	144,793	chromosome 7	22,390,777	22,390,828	96.154	52	2	0	1.65×10^{-12}
mitogenome	273,946	273,997	chromosome 5	18,107,875	18,107,926	98.077	52	1	0	3.55×10^{-14}
mitogenome	288,173	288,224	chromosome 5	661,271	661,220	96.154	52	2	0	1.65×10^{-12}
mitogenome	457,310	457,361	chromosome 5	12,005,520	12,005,571	94.231	52	3	0	7.69×10^{-11}
mitogenome	393,869	393,920	chromosome 5	13,352,699	13,352,648	94.231	52	3	0	7.69×10^{-11}
mitogenome	460,711	460,762	chromosome 2	23,322,099	23,322,150	98.077	52	1	0	3.55×10^{-14}
mitogenome	297,231	297,282	chromosome 2	33,246,996	33,246,945	96.154	52	2	0	1.65×10^{-12}
mitogenome	192,845	192,896	chromosome 3	10,452,303	10,452,252	100	52	0	0	7.64×10^{-16}
mitogenome	297,231	297,282	chromosome 3	14,809,972	14,809,921	98.077	52	1	0	3.55×10^{-14}
mitogenome	325,118	325,169	chromosome 3	30,466,647	30,466,698	96.154	52	2	0	1.65×10^{-12}
mitogenome	225,332	225,383	chromosome 3	31,646,869	31,646,920	96.154	52	2	0	1.65×10^{-12}
mitogenome	89,703	89,753	chromosome 8	3,170,282	3,170,232	98.039	51	1	0	1.28×10^{-13}
mitogenome	317,972	318,022	chromosome 8	8,236,928	8,236,878	96.078	51	2	0	5.95×10^{-12}
mitogenome	438,979	439,029	chromosome 8	18,576,195	18,576,245	96.078	51	2	0	5.95×10^{-12}
mitogenome	315,735	315,785	chromosome 7	2,077,401	2,077,351	98.039	51	1	0	1.28×10^{-13}
mitogenome	281,364	281,414	chromosome 2	27,860,104	27,860,154	100	51	0	0	2.75×10^{-15}

mitogenome	403,429	403,479	chromosome 3	18,444,554	18,444,604	100	51	0	0	2.75×10^{-15}
mitogenome	6,623	6,672	chromosome 5	4,721,814	4,721,863	96	50	2	0	2.14×10^{-11}
mitogenome	142,749	142,798	chromosome 2	567,198	567,149	100	50	0	0	9.88×10^{-15}
mitogenome	138,853	138,902	chromosome 2	34,945,201	34,945,250	96	50	2	0	2.14×10^{-11}
mitogenome	503,306	503,352	chromosome 4	27,048,333	27,048,285	95.918	49	0	1	2.77×10^{-10}
mitogenome	164,752	164,800	chromosome 1	42,012,299	42,012,251	100	49	0	0	3.55×10^{-14}
mitogenome	345,305	345,353	chromosome 1	42,012,251	42,012,299	100	49	0	0	3.55×10^{-14}
mitogenome	345,305	345,353	chromosome 1	43,312,691	43,312,643	100	49	0	0	3.55×10^{-14}
mitogenome	164,752	164,800	chromosome 1	43,312,643	43,312,691	100	49	0	0	3.55×10^{-14}
mitogenome	101,957	102,005	chromosome 1	56,301,809	56,301,761	93.878	49	3	0	3.58×10^{-09}
mitogenome	354,802	354,850	chromosome 7	20,722,372	20,722,420	95.918	49	2	0	7.69×10^{-11}
mitogenome	2,490	2,538	chromosome 5	5,574,710	5,574,758	95.918	49	2	0	7.69×10^{-11}
mitogenome	71,284	71,332	chromosome 3	26,177,317	26,177,269	97.959	49	1	0	1.65×10^{-12}
mitogenome	63,006	63,053	chromosome 3	5,894,815	5,894,768	95.918	49	0	2	2.77×10^{-10}
mitogenome	121,871	121,918	chromosome 4	25,073,535	25,073,488	100	48	0	0	1.28×10^{-13}
mitogenome	73,968	74,015	chromosome 8	6,912,662	6,912,615	95.833	48	2	0	2.77×10^{-10}
mitogenome	374,955	375,002	chromosome 8	5,782,865	5,782,912	93.75	48	3	0	1.29×10^{-08}
mitogenome	374,955	375,002	chromosome 8	5,974,585	5,974,632	93.75	48	3	0	1.29×10^{-08}
mitogenome	458,386	458,433	chromosome 1	52,950,039	52,950,086	100	48	0	0	1.28×10^{-13}
mitogenome	395,546	395,593	chromosome 1	48,440,220	48,440,267	95.833	48	2	0	2.77×10^{-10}
mitogenome	437,601	437,648	chromosome 1	50,439,561	50,439,514	95.833	48	2	0	2.77×10^{-10}
mitogenome	394,704	394,751	chromosome 1	50,439,561	50,439,514	95.833	48	2	0	2.77×10^{-10}
mitogenome	390,248	390,295	chromosome 1	50,439,561	50,439,514	95.833	48	2	0	2.77×10^{-10}
mitogenome	186,166	186,213	chromosome 1	50,439,514	50,439,561	95.833	48	2	0	2.77×10^{-10}
mitogenome	241,472	241,519	chromosome 1	50,439,561	50,439,514	95.833	48	2	0	2.77×10^{-10}
mitogenome	295,399	295,444	chromosome 1	9,123,457	9,123,504	95.833	48	0	2	9.95×10^{-10}
mitogenome	435,628	435,675	chromosome 1	21,809,562	21,809,609	93.75	48	3	0	1.29×10^{-08}
mitogenome	435,628	435,675	chromosome 1	25,579,385	25,579,338	93.75	48	3	0	1.29×10^{-08}
mitogenome	295,399	295,444	chromosome 1	22,459,875	22,459,828	93.75	48	1	2	4.63×10^{-08}
mitogenome	348,091	348,137	chromosome 5	29,159,194	29,159,148	93.75	48	1	2	4.63×10^{-08}
mitogenome	164,745	164,791	chromosome 1	35,214,998	35,214,952	100	47	0	0	4.60×10^{-13}
mitogenome	345,314	345,360	chromosome 1	35,214,952	35,214,998	100	47	0	0	4.60×10^{-13}
mitogenome	157,144	157,190	chromosome 1	35,214,998	35,214,952	100	47	0	0	4.60×10^{-13}
mitogenome	333,090	333,136	chromosome 7	2,040,761	2,040,807	97.872	47	1	0	2.14×10^{-11}
mitogenome	148,008	148,054	chromosome 7	13,038,488	13,038,533	95.745	47	1	1	3.58×10^{-09}
mitogenome	394,698	394,743	chromosome 5	23,651,126	23,651,080	95.745	47	1	1	3.58×10^{-09}
mitogenome	260,059	260,105	chromosome 3	5,894,806	5,894,762	95.745	47	0	2	3.58×10^{-09}
mitogenome	335,165	335,210	chromosome 4	10,333,389	10,333,434	97.826	46	1	0	7.69×10^{-11}

mitogenome	260,795	260,839	chromosome 6	9,986,358	9,986,403	95.652	46	1	1	1.29×10^{-08}
mitogenome	260,795	260,839	chromosome 6	9,987,802	9,987,847	95.652	46	1	1	1.29×10^{-08}
mitogenome	260,795	260,839	chromosome 6	9,989,300	9,989,345	95.652	46	1	1	1.29×10^{-08}
mitogenome	319,047	319,092	chromosome 1	31,700,475	31,700,430	93.478	46	3	0	1.67×10^{-07}
mitogenome	288,173	288,218	chromosome 5	1,726,226	1,726,271	100	46	0	0	1.65×10^{-12}
mitogenome	440,150	440,195	chromosome 5	10,257,398	10,257,353	100	46	0	0	1.65×10^{-12}
mitogenome	2,493	2,538	chromosome 5	6,567,800	6,567,755	97.826	46	1	0	7.69×10^{-11}
mitogenome	478,475	478,520	chromosome 2	8,812,180	8,812,135	100	46	0	0	1.65×10^{-12}
mitogenome	503,308	503,352	chromosome 4	26,174,368	26,174,412	97.778	45	1	0	2.77×10^{-10}
mitogenome	376,904	376,948	chromosome 1	31,700,431	31,700,387	95.556	45	2	0	1.29×10^{-08}
mitogenome	133,040	133,084	chromosome 5	21,639,430	21,639,474	100	45	0	0	5.95×10^{-12}
mitogenome	181,699	181,743	chromosome 5	13,458,222	13,458,266	97.778	45	1	0	2.77×10^{-10}
mitogenome	133,040	133,084	chromosome 2	5,285,371	5,285,415	100	45	0	0	5.95×10^{-12}
mitogenome	133,040	133,084	chromosome 2	7,383,551	7,383,507	100	45	0	0	5.95×10^{-12}
mitogenome	133,040	133,084	chromosome 2	7,403,393	7,403,349	100	45	0	0	5.95×10^{-12}
mitogenome	144,825	144,869	chromosome 2	34,882,268	34,882,225	97.778	45	0	1	9.95×10^{-10}
mitogenome	458,953	458,996	chromosome 4	20,405,722	20,405,765	100	44	0	0	2.14×10^{-11}
mitogenome	458,953	458,996	chromosome 8	10,281,703	10,281,660	100	44	0	0	2.14×10^{-11}
mitogenome	458,953	458,996	chromosome 6	15,051,446	15,051,489	100	44	0	0	2.14×10^{-11}
mitogenome	458,953	458,996	chromosome 6	16,055,727	16,055,770	100	44	0	0	2.14×10^{-11}
mitogenome	458,953	458,996	chromosome 6	22,102,408	22,102,365	100	44	0	0	2.14×10^{-11}
mitogenome	227,433	227,476	chromosome 1	15,417,568	15,417,525	100	44	0	0	2.14×10^{-11}
mitogenome	122,706	122,749	chromosome 1	37,667,960	37,668,003	97.727	44	1	0	9.95×10^{-10}
mitogenome	458,953	458,996	chromosome 7	16,089,512	16,089,555	100	44	0	0	2.14×10^{-11}
mitogenome	458,953	458,996	chromosome 2	8,035,415	8,035,458	100	44	0	0	2.14×10^{-11}
mitogenome	458,953	458,996	chromosome 2	34,298,200	34,298,243	100	44	0	0	2.14×10^{-11}
mitogenome	458,953	458,996	chromosome 2	37,188,817	37,188,774	100	44	0	0	2.14×10^{-11}
mitogenome	334,312	334,355	chromosome 2	29,146,409	29,146,366	97.727	44	1	0	9.95×10^{-10}
mitogenome	458,953	458,996	chromosome 3	26,445,120	26,445,163	100	44	0	0	2.14×10^{-11}
mitogenome	372,581	372,623	chromosome 8	8,433,656	8,433,698	100	43	0	0	7.69×10^{-11}
mitogenome	221,694	221,736	chromosome 1	52,312,500	52,312,542	97.674	43	1	0	3.58×10^{-09}
mitogenome	335,795	335,837	chromosome 7	14,257,407	14,257,449	97.674	43	1	0	3.58×10^{-09}
mitogenome	182,632	182,674	chromosome 7	24,478,256	24,478,298	97.674	43	1	0	3.58×10^{-09}
mitogenome	148,985	149,027	chromosome 3	34,300,493	34,300,451	97.674	43	1	0	3.58×10^{-09}
mitogenome	458,744	458,786	chromosome 3	16,069,097	16,069,139	95.349	43	2	0	1.67×10^{-07}
mitogenome	121,659	121,700	chromosome 6	10,829,383	10,829,342	100	42	0	0	2.77×10^{-10}
mitogenome	334,689	334,730	chromosome 1	47,946,477	47,946,518	100	42	0	0	2.77×10^{-10}
mitogenome	113,133	113,174	chromosome 1	50,439,555	50,439,514	97.619	42	1	0	1.29×10^{-08}

mitogenome	241,259	241,300	chromosome 2	29,880,672	29,880,631	100	42	0	0	2.77×10^{-10}
mitogenome	154,857	154,898	chromosome 2	29,146,324	29,146,364	97.619	42	0	1	4.63×10^{-08}
mitogenome	238,497	238,538	chromosome 3	18,905,919	18,905,960	100	42	0	0	2.77×10^{-10}
mitogenome	55,268	55,307	chromosome 4	8,782,986	8,782,946	95.122	41	1	1	7.75×10^{-06}
mitogenome	479,886	479,926	chromosome 5	2,830,258	2,830,218	95.122	41	2	0	2.15×10^{-06}
mitogenome	439,053	439,093	chromosome 2	14,929,915	14,929,875	100	41	0	0	9.95×10^{-10}
mitogenome	279,830	279,870	chromosome 3	5,894,775	5,894,814	95.122	41	1	1	7.75×10^{-06}
mitogenome	240,764	240,803	chromosome 6	21,239,604	21,239,565	100	40	0	0	3.58×10^{-09}
mitogenome	53,457	53,496	chromosome 1	275,932	275,971	100	40	0	0	3.58×10^{-09}
mitogenome	390,248	390,287	chromosome 5	23,651,119	23,651,080	100	40	0	0	3.58×10^{-09}
mitogenome	186,174	186,213	chromosome 5	23,651,080	23,651,119	100	40	0	0	3.58×10^{-09}
mitogenome	308,809	308,847	chromosome 5	30,240,689	30,240,728	97.5	40	0	1	5.99×10^{-07}
mitogenome	496,034	496,073	chromosome 2	14,377,625	14,377,664	100	40	0	0	3.58×10^{-09}
mitogenome	117,239	117,278	chromosome 3	2,508,614	2,508,575	100	40	0	0	3.58×10^{-09}
mitogenome	227,982	228,021	chromosome 3	18,382,886	18,382,925	97.5	40	1	0	1.67×10^{-07}
mitogenome	244,603	244,641	chromosome 4	1,615,540	1,615,502	100	39	0	0	1.29×10^{-08}
mitogenome	159,793	159,831	chromosome 4	25,534,951	25,534,989	97.436	39	1	0	5.99×10^{-07}
mitogenome	223,049	223,087	chromosome 6	5,552,670	5,552,708	100	39	0	0	1.29×10^{-08}
mitogenome	127,823	127,861	chromosome 6	6,135,041	6,135,003	100	39	0	0	1.29×10^{-08}
mitogenome	241,264	241,302	chromosome 1	3,879,277	3,879,315	100	39	0	0	1.29×10^{-08}
mitogenome	241,264	241,302	chromosome 1	5,706,515	5,706,553	100	39	0	0	1.29×10^{-08}
mitogenome	107,807	107,845	chromosome 1	21,243,326	21,243,288	97.436	39	1	0	5.99×10^{-07}
mitogenome	390,145	390,183	chromosome 7	6,211,742	6,211,780	100	39	0	0	1.29×10^{-08}
mitogenome	143,836	143,873	chromosome 2	565,439	565,476	97.368	38	1	0	2.15×10^{-06}
mitogenome	465,325	465,362	chromosome 3	7,122,150	7,122,113	100	38	0	0	4.63×10^{-08}
mitogenome	133,047	133,084	chromosome 3	14,251,230	14,251,193	100	38	0	0	4.63×10^{-08}
mitogenome	345,636	345,672	chromosome 4	17,191,908	17,191,872	100	37	0	0	1.67×10^{-07}
mitogenome	163,509	163,545	chromosome 4	17,191,872	17,191,908	100	37	0	0	1.67×10^{-07}
mitogenome	109,907	109,943	chromosome 8	13,157,679	13,157,643	100	37	0	0	1.67×10^{-07}
mitogenome	450,960	450,996	chromosome 6	8,053,310	8,053,274	97.297	37	1	0	7.75×10^{-06}
mitogenome	450,960	450,996	chromosome 6	9,259,989	9,259,953	97.297	37	1	0	7.75×10^{-06}
mitogenome	199,248	199,284	chromosome 1	20,867,752	20,867,788	97.297	37	1	0	7.75×10^{-06}
mitogenome	305,002	305,038	chromosome 1	20,867,752	20,867,788	97.297	37	1	0	7.75×10^{-06}
mitogenome	106,965	107,001	chromosome 1	25,616,483	25,616,447	97.297	37	1	0	7.75×10^{-06}
mitogenome	149,170	149,206	chromosome 1	47,278,816	47,278,852	97.297	37	1	0	7.75×10^{-06}
mitogenome	149,170	149,206	chromosome 1	47,405,529	47,405,565	97.297	37	1	0	7.75×10^{-06}
mitogenome	163,509	163,545	chromosome 7	12,819,835	12,819,799	100	37	0	0	1.67×10^{-07}
mitogenome	345,636	345,672	chromosome 7	12,819,799	12,819,835	100	37	0	0	1.67×10^{-07}

mitogenome	345,636	345,672	chromosome 5	12,891,545	12,891,509	100	37	0	0	$1.67{\times}10^{-07}$
mitogenome	163,509	163,545	chromosome 5	12,891,509	12,891,545	100	37	0	0	$1.67{\times}10^{-07}$
mitogenome	167,685	167,721	chromosome 5	21,050,548	21,050,584	97.297	37	1	0	$7.75{\times}10^{-06}$
mitogenome	442,389	442,425	chromosome 2	11,291,915	11,291,879	97.297	37	1	0	$7.75{\times}10^{-06}$
mitogenome	437,312	437,348	chromosome 3	26,702,030	26,702,066	97.297	37	1	0	$7.75{\times}10^{-06}$
mitogenome	180,929	180,964	chromosome 4	1,848,400	1,848,435	100	36	0	0	$5.99{\times}10^{-07}$
mitogenome	144,648	144,683	chromosome 4	25,534,985	25,534,950	100	36	0	0	$5.99{\times}10^{-07}$
mitogenome	3,592	3,627	chromosome 4	25,534,985	25,534,950	100	36	0	0	$5.99{\times}10^{-07}$
mitogenome	331,136	331,171	chromosome 1	33,261,114	33,261,149	100	36	0	0	$5.99{\times}10^{-07}$
mitogenome	119,636	119,671	chromosome 1	46,428,520	46,428,555	100	36	0	0	$5.99{\times}10^{-07}$
mitogenome	331,136	331,171	chromosome 7	7,698,962	7,698,997	100	36	0	0	$5.99{\times}10^{-07}$
mitogenome	428,853	428,888	chromosome 7	7,699,136	7,699,171	100	36	0	0	$5.99{\times}10^{-07}$
mitogenome	109,418	109,453	chromosome 2	15,991,164	15,991,199	100	36	0	0	$5.99{\times}10^{-07}$
mitogenome	284,170	284,205	chromosome 2	25,763,294	25,763,329	100	36	0	0	$5.99{\times}10^{-07}$
mitogenome	284,170	284,205	chromosome 2	27,409,316	27,409,351	100	36	0	0	$5.99{\times}10^{-07}$
mitogenome	87,190	87,225	chromosome 3	32,074,208	32,074,243	100	36	0	0	$5.99{\times}10^{-07}$
mitogenome	428,914	428,948	chromosome 4	13,472,833	13,472,867	100	35	0	0	$2.15{\times}10^{-06}$
mitogenome	371,359	371,393	chromosome 4	25,534,985	25,534,951	100	35	0	0	$2.15{\times}10^{-06}$
mitogenome	343,085	343,119	chromosome 4	25,534,951	25,534,985	100	35	0	0	$2.15{\times}10^{-06}$
mitogenome	456,224	456,258	chromosome 4	25,534,951	25,534,985	100	35	0	0	$2.15{\times}10^{-06}$
mitogenome	460,965	460,999	chromosome 4	25,534,951	25,534,985	100	35	0	0	$2.15{\times}10^{-06}$
mitogenome	316,756	316,790	chromosome 4	29,813,659	29,813,625	100	35	0	0	$2.15{\times}10^{-06}$
mitogenome	316,813	316,847	chromosome 4	29,813,716	29,813,682	100	35	0	0	$2.15{\times}10^{-06}$
mitogenome	261,222	261,256	chromosome 6	9,987,838	9,987,872	100	35	0	0	$2.15{\times}10^{-06}$
mitogenome	261,222	261,256	chromosome 6	9,989,336	9,989,370	100	35	0	0	$2.15{\times}10^{-06}$
mitogenome	119,637	119,671	chromosome 1	46,436,339	46,436,373	100	35	0	0	$2.15{\times}10^{-06}$
mitogenome	176,743	176,777	chromosome 2	2,091,668	2,091,702	100	35	0	0	$2.15{\times}10^{-06}$
mitogenome	396,441	396,475	chromosome 3	30,778,170	30,778,204	100	35	0	0	$2.15{\times}10^{-06}$
mitogenome	391,847	391,880	chromosome 6	3,381,898	3,381,865	100	34	0	0	$7.75{\times}10^{-06}$
mitogenome	208,948	208,981	chromosome 6	21,239,534	21,239,567	100	34	0	0	$7.75{\times}10^{-06}$
mitogenome	391,117	391,150	chromosome 1	25,065,061	25,065,094	100	34	0	0	$7.75{\times}10^{-06}$
mitogenome	57,273	57,306	chromosome 7	9,431,242	9,431,209	100	34	0	0	$7.75{\times}10^{-06}$
mitogenome	57,273	57,306	chromosome 7	10,023,467	10,023,500	100	34	0	0	$7.75{\times}10^{-06}$
mitogenome	268,309	268,342	chromosome 5	20,158,144	20,158,111	100	34	0	0	$7.75{\times}10^{-06}$
mitogenome	113,133	113,166	chromosome 5	23,651,113	23,651,080	100	34	0	0	$7.75{\times}10^{-06}$
mitogenome	379,099	379,132	chromosome 3	662,160	662,193	100	34	0	0	$7.75{\times}10^{-06}$

Table S3. The dispersed repeats identified in the mitogenome of *P. salicina*.

ID	Repeat Types	Repeat Length（bp）	Repeat Units I Start	Repeat Units I End	Repeat Units II Start	Repeat Units II End	Repeat Interval	E–Value
1	F	691	199,012	199,702	304,766	305,456	0	0.00×10^{1}
2	P	557	163,079	163,635	345,544	346,100	–1	0.00×10^{1}
3	P	529	156,449	156,977	409,835	410,363	–2	0.00×10^{1}
4	P	526	163,110	163,635	345,544	346,069	0	0.00×10^{1}
5	P	511	200,802	201,312	418,999	419,509	0	0.00×10^{1}
6	P	393	164,677	165,069	345,034	345,426	0	1.78×10^{-226}
7	F	385	361,587	361,971	501,440	501,824	0	1.17×10^{-221}
8	P	326	274,169	274,494	348,698	349,023	–1	3.80×10^{-183}
9	P	329	280,897	281,225	358,066	358,394	–2	2.95×10^{-182}
10	P	320	280,906	281,225	358,066	358,385	–1	1.53×10^{-179}
11	P	311	274,184	274,494	348,698	349,008	0	4.17×10^{-177}
12	P	299	280,927	281,225	358,066	358,364	0	7.00×10^{-170}
13	P	294	123,382	123,675	154,570	154,863	0	7.17×10^{-167}
14	F	248	156,951	157,198	164,552	164,799	–1	2.64×10^{-136}
15	F	195	215,707	215,901	303,435	303,629	0	2.88×10^{-107}
16	F	175	290,958	291,132	362,215	362,389	0	3.17×10^{-95}
17	P	165	409,141	409,305	478,838	479,002	–2	4.04×10^{-84}
18	P	162	7,147	7,308	460,113	460,274	–3	3.99×10^{-80}
19	P	146	156,926	157,071	409,741	409,886	–2	8.69×10^{-73}
20	P	138	409,168	409,305	478,838	478,975	–1	2.48×10^{-70}
21	P	144	201,905	202,048	358,588	358,731	–3	1.92×10^{-69}
22	P	141	277,606	277,746	375,286	375,426	–3	1.15×10^{-67}
23	P	129	409,177	409,305	478,838	478,966	0	1.57×10^{-67}
24	F	133	52,874	53,006	359,248	359,380	–1	2.44×10^{-67}
25	P	128	155,555	155,682	244,255	244,382	0	6.27×10^{-67}
26	P	132	52,958	53,089	197,093	197,224	–1	9.70×10^{-67}
27	P	135	17,358	17,492	274,689	274,823	–2	3.11×10^{-66}
28	P	129	52,735	52,863	302,485	302,613	–1	6.07×10^{-65}
29	F	132	164,676	164,807	317,071	317,202	–3	2.48×10^{-62}
30	P	131	202,112	202,242	358,385	358,515	–3	9.68×10^{-62}
31	P	131	317,072	317,202	345,296	345,426	–3	9.68×10^{-62}
32	P	123	157,076	157,198	345,304	345,426	–1	2.37×10^{-61}
33	P	118	52,746	52,863	302,485	302,602	0	6.57×10^{-61}
34	F	124	157,075	157,198	317,071	317,194	–2	1.10×10^{-59}
35	P	121	164,552	164,672	409,741	409,861	–2	6.71×10^{-58}
36	P	121	201,928	202,048	358,588	358,708	–2	6.71×10^{-58}
37	F	112	164,661	164,772	495,567	495,678	0	2.69×10^{-57}
38	P	111	89,334	89,444	343,114	343,224	0	1.08×10^{-56}
39	P	118	7,191	7,308	460,113	460,230	–2	4.08×10^{-56}
40	P	116	436,971	437,086	496,403	496,518	–2	6.31×10^{-55}
41	F	112	157,060	157,171	495,567	495,678	–1	9.05×10^{-55}
42	P	107	315,038	315,144	367,810	367,916	0	2.76×10^{-54}
43	F	115	23,698	23,812	403,521	403,635	–3	2.80×10^{-52}
44	P	114	202,076	202,189	358,438	358,551	–3	1.09×10^{-51}
45	F	99	199,699	199,797	305,454	305,552	0	1.81×10^{-49}
46	P	110	52,400	52,509	201,369	201,478	–3	2.51×10^{-49}

47	P	102	202,141	202,242	358,385	358,486	–1	8.64×10^{-49}
48	P	96	345,331	345,426	495,583	495,678	0	1.16×10^{-47}
49	F	107	23,762	23,868	403,585	403,691	–3	1.48×10^{-47}
50	F	100	164,708	164,807	317,103	317,202	–2	2.01×10^{-45}
51	P	100	317,103	317,202	345,296	345,395	–2	2.01×10^{-45}
52	F	92	157,107	157,198	164,708	164,799	0	2.96×10^{-45}
53	P	92	157,107	157,198	345,304	345,395	0	2.96×10^{-45}
54	P	93	156,979	157,071	409,741	409,833	–1	2.06×10^{-43}
55	P	93	164,580	164,672	409,741	409,833	–1	2.06×10^{-43}
56	P	88	53,002	53,089	197,093	197,180	0	7.58×10^{-43}
57	F	87	297,050	297,136	338,917	339,003	0	3.03×10^{-42}
58	F	87	386,842	386,928	391,154	391,240	0	3.03×10^{-42}
59	F	97	317,071	317,167	495,582	495,678	–3	1.15×10^{-41}
60	F	97	459,928	460,024	495,611	495,707	–3	1.15×10^{-41}
61	P	96	122,348	122,443	460,710	460,805	–3	4.46×10^{-41}
62	P	95	281,755	281,849	447,283	447,377	–3	1.73×10^{-40}
63	P	84	276,727	276,810	334,368	334,451	0	1.94×10^{-40}
64	F	92	691	782	419,694	419,785	–3	1.00×10^{-38}
65	P	81	73,971	74,051	353,216	353,296	0	1.24×10^{-38}
66	P	90	163,660	163,749	345,430	345,519	–3	1.50×10^{-37}
67	F	83	164,725	164,807	317,120	317,202	–1	1.93×10^{-37}
68	P	83	317,120	317,202	345,296	345,378	–1	1.93×10^{-37}
69	F	89	243,258	243,346	360,621	360,709	–3	5.81×10^{-37}
70	P	78	201,971	202,048	358,588	358,665	0	7.95×10^{-37}
71	F	84	699	782	419,702	419,785	–2	6.09×10^{-36}
72	F	84	23,785	23,868	403,608	403,691	–2	6.09×10^{-36}
73	P	87	163,648	163,734	345,445	345,531	–3	8.68×10^{-36}
74	F	80	189,272	189,351	273,347	273,426	–1	1.19×10^{-35}
75	P	77	122,367	122,443	460,710	460,786	–1	7.34×10^{-34}
76	F	72	53,454	53,525	122,677	122,748	0	3.26×10^{-33}
77	F	79	459,906	459,984	479,028	479,106	–2	5.51×10^{-33}
78	F	75	157,124	157,198	317,120	317,194	–1	1.14×10^{-32}
79	F	71	16,825	16,895	52,609	52,679	0	1.30×10^{-32}
80	P	71	114,047	114,117	317,239	317,309	0	1.30×10^{-32}
81	F	71	147,157	147,227	430,184	430,254	0	1.30×10^{-32}
82	F	71	164,683	164,753	479,028	479,098	0	1.30×10^{-32}
83	P	71	345,350	345,420	479,028	479,098	0	1.30×10^{-32}
84	F	71	479,028	479,098	495,589	495,659	0	1.30×10^{-32}
85	P	78	345,350	345,427	459,899	459,976	–2	2.15×10^{-32}
86	F	74	94,263	94,336	272,442	272,515	–1	4.52×10^{-32}
87	F	77	164,677	164,753	459,900	459,976	–2	8.37×10^{-32}
88	F	77	459,900	459,976	495,583	495,659	–2	8.37×10^{-32}
89	F	80	63,191	63,270	219,015	219,094	–3	1.10×10^{-31}
90	F	73	241,463	241,535	394,695	394,767	–1	1.78×10^{-31}
91	F	68	62,980	63,047	460,816	460,883	0	8.33×10^{-31}
92	P	75	163,675	163,749	345,430	345,504	–2	1.27×10^{-30}
93	F	75	459,950	460,024	495,633	495,707	–2	1.27×10^{-30}
94	P	71	17,422	17,492	274,689	274,759	–1	2.77×10^{-30}
95	F	71	157,082	157,152	479,028	479,098	–1	2.77×10^{-30}
96	P	71	165,221	165,291	218,900	218,970	–1	2.77×10^{-30}
97	F	77	157,076	157,152	459,900	459,976	–3	6.28×10^{-30}
98	F	77	459,928	460,004	479,050	479,126	–3	6.28×10^{-30}
99	F	76	477,524	477,599	499,644	499,719	–3	2.41×10^{-29}

100	F	65	157,107	157,171	495,614	495,678	0	5.33×10^{-29}
101	P	65	186,151	186,215	241,468	241,532	0	5.33×10^{-29}
102	F	65	241,464	241,528	437,593	437,657	0	5.33×10^{-29}
103	F	65	394,696	394,760	437,593	437,657	0	5.33×10^{-29}
104	F	71	317,078	317,148	479,028	479,098	–2	2.91×10^{-28}
105	F	63	433,478	433,540	463,193	463,255	0	8.53×10^{-28}
106	P	66	201,813	201,878	358,758	358,823	–1	2.64×10^{-27}
107	P	61	186,155	186,215	394,700	394,760	0	1.37×10^{-26}
108	P	61	186,155	186,215	437,597	437,657	0	1.37×10^{-26}
109	F	64	7,638	7,701	390,247	390,310	–1	4.10×10^{-26}
110	F	64	274,262	274,325	478,563	478,626	–1	4.10×10^{-26}
111	P	64	348,867	348,930	478,563	478,626	–1	4.10×10^{-26}
112	P	60	165,232	165,291	218,900	218,959	0	5.46×10^{-26}
113	F	70	9,462	9,531	241,136	241,205	–3	7.70×10^{-26}
114	F	63	96,346	96,408	356,018	356,080	–1	1.61×10^{-25}
115	P	59	186,160	186,218	390,241	390,299	0	2.18×10^{-25}
116	F	58	442,232	442,289	445,485	445,542	0	8.74×10^{-25}
117	F	65	317,103	317,167	495,614	495,678	–2	9.98×10^{-25}
118	F	68	164,705	164,772	459,928	459,995	–3	1.13×10^{-24}
119	P	68	345,331	345,398	459,928	459,995	–3	1.13×10^{-24}
120	P	64	356,018	356,081	449,684	449,747	–2	3.87×10^{-24}
121	P	60	163,690	163,749	345,430	345,489	–1	9.83×10^{-24}
122	F	56	241,468	241,523	390,244	390,299	0	1.40×10^{-23}
123	F	56	390,244	390,299	394,700	394,755	0	1.40×10^{-23}
124	F	56	390,244	390,299	437,597	437,652	0	1.40×10^{-23}
125	F	59	177,401	177,459	390,243	390,301	–1	3.87×10^{-23}
126	F	62	196,296	196,357	280,361	280,422	–2	5.81×10^{-23}
127	F	65	157,107	157,171	459,931	459,995	–3	6.29×10^{-23}
128	F	58	7,638	7,695	223,764	223,821	–1	1.52×10^{-22}
129	F	58	177,396	177,453	394,694	394,751	–1	1.52×10^{-22}
130	P	58	437,120	437,177	496,318	496,375	–1	1.52×10^{-22}
131	F	54	23,815	23,868	403,638	403,691	0	2.24×10^{-22}
132	F	61	63,237	63,297	219,061	219,121	–2	2.25×10^{-22}
133	P	61	114,111	114,171	459,981	460,041	–2	2.25×10^{-22}
134	F	61	223,761	223,821	390,244	390,304	–2	2.25×10^{-22}
135	F	57	177,397	177,453	241,463	241,519	–1	5.98×10^{-22}
136	F	57	223,758	223,814	241,465	241,521	–1	5.98×10^{-22}
137	F	57	223,758	223,814	394,697	394,753	–1	5.98×10^{-22}
138	F	57	223,758	223,814	437,594	437,650	–1	5.98×10^{-22}
139	F	53	147,158	147,210	449,351	449,403	0	8.95×10^{-22}
140	P	53	177,401	177,453	186,164	186,216	0	8.95×10^{-22}
141	P	53	348,878	348,930	478,563	478,615	0	8.95×10^{-22}
142	F	53	430,185	430,237	449,351	449,403	0	8.95×10^{-22}
143	F	63	728	790	419,731	419,793	–3	9.15×10^{-22}
144	P	63	6,153	6,215	274,734	274,796	–3	9.15×10^{-22}
145	P	63	96,346	96,408	449,685	449,747	–3	9.15×10^{-22}
146	P	62	281,805	281,866	447,266	447,327	–3	3.49×10^{-21}
147	P	52	122,015	122,066	429,234	429,285	0	3.58×10^{-21}
148	F	52	177,402	177,453	437,597	437,648	0	3.58×10^{-21}
149	P	52	202,191	202,242	358,385	358,436	0	3.58×10^{-21}
150	F	55	67,972	68,026	156,412	156,466	–1	9.23×10^{-21}
151	F	55	183,575	183,629	329,274	329,328	–1	9.23×10^{-21}
152	P	58	62,908	62,965	430,221	430,278	–2	1.30×10^{-20}

153	P	51	219,173	219,223	494,719	494,769	0	1.43×10^{-20}
154	P	54	186,162	186,215	223,761	223,814	–1	3.62×10^{-20}
155	F	50	94,287	94,336	272,466	272,515	0	5.73×10^{-20}
156	P	53	7,638	7,690	186,160	186,212	–1	1.42×10^{-19}
157	F	53	7,638	7,690	241,471	241,523	–1	1.42×10^{-19}
158	F	53	7,638	7,690	394,703	394,755	–1	1.42×10^{-19}
159	F	53	7,638	7,690	437,600	437,652	–1	1.42×10^{-19}
160	P	53	114,119	114,171	459,981	460,033	–1	1.42×10^{-19}
161	F	49	65,596	65,644	430,640	430,688	0	2.29×10^{-19}
162	F	49	240,043	240,091	496,033	496,081	0	2.29×10^{-19}
163	F	52	177,402	177,453	223,761	223,812	–1	5.58×10^{-19}
164	P	58	274,125	274,182	349,010	349,067	–3	7.28×10^{-19}
165	F	55	7,638	7,692	177,405	177,459	–2	7.47×10^{-19}
166	P	55	22,974	23,028	247,508	247,562	–2	7.47×10^{-19}
167	F	55	459,950	460,004	479,072	479,126	–2	7.47×10^{-19}
168	F	48	20,714	20,761	449,116	449,163	0	9.16×10^{-19}
169	P	48	53,047	53,094	460,063	460,110	0	9.16×10^{-19}
170	F	48	64,145	64,192	276,049	276,096	0	9.16×10^{-19}
171	F	54	422,776	422,829	434,374	434,427	–2	2.88×10^{-18}
172	F	47	477,466	477,512	499,596	499,642	0	3.66×10^{-18}
173	P	50	345,378	345,427	459,899	459,948	–1	8.59×10^{-18}
174	F	46	157,107	157,152	479,053	479,098	0	1.47×10^{-17}
175	F	46	459,979	460,024	495,662	495,707	0	1.47×10^{-17}
176	P	49	73,923	73,971	353,481	353,529	–1	3.37×10^{-17}
177	F	49	113,132	113,180	177,411	177,459	–1	3.37×10^{-17}
178	P	48	16,986	17,033	276,779	276,826	–1	1.32×10^{-16}
179	F	48	317,120	317,167	495,631	495,678	–1	1.32×10^{-16}
180	F	54	317,103	317,156	479,053	479,106	–3	1.50×10^{-16}
181	F	51	17,045	17,095	333,915	333,965	–2	1.64×10^{-16}
182	P	44	88,877	88,920	503,650	503,693	0	2.35×10^{-16}
183	F	44	260,063	260,106	460,854	460,897	0	2.35×10^{-16}
184	F	47	53,043	53,089	359,410	359,456	–1	5.17×10^{-16}
185	F	47	113,178	113,224	240,266	240,312	–1	5.17×10^{-16}
186	P	47	197,093	197,139	359,410	359,456	–1	5.17×10^{-16}
187	P	47	409,510	409,556	495,271	495,317	–1	5.17×10^{-16}
188	F	53	243,408	243,460	360,767	360,819	–3	5.66×10^{-16}
189	P	50	239,749	239,798	272,249	272,298	–2	6.31×10^{-16}
190	F	43	4,148	4,190	352,804	352,846	0	9.38×10^{-16}
191	P	43	113,132	113,174	186,164	186,206	0	9.38×10^{-16}
192	F	43	113,132	113,174	241,477	241,519	0	9.38×10^{-16}
193	F	43	113,132	113,174	390,253	390,295	0	9.38×10^{-16}
194	F	43	113,132	113,174	394,709	394,751	0	9.38×10^{-16}
195	F	43	113,132	113,174	437,606	437,648	0	9.38×10^{-16}
196	P	43	122,401	122,443	460,710	460,752	0	9.38×10^{-16}
197	F	43	197,093	197,135	460,068	460,110	0	9.38×10^{-16}
198	P	46	7,263	7,308	460,113	460,158	–1	2.02×10^{-15}
199	F	52	135,764	135,815	205,214	205,265	–3	2.14×10^{-15}
200	P	52	163,637	163,688	345,491	345,542	–3	2.14×10^{-15}
201	P	49	197,176	197,224	359,332	359,380	–2	2.42×10^{-15}
202	F	42	293,758	293,799	293,779	293,820	0	3.75×10^{-15}
203	P	45	62,921	62,965	430,221	430,265	–1	7.92×10^{-15}
204	P	48	167,512	167,559	215,918	215,965	–2	9.30×10^{-15}
205	P	41	62,971	63,011	449,341	449,381	0	1.50×10^{-14}

206	F	41	88,880	88,920	374,812	374,852	0	1.50×10^{-14}
207	F	41	164,767	164,807	317,162	317,202	0	1.50×10^{-14}
208	F	41	199,470	199,510	272,615	272,655	0	1.50×10^{-14}
209	F	41	272,615	272,655	305,224	305,264	0	1.50×10^{-14}
210	P	41	317,162	317,202	345,296	345,336	0	1.50×10^{-14}
211	P	41	374,812	374,852	503,650	503,690	0	1.50×10^{-14}
212	F	41	456,217	456,257	460,958	460,998	0	1.50×10^{-14}
213	F	50	6,153	6,202	17,385	17,434	–3	3.03×10^{-14}
214	F	50	281,927	281,976	329,787	329,836	–3	3.03×10^{-14}
215	F	44	88,064	88,107	116,462	116,505	–1	3.10×10^{-14}
216	P	44	114,128	114,171	495,664	495,707	–1	3.10×10^{-14}
217	F	47	317,072	317,118	459,900	459,946	–2	3.57×10^{-14}
218	P	47	359,414	359,460	460,064	460,110	–2	3.57×10^{-14}
219	P	40	230,219	230,258	420,018	420,057	0	6.00×10^{-14}
220	F	43	7,644	7,686	113,132	113,174	–1	1.21×10^{-13}
221	F	43	113,132	113,174	223,770	223,812	–1	1.21×10^{-13}
222	F	46	157,126	157,171	459,950	459,995	–2	1.37×10^{-13}
223	F	46	164,727	164,772	459,950	459,995	–2	1.37×10^{-13}
224	F	46	317,103	317,148	459,931	459,976	–2	1.37×10^{-13}
225	P	46	348,345	348,390	386,574	386,619	–2	1.37×10^{-13}
226	F	39	58,985	59,023	390,144	390,182	0	2.40×10^{-13}
227	P	39	114,133	114,171	459,981	460,019	0	2.40×10^{-13}
228	F	39	156,176	156,214	187,301	187,339	0	2.40×10^{-13}
229	F	39	196,319	196,357	280,384	280,422	0	2.40×10^{-13}
230	F	39	373,809	373,847	442,100	442,138	0	2.40×10^{-13}
231	P	48	62,727	62,774	240,976	241,023	–3	4.28×10^{-13}
232	F	48	76,482	76,529	198,326	198,373	–3	4.28×10^{-13}
233	F	45	96,376	96,420	306,971	307,015	–2	5.22×10^{-13}
234	F	38	3,590	3,627	144,646	144,683	0	9.61×10^{-13}
235	F	38	4,747	4,784	144,489	144,526	0	9.61×10^{-13}
236	P	38	82,872	82,909	424,404	424,441	0	9.61×10^{-13}
237	F	38	159,790	159,827	460,962	460,999	0	9.61×10^{-13}
238	F	38	189,624	189,661	273,458	273,495	0	9.61×10^{-13}
239	F	38	343,084	343,121	460,964	461,001	0	9.61×10^{-13}
240	P	47	236,849	236,895	473,771	473,817	–3	1.60×10^{-12}
241	P	47	334,361	334,407	388,640	388,686	–3	1.60×10^{-12}
242	P	41	16,754	16,794	163,816	163,856	–1	1.85×10^{-12}
243	F	41	23,185	23,225	447,234	447,274	–1	1.85×10^{-12}
244	P	41	129,270	129,310	228,184	228,224	–1	1.85×10^{-12}
245	F	41	291,614	291,654	460,964	461,004	–1	1.85×10^{-12}
246	F	44	302,360	302,403	464,543	464,586	–2	2.00×10^{-12}
247	F	37	7,665	7,701	390,274	390,310	0	3.84×10^{-12}
248	P	37	144,645	144,681	159,792	159,828	0	3.84×10^{-12}
249	F	37	159,790	159,826	456,221	456,257	0	3.84×10^{-12}
250	P	37	437,085	437,121	496,369	496,405	0	3.84×10^{-12}
251	F	46	9,511	9,556	241,185	241,230	–3	6.01×10^{-12}
252	P	46	26,182	26,227	334,185	334,230	–3	6.01×10^{-12}
253	F	46	317,122	317,167	459,950	459,995	–3	6.01×10^{-12}
254	F	40	17,056	17,095	333,926	333,965	–1	7.21×10^{-12}
255	F	43	243,393	243,435	360,752	360,794	–2	7.62×10^{-12}
256	P	43	277,704	277,746	375,286	375,328	–2	7.62×10^{-12}
257	P	36	3,590	3,625	159,792	159,827	0	1.54×10^{-11}
258	P	36	3,590	3,625	343,084	343,119	0	1.54×10^{-11}

259	P	36	3,590	3,625	460,964	460,999	0	1.54×10^{-11}
260	F	36	3,591	3,626	223,364	223,399	0	1.54×10^{-11}
261	F	36	98,919	98,954	277,460	277,495	0	1.54×10^{-11}
262	P	36	144,646	144,681	343,084	343,119	0	1.54×10^{-11}
263	P	36	144,646	144,681	460,964	460,999	0	1.54×10^{-11}
264	F	36	144,647	144,682	223,364	223,399	0	1.54×10^{-11}
265	F	36	159,792	159,827	343,084	343,119	0	1.54×10^{-11}
266	P	36	201,843	201,878	358,758	358,793	0	1.54×10^{-11}
267	F	36	277,459	277,494	453,483	453,518	0	1.54×10^{-11}
268	P	36	343,083	343,118	371,358	371,393	0	1.54×10^{-11}
269	P	36	371,357	371,392	456,223	456,258	0	1.54×10^{-11}
270	P	45	129,258	129,302	287,641	287,685	–3	2.25×10^{-11}
271	F	39	3,062	3,100	309,783	309,821	–1	2.81×10^{-11}
272	P	39	60,056	60,094	98,917	98,955	–1	2.81×10^{-11}
273	F	42	243,305	243,346	360,668	360,709	–2	2.91×10^{-11}
274	F	35	748	782	419,751	419,785	0	6.15×10^{-11}
275	F	35	3,591	3,625	371,358	371,392	0	6.15×10^{-11}
276	P	35	3,591	3,625	456,223	456,257	0	6.15×10^{-11}
277	P	35	60,699	60,733	122,254	122,288	0	6.15×10^{-11}
278	F	35	74,014	74,048	291,615	291,649	0	6.15×10^{-11}
279	F	35	98,919	98,953	453,484	453,518	0	6.15×10^{-11}
280	F	35	122,255	122,289	309,784	309,818	0	6.15×10^{-11}
281	F	35	129,030	129,064	287,641	287,675	0	6.15×10^{-11}
282	F	35	144,647	144,681	371,358	371,392	0	6.15×10^{-11}
283	P	35	144,647	144,681	456,223	456,257	0	6.15×10^{-11}
284	P	35	159,792	159,826	223,364	223,398	0	6.15×10^{-11}
285	P	35	159,792	159,826	371,358	371,392	0	6.15×10^{-11}
286	P	35	223,364	223,398	343,084	343,118	0	6.15×10^{-11}
287	F	35	223,364	223,398	371,358	371,392	0	6.15×10^{-11}
288	P	35	223,364	223,398	456,223	456,257	0	6.15×10^{-11}
289	P	35	223,364	223,398	460,964	460,998	0	6.15×10^{-11}
290	F	35	263,861	263,895	475,397	475,431	0	6.15×10^{-11}
291	P	35	287,640	287,674	449,684	449,718	0	6.15×10^{-11}
292	F	35	291,307	291,341	291,613	291,647	0	6.15×10^{-11}
293	P	35	291,615	291,649	353,219	353,253	0	6.15×10^{-11}
294	F	35	316,755	316,789	316,812	316,846	0	6.15×10^{-11}
295	F	35	343,084	343,118	456,223	456,257	0	6.15×10^{-11}
296	P	35	356,017	356,051	393,129	393,163	0	6.15×10^{-11}
297	P	35	371,358	371,392	460,964	460,998	0	6.15×10^{-11}
298	P	38	22,991	23,028	247,508	247,545	–1	1.10×10^{-10}
299	P	38	53,725	53,762	464,410	464,447	–1	1.10×10^{-10}
300	F	38	129,265	129,302	449,680	449,717	–1	1.10×10^{-10}
301	P	38	239,761	239,798	272,249	272,286	–1	1.10×10^{-10}
302	F	38	287,639	287,676	356,046	356,083	–1	1.10×10^{-10}
303	F	38	291,614	291,651	343,084	343,121	–1	1.10×10^{-10}
304	P	41	63,006	63,046	279,829	279,869	–2	1.11×10^{-10}
305	F	41	74,008	74,048	290,960	291,000	–2	1.11×10^{-10}
306	F	41	74,008	74,048	362,217	362,257	–2	1.11×10^{-10}
307	P	41	279,829	279,869	460,842	460,882	–2	1.11×10^{-10}
308	P	41	290,960	291,000	353,219	353,259	–2	1.11×10^{-10}
309	P	41	334,679	334,719	460,529	460,569	–2	1.11×10^{-10}
310	P	41	353,219	353,259	362,217	362,257	–2	1.11×10^{-10}
311	P	34	17,459	17,492	274,689	274,722	0	2.46×10^{-10}

312	P	34	43,209	43,242	106,552	106,585	0	2.46×10^{-10}
313	F	34	46,151	46,184	168,590	168,623	0	2.46×10^{-10}
314	P	34	60,699	60,732	309,784	309,817	0	2.46×10^{-10}
315	F	34	63,347	63,380	327,185	327,218	0	2.46×10^{-10}
316	F	34	88,074	88,107	116,472	116,505	0	2.46×10^{-10}
317	F	34	88,887	88,920	443,384	443,417	0	2.46×10^{-10}
318	P	34	96,346	96,379	393,129	393,162	0	2.46×10^{-10}
319	P	34	103,343	103,376	401,925	401,958	0	2.46×10^{-10}
320	P	34	113,107	113,140	429,140	429,173	0	2.46×10^{-10}
321	P	34	113,191	113,224	129,029	129,062	0	2.46×10^{-10}
322	F	34	113,191	113,224	129,270	129,303	0	2.46×10^{-10}
323	P	34	113,191	113,224	228,191	228,224	0	2.46×10^{-10}
324	F	34	121,974	122,007	449,217	449,250	0	2.46×10^{-10}
325	P	34	129,029	129,062	129,270	129,303	0	2.46×10^{-10}
326	F	34	129,029	129,062	228,191	228,224	0	2.46×10^{-10}
327	P	34	129,030	129,063	449,684	449,717	0	2.46×10^{-10}
328	P	34	212,118	212,151	236,838	236,871	0	2.46×10^{-10}
329	F	34	240,309	240,342	442,071	442,104	0	2.46×10^{-10}
330	F	34	374,819	374,852	443,384	443,417	0	2.46×10^{-10}
331	P	34	443,384	443,417	503,650	503,683	0	2.46×10^{-10}
332	P	43	84,710	84,752	294,840	294,882	–3	3.13×10^{-10}
333	P	43	269,784	269,826	489,641	489,683	–3	3.13×10^{-10}
334	F	40	340,014	340,053	372,298	372,337	–2	4.22×10^{-10}
335	F	40	504,704	504,743	504,743	504,782	–2	4.22×10^{-10}
336	P	37	3,590	3,626	291,613	291,649	–1	4.27×10^{-10}
337	P	37	44,148	44,184	264,415	264,451	–1	4.27×10^{-10}
338	F	37	111,738	111,774	430,279	430,315	–1	4.27×10^{-10}
339	P	37	144,646	144,682	291,613	291,649	–1	4.27×10^{-10}
340	F	37	290,966	291,002	291,615	291,651	–1	4.27×10^{-10}
341	F	37	291,615	291,651	362,223	362,259	–1	4.27×10^{-10}
342	F	37	408,902	408,938	447,347	447,383	–1	4.27×10^{-10}
343	P	37	435,835	435,871	442,388	442,424	–1	4.27×10^{-10}
344	P	33	3,588	3,620	74,018	74,050	0	9.84×10^{-10}
345	F	33	3,588	3,620	353,217	353,249	0	9.84×10^{-10}
346	F	33	23,864	23,896	403,694	403,726	0	9.84×10^{-10}
347	P	33	59,946	59,978	60,700	60,732	0	9.84×10^{-10}
348	F	33	59,946	59,978	122,255	122,287	0	9.84×10^{-10}
349	F	33	59,946	59,978	309,784	309,816	0	9.84×10^{-10}
350	F	33	74,014	74,046	291,309	291,341	0	9.84×10^{-10}
351	P	33	113,191	113,223	287,641	287,673	0	9.84×10^{-10}
352	F	33	113,191	113,223	449,685	449,717	0	9.84×10^{-10}
353	P	33	141,964	141,996	494,438	494,470	0	9.84×10^{-10}
354	F	33	157,166	157,198	317,162	317,194	0	9.84×10^{-10}
355	R	33	177,389	177,421	177,389	177,421	0	9.84×10^{-10}
356	F	33	202,311	202,343	433,659	433,691	0	9.84×10^{-10}
357	F	33	228,192	228,224	287,641	287,673	0	9.84×10^{-10}
358	P	33	228,192	228,224	449,685	449,717	0	9.84×10^{-10}
359	P	33	291,309	291,341	353,221	353,253	0	9.84×10^{-10}
360	F	33	306,971	307,003	356,048	356,080	0	9.84×10^{-10}
361	P	42	52,475	52,516	201,362	201,403	–3	1.16×10^{-09}
362	F	42	52,982	53,023	359,356	359,397	–3	1.16×10^{-09}
363	P	42	53,949	53,990	464,172	464,213	–3	1.16×10^{-09}
364	P	42	239,717	239,758	266,667	266,708	–3	1.16×10^{-09}

365	P	39	3,590	3,628	177,809	177,847	–2	1.60×10^{-09}
366	P	39	17,002	17,040	388,641	388,679	–2	1.60×10^{-09}
367	F	39	59,794	59,832	59,795	59,833	–2	1.60×10^{-09}
368	P	39	315,137	315,175	367,751	367,789	–2	1.60×10^{-09}
369	P	39	456,256	456,294	506,195	506,233	–2	1.60×10^{-09}
370	P	36	60,057	60,092	277,460	277,495	–1	1.66×10^{-09}
371	F	36	63,262	63,297	219,086	219,121	–1	1.66×10^{-09}
372	F	36	95,804	95,839	97,741	97,776	–1	1.66×10^{-09}
373	F	36	101,706	101,741	372,053	372,088	–1	1.66×10^{-09}
374	P	36	114,116	114,151	317,203	317,238	–1	1.66×10^{-09}
375	P	36	129,268	129,303	306,970	307,005	–1	1.66×10^{-09}
376	F	36	159,792	159,827	291,614	291,649	–1	1.66×10^{-09}
377	F	36	177,813	177,848	343,085	343,120	–1	1.66×10^{-09}
378	F	36	177,813	177,848	460,965	461,000	–1	1.66×10^{-09}
379	P	36	223,364	223,399	291,613	291,648	–1	1.66×10^{-09}
380	P	36	437,051	437,086	496,403	496,438	–1	1.66×10^{-09}
381	P	32	122,316	122,347	460,803	460,834	0	3.94×10^{-09}
382	F	32	304,732	304,763	387,517	387,548	0	3.94×10^{-09}
383	P	32	449,341	449,372	460,816	460,847	0	3.94×10^{-09}
384	F	41	14,677	14,717	391,242	391,282	–3	4.32×10^{-09}
385	P	41	52,484	52,524	201,354	201,394	–3	4.32×10^{-09}
386	F	41	147,193	147,233	371,522	371,562	–3	4.32×10^{-09}
387	F	41	290,962	291,002	460,961	461,001	–3	4.32×10^{-09}
388	F	41	362,219	362,259	460,961	461,001	–3	4.32×10^{-09}
389	F	38	76,492	76,529	198,336	198,373	–2	6.08×10^{-09}
390	F	38	96,325	96,362	306,914	306,951	–2	6.08×10^{-09}
391	F	38	135,789	135,826	205,239	205,276	–2	6.08×10^{-09}
392	F	38	196,895	196,932	198,086	198,123	–2	6.08×10^{-09}
393	P	38	370,173	370,210	421,383	421,420	–2	6.08×10^{-09}
394	P	38	433,875	433,912	449,626	449,663	–2	6.08×10^{-09}
395	F	35	3,063	3,097	122,255	122,289	–1	6.46×10^{-09}
396	P	35	3,592	3,626	291,307	291,341	–1	6.46×10^{-09}
397	P	35	60,058	60,092	453,484	453,518	–1	6.46×10^{-09}
398	F	35	102,285	102,319	418,930	418,964	–1	6.46×10^{-09}
399	P	35	129,028	129,062	240,279	240,313	–1	6.46×10^{-09}
400	F	35	129,028	129,062	306,969	307,003	–1	6.46×10^{-09}
401	F	35	129,030	129,064	356,048	356,082	–1	6.46×10^{-09}
402	P	35	144,646	144,680	177,813	177,847	–1	6.46×10^{-09}
403	P	35	144,648	144,682	291,307	291,341	–1	6.46×10^{-09}
404	F	35	159,793	159,827	177,813	177,847	–1	6.46×10^{-09}
405	P	35	223,365	223,399	291,307	291,341	–1	6.46×10^{-09}
406	P	35	291,614	291,648	371,358	371,392	–1	6.46×10^{-09}
407	F	35	291,614	291,648	456,223	456,257	–1	6.46×10^{-09}
408	P	35	356,047	356,081	449,684	449,718	–1	6.46×10^{-09}
409	P	35	356,461	356,495	372,054	372,088	–1	6.46×10^{-09}
410	F	31	20,877	20,907	448,185	448,215	0	1.57×10^{-08}
411	F	31	34,334	34,364	44,144	44,174	0	1.57×10^{-08}
412	P	31	62,971	63,001	147,158	147,188	0	1.57×10^{-08}
413	P	31	62,971	63,001	430,185	430,215	0	1.57×10^{-08}
414	P	31	74,018	74,048	144,646	144,676	0	1.57×10^{-08}
415	F	31	74,018	74,048	159,797	159,827	0	1.57×10^{-08}
416	F	31	74,018	74,048	343,089	343,119	0	1.57×10^{-08}
417	F	31	74,018	74,048	460,969	460,999	0	1.57×10^{-08}

418	F	31	144,646	144,676	353,219	353,249	0	1.57×10^{-08}
419	P	31	159,797	159,827	353,219	353,249	0	1.57×10^{-08}
420	P	31	276,783	276,813	333,869	333,899	0	1.57×10^{-08}
421	F	31	280,087	280,117	371,678	371,708	0	1.57×10^{-08}
422	P	31	343,089	343,119	353,219	353,249	0	1.57×10^{-08}
423	P	31	353,219	353,249	460,969	460,999	0	1.57×10^{-08}
424	F	40	2,898	2,937	300,980	301,019	–3	1.60×10^{-08}
425	P	40	6,180	6,219	274,730	274,769	–3	1.60×10^{-08}
426	P	40	84,730	84,769	294,823	294,862	–3	1.60×10^{-08}
427	F	40	122,403	122,442	279,984	280,023	–3	1.60×10^{-08}
428	F	40	124,508	124,547	195,624	195,663	–3	1.60×10^{-08}
429	P	40	130,727	130,766	162,867	162,906	–3	1.60×10^{-08}
430	F	40	276,771	276,810	388,640	388,679	–3	1.60×10^{-08}
431	P	40	279,984	280,023	460,711	460,750	–3	1.60×10^{-08}
432	P	37	129,270	129,306	356,044	356,080	–2	2.30×10^{-08}
433	P	37	144,641	144,677	249,291	249,327	–2	2.30×10^{-08}
434	F	37	153,890	153,926	177,812	177,848	–2	2.30×10^{-08}
435	F	37	290,966	291,002	343,085	343,121	–2	2.30×10^{-08}
436	F	37	317,120	317,156	479,070	479,106	–2	2.30×10^{-08}
437	F	37	343,085	343,121	362,223	362,259	–2	2.30×10^{-08}
438	P	34	3,063	3,096	60,699	60,732	–1	2.51×10^{-08}
439	P	34	62,932	62,965	147,194	147,227	–1	2.51×10^{-08}
440	P	34	113,191	113,224	306,970	307,003	–1	2.51×10^{-08}
441	F	34	129,270	129,303	240,279	240,312	–1	2.51×10^{-08}
442	P	34	147,101	147,134	393,159	393,192	–1	2.51×10^{-08}
443	F	34	159,792	159,825	291,308	291,341	–1	2.51×10^{-08}
444	P	34	177,813	177,846	223,364	223,397	–1	2.51×10^{-08}
445	P	34	177,813	177,846	371,358	371,391	–1	2.51×10^{-08}
446	F	34	177,813	177,846	456,224	456,257	–1	2.51×10^{-08}
447	P	34	228,191	228,224	240,279	240,312	–1	2.51×10^{-08}
448	F	34	228,191	228,224	306,970	307,003	–1	2.51×10^{-08}
449	F	34	249,291	249,324	343,088	343,121	–1	2.51×10^{-08}
450	F	34	249,291	249,324	460,968	461,001	–1	2.51×10^{-08}
451	F	34	291,308	291,341	343,084	343,117	–1	2.51×10^{-08}
452	P	34	291,308	291,341	371,359	371,392	–1	2.51×10^{-08}
453	F	34	291,308	291,341	456,223	456,256	–1	2.51×10^{-08}
454	F	34	291,308	291,341	460,964	460,997	–1	2.51×10^{-08}
455	F	34	393,129	393,162	449,714	449,747	–1	2.51×10^{-08}
456	P	39	1,768	1,806	378,125	378,163	–3	5.93×10^{-08}
457	P	39	96,372	96,410	129,268	129,306	–3	5.93×10^{-08}
458	F	30	7,674	7,703	77,820	77,849	0	6.30×10^{-08}
459	F	30	63,018	63,047	260,063	260,092	0	6.30×10^{-08}
460	P	30	68,972	69,001	96,345	96,374	0	6.30×10^{-08}
461	P	30	74,018	74,047	223,364	223,393	0	6.30×10^{-08}
462	P	30	74,018	74,047	371,358	371,387	0	6.30×10^{-08}
463	F	30	74,018	74,047	456,228	456,257	0	6.30×10^{-08}
464	P	30	197,110	197,139	359,410	359,439	0	6.30×10^{-08}
465	F	30	204,969	204,998	435,844	435,873	0	6.30×10^{-08}
466	F	30	223,364	223,393	353,220	353,249	0	6.30×10^{-08}
467	F	30	288,669	288,698	294,077	294,106	0	6.30×10^{-08}
468	F	30	339,986	340,015	372,268	372,297	0	6.30×10^{-08}
469	F	30	353,220	353,249	371,358	371,387	0	6.30×10^{-08}
470	P	30	353,220	353,249	456,228	456,257	0	6.30×10^{-08}

471	F	30	356,039	356,068	374,386	374,415	0	6.30×10^{-08}
472	P	30	437,148	437,177	496,318	496,347	0	6.30×10^{-08}
473	F	36	17,002	17,037	334,368	334,403	–2	8.72×10^{-08}
474	P	36	104,857	104,892	298,960	298,995	–2	8.72×10^{-08}
475	F	36	177,813	177,848	291,615	291,650	–2	8.72×10^{-08}
476	F	36	267,920	267,955	363,065	363,100	–2	8.72×10^{-08}
477	F	36	477,569	477,604	499,689	499,724	–2	8.72×10^{-08}
478	F	33	3,063	3,095	59,946	59,978	–1	9.74×10^{-08}
479	P	33	68,968	69,000	356,018	356,050	–1	9.74×10^{-08}
480	F	33	68,968	69,000	393,130	393,162	–1	9.74×10^{-08}
481	P	33	74,613	74,645	447,220	447,252	–1	9.74×10^{-08}
482	P	33	113,191	113,223	356,048	356,080	–1	9.74×10^{-08}
483	P	33	183,312	183,344	309,788	309,820	–1	9.74×10^{-08}
484	F	33	196,729	196,761	302,725	302,757	–1	9.74×10^{-08}
485	F	33	228,192	228,224	356,048	356,080	–1	9.74×10^{-08}
486	P	33	240,279	240,311	287,641	287,673	–1	9.74×10^{-08}
487	F	33	240,279	240,311	449,685	449,717	–1	9.74×10^{-08}
488	F	33	248,973	249,005	335,311	335,343	–1	9.74×10^{-08}
489	F	33	249,292	249,324	291,619	291,651	–1	9.74×10^{-08}
490	F	33	287,641	287,673	306,971	307,003	–1	9.74×10^{-08}
491	F	33	290,966	290,998	291,309	291,341	–1	9.74×10^{-08}
492	F	33	291,309	291,341	362,223	362,255	–1	9.74×10^{-08}
493	P	33	306,971	307,003	449,685	449,717	–1	9.74×10^{-08}
494	F	33	356,469	356,501	373,820	373,852	–1	9.74×10^{-08}
495	P	38	9,782	9,819	135,608	135,645	–3	2.19×10^{-07}
496	P	38	52,873	52,910	93,225	93,262	–3	2.19×10^{-07}
497	P	38	53,958	53,995	464,167	464,204	–3	2.19×10^{-07}
498	F	38	103,667	103,704	113,187	113,224	–3	2.19×10^{-07}
499	F	38	260,064	260,101	434,056	434,093	–3	2.19×10^{-07}
500	F	38	290,962	290,999	456,220	456,257	–3	2.19×10^{-07}
501	F	38	362,219	362,256	456,220	456,257	–3	2.19×10^{-07}
502	F	38	434,056	434,093	460,855	460,892	–3	2.19×10^{-07}
503	P	38	437,174	437,211	496,280	496,317	–3	2.19×10^{-07}
504	P	35	3,590	3,624	290,966	291,000	–2	3.29×10^{-07}
505	P	35	3,590	3,624	362,223	362,257	–2	3.29×10^{-07}
506	P	35	23,001	23,035	247,501	247,535	–2	3.29×10^{-07}
507	P	35	67,922	67,956	418,998	419,032	–2	3.29×10^{-07}
508	F	35	74,014	74,048	177,813	177,847	–2	3.29×10^{-07}
509	F	35	96,374	96,408	287,639	287,673	–2	3.29×10^{-07}
510	P	35	101,707	101,741	356,461	356,495	–2	3.29×10^{-07}
511	P	35	103,670	103,704	129,029	129,063	–2	3.29×10^{-07}
512	F	35	128,855	128,889	268,277	268,311	–2	3.29×10^{-07}
513	P	35	144,646	144,680	290,966	291,000	–2	3.29×10^{-07}
514	P	35	144,646	144,680	362,223	362,257	–2	3.29×10^{-07}
515	F	35	159,793	159,827	290,966	291,000	–2	3.29×10^{-07}
516	F	35	159,793	159,827	362,223	362,257	–2	3.29×10^{-07}
517	P	35	177,813	177,847	353,219	353,253	–2	3.29×10^{-07}
518	P	35	240,279	240,313	306,969	307,003	–2	3.29×10^{-07}
519	P	35	281,751	281,785	408,902	408,936	–2	3.29×10^{-07}
520	P	35	330,989	331,023	412,671	412,705	–2	3.29×10^{-07}
521	P	32	3,590	3,621	249,291	249,322	–1	3.78×10^{-07}
522	P	32	17,006	17,037	276,775	276,806	–1	3.78×10^{-07}
523	F	32	159,796	159,827	249,291	249,322	–1	3.78×10^{-07}

524	F	32	375,167	375,198	453,480	453,511	–1	3.78×10^{-07}
525	P	37	4,719	4,755	268,859	268,895	–3	8.06×10^{-07}
526	P	37	26,051	26,087	334,316	334,352	–3	8.06×10^{-07}
527	P	37	52,900	52,936	302,407	302,443	–3	8.06×10^{-07}
528	P	37	93,225	93,261	359,248	359,284	–3	8.06×10^{-07}
529	F	37	119,049	119,085	386,844	386,880	–3	8.06×10^{-07}
530	F	37	119,049	119,085	391,156	391,192	–3	8.06×10^{-07}
531	F	37	177,816	177,852	249,291	249,327	–3	8.06×10^{-07}
532	P	37	236,837	236,873	473,793	473,829	–3	8.06×10^{-07}
533	P	37	302,407	302,443	359,274	359,310	–3	8.06×10^{-07}
534	F	37	365,350	365,386	442,696	442,732	–3	8.06×10^{-07}
535	P	34	20,846	20,879	422,799	422,832	–2	1.24×10^{-06}
536	F	34	67,922	67,955	201,279	201,312	–2	1.24×10^{-06}
537	P	34	96,375	96,408	449,685	449,718	–2	1.24×10^{-06}
538	P	34	103,670	103,703	287,641	287,674	–2	1.24×10^{-06}
539	F	34	103,670	103,703	449,684	449,717	–2	1.24×10^{-06}
540	F	34	103,671	103,704	129,270	129,303	–2	1.24×10^{-06}
541	P	34	103,671	103,704	228,191	228,224	–2	1.24×10^{-06}
542	F	34	131,195	131,228	147,101	147,134	–2	1.24×10^{-06}
543	P	34	223,364	223,397	290,966	290,999	–2	1.24×10^{-06}
544	P	34	223,364	223,397	362,223	362,256	–2	1.24×10^{-06}
545	F	34	249,292	249,325	290,970	291,003	–2	1.24×10^{-06}
546	F	34	249,292	249,325	362,227	362,260	–2	1.24×10^{-06}
547	P	34	281,833	281,866	447,266	447,299	–2	1.24×10^{-06}
548	P	34	290,966	290,999	371,358	371,391	–2	1.24×10^{-06}
549	P	34	315,194	315,227	374,395	374,428	–2	1.24×10^{-06}
550	P	34	362,223	362,256	371,358	371,391	–2	1.24×10^{-06}
551	P	31	3,061	3,091	124,461	124,491	–1	1.46×10^{-06}
552	F	31	16,999	17,029	333,869	333,899	–1	1.46×10^{-06}
553	P	31	20,849	20,879	434,397	434,427	–1	1.46×10^{-06}
554	F	31	74,018	74,048	249,292	249,322	–1	1.46×10^{-06}
555	F	31	77,817	77,847	390,280	390,310	–1	1.46×10^{-06}
556	F	31	110,642	110,672	183,336	183,366	–1	1.46×10^{-06}
557	P	31	122,259	122,289	183,314	183,344	–1	1.46×10^{-06}
558	F	31	199,791	199,821	305,888	305,918	–1	1.46×10^{-06}
559	P	31	223,364	223,394	249,291	249,321	–1	1.46×10^{-06}
560	F	31	223,791	223,821	390,274	390,304	–1	1.46×10^{-06}
561	F	31	243,316	243,346	360,679	360,709	–1	1.46×10^{-06}
562	P	31	249,291	249,321	371,358	371,388	–1	1.46×10^{-06}
563	F	31	249,291	249,321	456,227	456,257	–1	1.46×10^{-06}
564	P	31	249,292	249,322	353,219	353,249	–1	1.46×10^{-06}
565	P	31	280,940	280,970	348,254	348,284	–1	1.46×10^{-06}
566	F	31	348,254	348,284	358,321	358,351	–1	1.46×10^{-06}
567	F	36	6,180	6,215	17,412	17,447	–3	2.96×10^{-06}
568	F	36	34,463	34,498	315,249	315,284	–3	2.96×10^{-06}
569	P	36	60,699	60,734	147,099	147,134	–3	2.96×10^{-06}
570	F	36	96,347	96,382	268,863	268,898	–3	2.96×10^{-06}
571	F	36	119,103	119,138	391,214	391,249	–3	2.96×10^{-06}
572	F	36	153,891	153,926	343,085	343,120	–3	2.96×10^{-06}
573	F	36	153,891	153,926	460,965	461,000	–3	2.96×10^{-06}
574	P	36	156,177	156,212	340,953	340,988	–3	2.96×10^{-06}
575	F	36	177,813	177,848	290,966	291,001	–3	2.96×10^{-06}
576	F	36	177,813	177,848	362,223	362,258	–3	2.96×10^{-06}

577	P	36	187,302	187,337	340,953	340,988	–3	2.96×10^{-06}
578	F	36	239,777	239,812	297,824	297,859	–3	2.96×10^{-06}
579	F	36	268,863	268,898	356,019	356,054	–3	2.96×10^{-06}
580	P	36	334,379	334,414	388,633	388,668	–3	2.96×10^{-06}
581	P	33	3,067	3,099	183,312	183,344	–2	4.67×10^{-06}
582	F	33	68,225	68,257	464,868	464,900	–2	4.67×10^{-06}
583	F	33	68,968	69,000	449,715	449,747	–2	4.67×10^{-06}
584	F	33	88,107	88,139	500,124	500,156	–2	4.67×10^{-06}
585	P	33	96,376	96,408	113,191	113,223	–2	4.67×10^{-06}
586	F	33	96,376	96,408	129,030	129,062	–2	4.67×10^{-06}
587	F	33	96,376	96,408	228,192	228,224	–2	4.67×10^{-06}
588	P	33	136,525	136,557	300,699	300,731	–2	4.67×10^{-06}
589	R	33	177,386	177,418	177,386	177,418	–2	4.67×10^{-06}
590	F	33	177,813	177,845	291,309	291,341	–2	4.67×10^{-06}
591	P	33	240,279	240,311	356,048	356,080	–2	4.67×10^{-06}
592	F	33	249,232	249,264	249,275	249,307	–2	4.67×10^{-06}
593	P	33	293,497	293,529	394,733	394,765	–2	4.67×10^{-06}
594	F	33	317,203	317,235	460,001	460,033	–2	4.67×10^{-06}
595	F	33	326,891	326,923	434,379	434,411	–2	4.67×10^{-06}
596	P	33	330,997	331,029	407,452	407,484	–2	4.67×10^{-06}
597	P	30	17,612	17,641	118,557	118,586	–1	5.67×10^{-06}
598	F	30	60,699	60,728	183,315	183,344	–1	5.67×10^{-06}
599	F	30	75,479	75,508	76,794	76,823	–1	5.67×10^{-06}
600	P	30	82,347	82,376	254,701	254,730	–1	5.67×10^{-06}
601	P	30	96,343	96,372	437,636	437,665	–1	5.67×10^{-06}
602	F	30	96,366	96,395	374,385	374,414	–1	5.67×10^{-06}
603	F	30	113,196	113,225	240,284	240,313	–1	5.67×10^{-06}
604	P	30	124,464	124,493	309,780	309,809	–1	5.67×10^{-06}
605	P	30	131,194	131,223	393,164	393,193	–1	5.67×10^{-06}
606	P	30	186,187	186,216	223,760	223,789	–1	5.67×10^{-06}
607	F	30	192,762	192,791	239,377	239,406	–1	5.67×10^{-06}
608	F	30	239,996	240,025	495,997	496,026	–1	5.67×10^{-06}
609	F	30	281,924	281,953	297,850	297,879	–1	5.67×10^{-06}
610	F	30	304,683	304,712	387,468	387,497	–1	5.67×10^{-06}
611	P	30	317,603	317,632	318,838	318,867	–1	5.67×10^{-06}
612	F	30	333,870	333,899	334,366	334,395	–1	5.67×10^{-06}
613	P	30	374,386	374,415	449,697	449,726	–1	5.67×10^{-06}
614	P	30	447,314	447,343	479,308	479,337	–1	5.67×10^{-06}
615	P	35	3,590	3,624	153,891	153,925	–3	1.09×10^{-05}
616	F	35	53,009	53,043	359,383	359,417	–3	1.09×10^{-05}
617	P	35	60,057	60,091	374,395	374,429	–3	1.09×10^{-05}
618	F	35	119,103	119,137	386,902	386,936	–3	1.09×10^{-05}
619	F	35	122,254	122,288	147,100	147,134	–3	1.09×10^{-05}
620	P	35	129,269	129,303	372,054	372,088	–3	1.09×10^{-05}
621	P	35	144,646	144,680	153,891	153,925	–3	1.09×10^{-05}
622	F	35	153,891	153,925	159,793	159,827	–3	1.09×10^{-05}
623	P	35	197,139	197,173	359,383	359,417	–3	1.09×10^{-05}
624	P	35	231,622	231,656	420,925	420,959	–3	1.09×10^{-05}
625	F	35	249,292	249,326	291,313	291,347	–3	1.09×10^{-05}
626	P	35	260,077	260,111	430,141	430,175	–3	1.09×10^{-05}
627	F	35	280,357	280,391	310,253	310,287	–3	1.09×10^{-05}
628	F	35	371,522	371,556	430,220	430,254	–3	1.09×10^{-05}
629	P	35	437,194	437,228	496,263	496,297	–3	1.09×10^{-05}

630	F	32	25,571	25,602	401,338	401,369	–2	1.76×10^{-05}
631	P	32	59,947	59,978	183,316	183,347	–2	1.76×10^{-05}
632	P	32	67,923	67,954	351,087	351,118	–2	1.76×10^{-05}
633	P	32	156,406	156,437	410,380	410,411	–2	1.76×10^{-05}
634	P	32	201,280	201,311	351,087	351,118	–2	1.76×10^{-05}
635	F	32	202,280	202,311	433,611	433,642	–2	1.76×10^{-05}
636	P	32	241,504	241,535	268,860	268,891	–2	1.76×10^{-05}
637	P	32	268,863	268,894	393,130	393,161	–2	1.76×10^{-05}
638	F	32	277,459	277,490	375,170	375,201	–2	1.76×10^{-05}
639	F	32	302,380	302,411	464,563	464,594	–2	1.76×10^{-05}
640	P	32	312,944	312,975	473,827	473,858	–2	1.76×10^{-05}
641	P	32	345,400	345,431	459,895	459,926	–2	1.76×10^{-05}
642	F	32	351,087	351,118	419,000	419,031	–2	1.76×10^{-05}
643	P	34	3,063	3,096	393,159	393,192	–3	3.97×10^{-05}
644	P	34	84,741	84,774	294,818	294,851	–3	3.97×10^{-05}
645	P	34	103,670	103,703	356,048	356,081	–3	3.97×10^{-05}
646	F	34	103,671	103,704	240,279	240,312	–3	3.97×10^{-05}
647	P	34	103,671	103,704	306,970	307,003	–3	3.97×10^{-05}
648	P	34	113,191	113,224	372,054	372,087	–3	3.97×10^{-05}
649	F	34	129,029	129,062	372,054	372,087	–3	3.97×10^{-05}
650	P	34	142,618	142,651	156,030	156,063	–3	3.97×10^{-05}
651	F	34	147,101	147,134	309,784	309,817	–3	3.97×10^{-05}
652	P	34	153,891	153,924	223,364	223,397	–3	3.97×10^{-05}
653	P	34	153,891	153,924	371,358	371,391	–3	3.97×10^{-05}
654	F	34	153,891	153,924	456,224	456,257	–3	3.97×10^{-05}
655	P	34	177,441	177,474	187,297	187,330	–3	3.97×10^{-05}
656	P	34	190,948	190,981	316,257	316,290	–3	3.97×10^{-05}
657	F	34	212,118	212,151	473,795	473,828	–3	3.97×10^{-05}
658	F	34	228,191	228,224	372,054	372,087	–3	3.97×10^{-05}
659	P	34	341,534	341,567	341,542	341,575	–3	3.97×10^{-05}
660	P	34	426,494	426,527	460,078	460,111	–3	3.97×10^{-05}
661	P	34	430,133	430,166	448,551	448,584	–3	3.97×10^{-05}
662	P	31	2,564	2,594	427,789	427,819	–2	6.59×10^{-05}
663	P	31	6,189	6,219	274,730	274,760	–2	6.59×10^{-05}
664	F	31	7,665	7,695	177,432	177,462	–2	6.59×10^{-05}
665	P	31	11,545	11,575	262,614	262,644	–2	6.59×10^{-05}
666	F	31	16,755	16,785	408,995	409,025	–2	6.59×10^{-05}
667	F	31	87,217	87,247	501,976	502,006	–2	6.59×10^{-05}
668	F	31	95,212	95,242	136,249	136,279	–2	6.59×10^{-05}
669	F	31	96,345	96,375	186,149	186,179	–2	6.59×10^{-05}
670	F	31	98,919	98,949	375,171	375,201	–2	6.59×10^{-05}
671	F	31	135,110	135,140	460,729	460,759	–2	6.59×10^{-05}
672	F	31	157,087	157,117	163,763	163,793	–2	6.59×10^{-05}
673	F	31	163,763	163,793	317,083	317,113	–2	6.59×10^{-05}
674	F	31	243,468	243,498	360,838	360,868	–2	6.59×10^{-05}
675	P	31	260,063	260,093	279,827	279,857	–2	6.59×10^{-05}
676	P	31	265,601	265,631	289,867	289,897	–2	6.59×10^{-05}
677	P	31	276,025	276,055	432,901	432,931	–2	6.59×10^{-05}
678	P	31	291,586	291,616	489,748	489,778	–2	6.59×10^{-05}
679	F	31	294,483	294,513	426,268	426,298	–2	6.59×10^{-05}
680	R	31	341,540	341,570	341,540	341,570	–2	6.59×10^{-05}
681	P	31	356,019	356,049	437,631	437,661	–2	6.59×10^{-05}
682	P	31	372,050	372,080	442,111	442,141	–2	6.59×10^{-05}

683	F	31	377,393	377,423	429,385	429,415	–2	6.59×10^{-05}
684	F	31	393,131	393,161	437,631	437,661	–2	6.59×10^{-05}

F: forward repeats; P: palindromic repeats; R: reverse repeats.

Table S4. RNA editing sites identified in mitochondrial PCGs from transcriptome data.

Gene	Editing Sites	Reference	Alternative	Editing Efficiency
atp1	178	G	A	0.1946
atp1	186	A	G	0.7676
atp1	285	C	U	0.5977
atp1	642	A	G	0.3951
atp1	1064	C	U	1
atp1	1178	C	U	0.9032
atp1	1292	C	U	1
atp1	1415	C	U	0.95
atp1	1490	C	U	0.923
atp1	1497	A	U	1
atp1	1517	C	U	1
atp1	1519	A	C	1
atp4	34	C	U	1
atp4	35	C	U	1
atp4	38	C	U	1
atp4	68	C	U	0.7058
atp4	97	C	U	1
atp4	194	C	U	0.82
atp4	206	C	U	1
atp4	227	C	U	0.923
atp4	230	C	U	0.95
atp4	285	C	U	0.4
atp4	374	C	U	0.9733
atp4	386	C	U	0.9418
atp6	76	C	U	1
atp6	268	C	U	1
atp6	275	C	U	1
atp6	293	C	U	1
atp6	301	C	U	1
atp6	308	C	U	1
atp6	440	C	U	1
atp6	499	C	U	1
atp6	502	C	U	1
atp6	524	C	U	1
atp6	566	C	U	1
atp8	30	C	U	0.4375
atp8	47	C	U	0.9714
atp8	58	C	U	1
atp8	76	C	U	0.975
atp8	77	C	U	0.8863
atp8	171	C	G	0.1686
atp8	179	C	G	0.1219
atp8	182	A	G	0.2307
atp8	215	G	U	0.25
atp8	303	A	C	1
atp8	452	C	U	0.9756

ccmB	28	C	U	0.8333
ccmB	43	C	U	0.8333
ccmB	71	C	U	0.8333
ccmB	80	C	U	0.5
ccmB	87	C	U	0.6
ccmB	128	C	U	1
ccmB	137	C	U	1
ccmB	138	C	U	1
ccmB	148	C	U	0.6666
ccmB	149	C	U	0.6666
ccmB	154	C	U	0.6666
ccmB	160	C	U	0.6666
ccmC	5	C	U	0.6
ccmC	54	C	U	0.3636
ccmC	76	C	U	1
ccmC	103	C	U	1
ccmC	115	C	U	0.8181
ccmC	161	C	U	0.8181
ccmC	179	C	U	0.8333
ccmC	184	C	U	0.8
ccmC	281	C	U	1
ccmC	521	C	U	1
ccmC	548	C	U	1
ccmC	568	C	U	1
ccmC	575	C	U	1
ccmC	605	C	U	1
ccmC	608	C	U	1
ccmC	614	C	U	1
ccmC	619	C	U	1
ccmC	624	C	U	1
ccmC	650	C	U	1
ccmC	656	C	U	1
ccmC	673	C	U	1
ccmFc	146	C	U	1
ccmFc	151	C	U	1
ccmFc	1237	C	U	1
ccmFn	98	C	U	1
ccmFn	137	C	U	0.6666
ccmFn	142	C	U	0.6666
ccmFn	151	C	U	0.6666
ccmFn	248	C	U	1
ccmFn	782	C	U	1
ccmFn	794	C	U	1
ccmFn	809	C	U	0.6666
ccmFn	958	C	U	1
ccmFn	1276	C	U	1
ccmFn	1304	C	U	1
cob	53	C	U	0.9444
cob	118	C	U	1
cob	286	C	U	1
cob	298	C	U	1
cob	325	C	U	1
cob	358	C	U	1

cob	407	C	U	1
cob	568	C	U	1
cob	737	C	U	1
cob	808	C	U	1
cob	853	C	U	1
cob	908	C	U	1
cob	982	C	U	1
cob	1124	C	U	1
cob	1160	C	U	0.25
cox1	18	C	U	0.1265
cox1	233	C	U	0.8965
cox1	245	C	U	0.9
cox1	443	C	U	1
cox1	506	C	U	1
cox1	542	C	U	0.8888
cox1	659	C	U	1
cox1	706	C	U	1
cox1	737	C	U	1
cox1	752	C	U	1
cox1	836	C	U	1
cox1	1177	C	U	0.909
cox1	1393	C	U	1
cox1	1396	C	U	1
cox1	1424	C	U	1
cox2	138	C	U	0.4117
cox2	161	C	U	1
cox2	163	C	U	0.875
cox2	253	C	U	1
cox2	379	C	U	1
cox2	443	C	U	1
cox2	460	C	U	0.1764
cox2	476	C	U	1
cox2	544	C	U	1
cox2	557	C	U	0.9117
cox2	581	C	U	1
cox2	632	C	U	1
cox2	721	C	U	1
cox2	742	C	U	1
cox3	15	G	A	1
cox3	112	C	U	1
cox3	263	C	U	0.8965
cox3	289	C	U	1
cox3	304	C	U	0.9807
cox3	311	C	U	0.9555
cox3	314	C	U	1
cox3	388	C	U	0.9344
cox3	413	C	U	1
cox3	574	T	G	1
cox3	754	C	U	1
cox3	764	C	U	1
matR	43	C	U	1
matR	166	C	U	0.2272
matR	193	C	U	0.7

matR	236	C	U	0.6666
matR	326	C	U	0.9032
matR	413	C	U	0.8333
matR	1064	C	U	0.375
matR	1679	C	U	0.8235
matR	1700	C	U	0.9411
matR	1720	C	U	0.875
matR	1734	C	U	0.9444
matR	1756	C	U	0.875
matR	1826	C	U	1
matR	1844	C	U	1
mttB	26	C	U	0.6666
mttB	59	C	U	1
mttB	64	C	U	1
mttB	112	C	U	0.6666
mttB	128	C	U	1
mttB	188	C	U	1
mttB	200	C	U	1
mttB	202	C	U	0.6666
mttB	252	C	U	0.6666
mttB	346	C	U	1
mttB	373	C	U	0.6666
mttB	374	C	U	0.6666
mttB	376	C	U	1
mttB	437	C	U	1
mttB	505	C	U	1
mttB	519	C	U	0.75
mttB	541	C	U	1
mttB	548	C	U	0.8
mttB	610	C	U	1
mttB	616	C	U	0.8888
mttB	660	C	U	1
mttB	667	C	U	0.8181
mttB	672	C	U	0.6666
mttB	683	C	U	0.6666
mttB	713	C	U	1
nad1	135	G	U	0.3333
nad1	215	C	U	1
nad1	436	C	U	1
nad1	490	C	U	0.6666
nad1	492	C	U	0.6666
nad1	493	C	U	1
nad1	500	C	U	0.6666
nad1	536	C	U	1
nad1	537	C	U	0.8
nad1	573	C	U	1
nad1	580	C	U	1
nad1	635	C	U	0.375
nad1	674	C	U	0.3846
nad1	722	C	U	1
nad1	731	C	U	0.8888
nad1	737	C	U	0.8888
nad1	739	C	U	0.2222

nad1	740	C	U	0.8888
nad1	752	C	U	0.625
nad1	820	C	U	0.2222
nad1	827	C	U	0.75
nad1	895	C	U	1
nad1	925	C	U	0.909
nad1	934	C	U	0.9
nad1	950	C	U	0.8
nad2	26	C	U	0.625
nad2	362	C	A	0.3636
nad2	367	C	U	0.5
nad2	370	A	U	0.3333
nad2	394	C	U	0.6666
nad2	401	C	U	0.2857
nad2	428	C	U	0.25
nad2	431	A	U	0.375
nad2	435	T	A	0.375
nad2	440	G	A	0.4
nad2	453	C	U	0.3571
nad2	455	C	A	0.3571
nad2	461	C	U	0.4375
nad2	497	C	U	0.2857
nad2	523	C	U	0.5
nad2	527	C	U	0.4285
nad2	529	G	A	0.5714
nad2	662	C	U	0.75
nad2	788	C	U	1
nad2	800	C	U	1
nad2	809	C	U	1
nad2	920	C	U	1
nad2	928	C	U	0.8888
nad2	958	C	U	1
nad2	962	C	U	1
nad2	1127	C	U	0.4285
nad2	1246	C	U	0.9
nad2	1276	C	U	0.8333
nad2	1298	C	U	1
nad2	1400	C	U	1
nad2	1403	C	U	1
nad2	1408	C	U	1
nad2	1409	C	U	0.7727
nad2	1416	C	U	0.8571
nad2	1457	C	U	1
nad3	43	C	U	1
nad3	44	C	U	1
nad3	61	C	U	1
nad3	62	C	U	1
nad3	80	C	U	0.75
nad3	208	C	U	0.4285
nad3	215	C	U	0.375
nad3	230	C	U	1
nad3	247	C	U	1
nad3	266	C	U	0.4285

nad3	275	C	U	0.4285
nad3	317	C	U	1
nad3	349	C	U	1
nad4	158	C	U	0.5
nad4	164	C	U	0.6666
nad4	166	C	U	0.6666
nad4	197	C	U	0.5
nad4	317	C	U	0.75
nad4	362	C	U	0.75
nad4	368	C	U	0.75
nad4	376	C	U	0.75
nad4	403	C	U	0.8
nad4	416	C	U	0.6666
nad4	433	C	U	0.6666
nad4	436	C	U	0.6666
nad4	437	C	U	0.6666
nad4	462	G	C	1
nad4	608	C	U	0.5
nad4	659	C	U	1
nad4	856	C	U	1
nad4	857	C	U	1
nad4	896	C	U	1
nad4	1006	C	U	1
nad4	1010	C	U	1
nad4	1033	C	U	1
nad4	1109	C	U	1
nad4	1129	C	U	1
nad4	1148	C	U	1
nad4	1205	C	U	1
nad4	1354	C	U	1
nad4	1373	C	U	1
nad4	1400	C	G	0.75
nad4	1406	C	U	0.75
nad4	1418	C	U	0.75
nad4	1433	C	U	0.5
nad4	1434	C	U	0.75
nad4L	150	C	U	0.4347
nad4L	196	C	U	0.0769
nad5	155	C	U	0.7142
nad5	229	G	U	0.6666
nad5	242	C	U	1
nad5	358	C	U	1
nad5	374	C	U	1
nad5	398	C	U	1
nad5	494	C	U	0.6363
nad5	539	C	U	0.3333
nad5	548	C	U	0.5625
nad5	553	C	U	0.4285
nad5	598	C	U	0.6666
nad5	608	C	U	1
nad5	629	C	U	0.8571
nad5	725	C	U	1
nad5	764	C	U	1

nad5	835	C	U	1
nad5	863	C	U	1
nad5	875	C	U	1
nad5	1469	C	U	1
nad5	1529	C	U	1
nad5	1547	C	U	1
nad5	1559	C	U	1
nad5	1568	C	U	0.8888
nad5	1589	C	U	1
nad5	1895	C	U	1
nad5	1897	C	U	1
nad5	1937	C	U	1
nad6	26	C	U	1
nad6	53	C	U	0.6666
nad6	59	C	A	0.3333
nad6	69	G	A	0.4
nad6	103	C	U	1
nad6	109	A	G	0.75
nad6	149	C	U	1
nad6	152	T	C	0.8
nad6	186	C	U	0.3333
nad6	191	C	U	0.6666
nad6	289	C	U	1
nad6	316	G	U	0.25
nad6	393	A	G	1
nad6	395	G	A	1
nad6	471	T	A	1
nad7	44	C	U	0.8571
nad7	45	C	U	0.4
nad7	77	C	U	0.7674
nad7	83	C	U	0.75
nad7	99	C	U	0.1555
nad7	137	C	U	0.8571
nad7	144	G	U	0.8
nad7	145	A	U	0.1794
nad7	244	C	U	0.9259
nad7	251	C	U	0.8823
nad7	315	C	U	0.3684
nad7	316	C	U	1
nad7	335	C	U	0.8372
nad7	344	C	U	0.7727
nad7	383	C	U	0.8648
nad7	531	C	U	0.3396
nad7	533	C	U	0.9019
nad7	578	C	U	0.9636
nad7	679	C	U	0.5777
nad7	698	C	U	0.909
nad7	724	C	U	1
nad7	734	C	U	1
nad7	739	C	U	0.9428
nad7	740	C	U	0.9705
nad7	769	C	U	0.8648
nad7	789	C	U	0.2439

nad7	924	G	C	0.826
nad7	926	C	U	0.6315
nad7	944	C	U	0.8166
nad7	963	C	U	0.1896
nad7	1050	C	U	0.8153
nad7	1057	C	U	0.9482
nad7	1079	C	U	0.9677
nad7	1103	C	U	0.9365
nad7	1124	C	U	0.9166
nad7	1166	C	U	0.9772
nad9	12	A	G	0.6666
nad9	27	G	A	0.6666
nad9	29	C	U	1
nad9	39	T	A	0.6666
nad9	50	C	U	1
nad9	104	C	U	1
nad9	132	T	C	0.4
nad9	235	C	U	1
nad9	261	C	U	0.5
nad9	265	C	U	1
nad9	305	C	U	1
nad9	335	C	U	1
nad9	367	C	U	0.4
nad9	376	C	U	1
rpl10	83	C	U	0.2777
rpl10	101	C	U	0.8
rpl10	134	C	U	0.8076
rpl10	155	C	U	0.5769
rpl10	239	C	U	0.6818
rpl10	314	C	U	0.4375
rpl16	102	C	U	0.6923
rpl16	104	C	U	0.9615
rpl16	164	C	U	0.96
rpl16	223	C	U	0.9444
rpl16	312	C	U	0.4
rpl16	326	C	U	0.84
rpl16	392	C	U	0.931
rpl16	398	C	U	0.9333
rpl16	405	C	U	0.3076
rpl5	7	C	U	0.5
rpl5	35	C	U	1
rpl5	47	C	U	1
rpl5	64	C	U	1
rpl5	92	C	U	1
rpl5	166	C	U	1
rpl5	169	C	U	1
rpl5	317	C	U	0.8857
rpl5	329	C	U	0.9459
rpl5	512	C	U	1
rpl5	515	C	U	1
rps1	26	C	U	0.6666
rps1	108	G	C	0.1999
rps1	161	C	U	0.5454

rps1	212	C	U	0.6428
rps1	251	G	A	1
rps1	537	T	A	1
rps1	552	C	U	0.1538
rps1	622	C	U	0.25
rps12	71	C	U	1
rps12	104	C	U	1
rps12	146	C	U	1
rps12	196	C	U	1
rps12	284	C	U	1
rps12	285	C	U	0.1999
rps13	26	C	U	1
rps13	56	C	U	1
rps13	100	C	U	1
rps13	256	C	U	1
rps13	287	C	U	0.8
rps14	143	C	U	0.1999
rps3	64	C	U	0.7804
rps3	92	C	U	0.9629
rps3	115	C	A	1
rps3	318	C	U	0.1176
rps3	512	C	U	0.8541
rps3	890	C	U	0.1851
rps3	1031	C	U	0.8372
rps3	1235	G	U	1
rps3	1361	C	U	0.1176
rps3	1364	C	U	1
rps3	1502	C	U	1
rps3	1506	A	C	0.125
rps3	1518	T	A	0.3095
rps3	1519	G	A	0.3249
rps3	1550	T	C	0.2916
rps3	1555	C	U	0.8571
rps3	1588	C	U	0.8545
rps3	1648	T	A	0.3333
rps4	35	C	U	0.5
rps4	61	C	U	1
rps4	145	C	U	0.7
rps4	176	C	U	0.9
rps4	196	C	U	0.8888
rps4	205	C	U	0.8571
rps4	219	C	U	0.375
rps4	269	C	U	1
rps4	278	C	U	0.5714
rps4	290	C	U	0.7142
rps4	347	C	U	1
rps4	446	C	U	0.25
rps4	494	C	U	0.8
rps4	797	C	U	0.3181
rps4	926	C	U	0.8333
rps4	947	C	U	1
rps4	962	C	U	1
rps4	1022	C	U	1

rps4	1027	C	U	0.8888
sdh4	29	C	U	1
sdh4	39	C	U	1
sdh4	153	C	U	0.4444
sdh4	155	C	U	1
sdh4	203	C	U	1
sdh4	259	C	U	1
sdh4	348	C	U	0.5789
sdh4	353	C	U	0.8421
sdh4	359	C	U	0.5
